# Supporting surveillance capacity for antimicrobial resistance: Laboratory capacity strengthening for drug resistant infections in low and middle income countries

**DOI:** 10.12688/wellcomeopenres.12523.1

**Published:** 2017-09-26

**Authors:** Anna C. Seale, Coll Hutchison, Silke Fernandes, Nicole Stoesser, Helen Kelly, Brett Lowe, Paul Turner, Kara Hanson, Clare I.R. Chandler, Catherine Goodman, Richard A. Stabler, J. Anthony G. Scott

**Affiliations:** 1 College of Health and Medical Sciences, Haramaya University, Harar, Ethiopia; 2 London School of Hygiene & Tropical Medicine, London, WC1E 7HT, UK; 3 Nuffield Department of Medicine, University of Oxford, Oxford, OX3 7BN, UK; 4 KEMRI-Wellcome Trust Research Programme, Kilifi, Kenya; 5 Cambodia Oxford Medical Research Unit, Siem Reap, Cambodia

**Keywords:** antimicrobial, antibiotic resistance, drug, infection, surveillance

## Abstract

Development of antimicrobial resistance (AMR) threatens our ability to treat common and life threatening infections. Identifying the emergence of AMR requires strengthening of surveillance for AMR, particularly in low and middle-income countries (LMICs) where the burden of infection is highest and health systems are least able to respond. This work aimed, through a combination of desk-based investigation, discussion with colleagues worldwide, and visits to three contrasting countries (Ethiopia, Malawi and Vietnam), to map and compare existing models and surveillance systems for AMR, to examine what worked and what did not work.

Current capacity for AMR surveillance varies in LMICs, but and systems in development are focussed on laboratory surveillance. This approach limits understanding of AMR and the extent to which laboratory results can inform local, national and international public health policy. An integrated model, combining clinical, laboratory and demographic surveillance in sentinel sites is more informative and costs for clinical and demographic surveillance are proportionally much lower.

The speed and extent to which AMR surveillance can be strengthened depends on the functioning of the health system, and the resources available. Where there is existing laboratory capacity, it may be possible to develop 5-20 sentinel sites with a long term view of establishing comprehensive surveillance; but where health systems are weaker and laboratory infrastructure less developed, available expertise and resources may limit this to 1-2 sentinel sites. Prioritising core functions, such as automated blood cultures, reduces investment at each site. Expertise to support AMR surveillance in LMICs may come from a variety of international, or national, institutions. It is important that these organisations collaborate to support the health systems on which AMR surveillance is built, as well as improving technical capacity specifically relating to AMR surveillance. Strong collaborations, and leadership, drive successful AMR surveillance systems across countries and contexts.

## Introduction

Since the introduction of antibiotics into medicine in the 1940s, they have provided dependable treatment for many infectious diseases. The emergence of antimicrobial resistance (AMR) and related drug resistant infections (DRIs) challenges this ^1^. One of the early priorities identified to tackle the emergence of AMR and DRIs is to strengthen surveillance of AMR in low and middle income countries (LMICs) ^2^.

Despite LMICs having the highest incidence of infection (and associated mortality and morbidity) there are significant gaps in our understanding of the aetiology and incidence of infectious diseases in these contexts ^3, 4^. This constrains national governments and international organisations in their efforts to detect evolving trends and emerging threats.

Data on the true prevalence of AMR in LMICs are limited. In 2014, the World Health Organization (WHO) summarized the most recent information on AMR surveillance for a selected set of nine bacteria–antibacterial drug combinations of public health importance from 129 Member States ^5^. Among WHO regions, the greatest volume of country-level data were obtained from the European Region and the Region of the Americas, where longstanding regional surveillance and collaboration exist. In contrast, data from LMIC were underrepresented, and in some countries, lacking. However, there is no evidence to suggest that the burden of AMR for many common pathogens in LMICs is not increasing in line with the rest of the world ^5^, and can be the place of emergence ^6^. Understanding current capacity for AMR surveillance in LMICs is important, as well as considering how to develop capacity in different settings and contexts, and their requisite costs.

Modelled estimates put the number of AMR attributable deaths per year in Europe at 25,000 with €1.5 billion in associated health care costs and productivity losses ^7^; in the United States there are 23,000 deaths per year attributable to AMR at a direct cost of US$20 billion and an indirect cost of US$35 billion in productivity losses ^8, 9^. There are no reliable estimates available on the economic losses due to AMR available from LMICs, but they are likely to be considerable. Despite the economic losses identified in high income countries, funding to tackle AMR has, until recently, been limited. This is in part because costs associated with establishing and running AMR surveillance were considered to be high. With a view to informing future capacity building for AMR surveillance in LMIC settings, this work aimed to characterise the requirements for achieving capacity to detect the emergence of AMR at national levels. The specific objectives were:

i) To map and compare existing models for laboratory system strengthening (for AMR surveillance), including the identification of specific contexts and geographical settings where certain models have been more or less successful, analysis of challenges and barriers in different geographical and sociodemographic contexts, consideration of costs and sustainability and assessment of governance issues, including the ability to share data.

ii) To map and compare existing AMR surveillance systems in at least three countries, identifying different approaches for monitoring emergence and spread of resistance in different countries or regional settings, including the range of baseline data gathered, the best models and mechanisms for surveillance, capacity strengthening and training in the different country or regional settings.

A fully formatted report is included as Supplementary File 1.

## Methods

**Figure 1.**
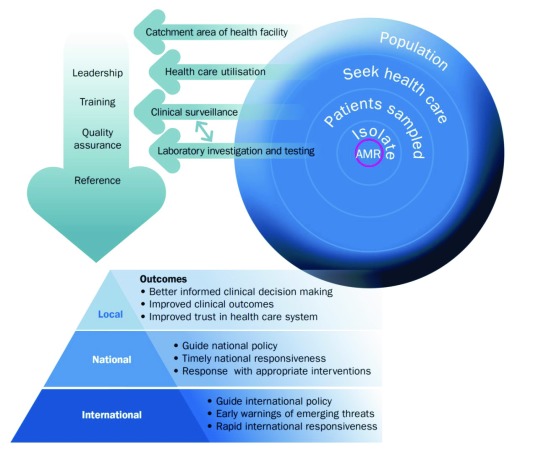
Antimicrobial resistance conceptual model. At a national level, there is a need for leadership to support training, and quality, with a reference laboratory function. At a site an integrated model includes development of laboratory capacity, clinical surveillance, health care utilisation surveys and census or enumeration data to determine the population catchment. This provides the framework for local, national and international public health action.

A team with expertise in health systems, health economics, anthropology, microbiology, laboratory capacity, surveillance, and epidemiology was brought together. This group discussed and developed an initial outline of a conceptual model for AMR surveillance on which to frame the desk based analysis, case studies and costing of AMR ( Figure 1).

### Desk based analysis and conceptual model

A desk-based analysis of capacity for AMR surveillance in Sub-Saharan Africa and South-east Asia was done, first by reviewing the WHO’s AMR report and then looking at those countries that had been able to contribute data, and identifying which components of the conceptual model they had put in place (or other) to do this. The challenges faced in these systems were also considered. Where there were few or no data in WHO sub-regions, examples of other surveillance systems that had been established, outside of the context of AMR surveillance were reviewed.

### Case studies

Site visits were made to Malawi, Ethiopia and Vietnam, and capacity in these countries reviewed according to the components included in the conceptual model and the functions that would be needed to improve/develop AMR surveillance, as well as the strengths of the systems and models in place. This work involved extensive review of relevant guidelines and health system and policy literature, as well as discussions with in-country personnel, including laboratory staff, policy-makers, clinicians, microbiologists, veterinarians and epidemiologists.

### Analytic framework

We identified the functions, sub-functions and activities needed for AMR surveillance at a national and site level, and defined four levels of AMR surveillance capacity involving increasing complexity. Capacity at Level 1 represents the most limited system, with incomplete clinical data, minimal clinical investigation and no laboratory testing. At level 2 there is some laboratory testing but no standardization of processes in place. Level 3 represents a core surveillance system with standardized processes, focusing on invasive disease in children under five years and the identification and antimicrobial susceptibility testing of WHO priority pathogens ( *Escherichia coli, Klebsiella pneumoniae, Staphylococcus aureus, Streptococcus pneumoniae, Acinetobacter baumannii, Salmonella spp., Shigella spp., Neisseria gonorrhoeae*). Level 4 represents extended AMR surveillance capacity, with capacity to include AMR surveillance from all patients and all sample types. The four functions at the sentinel site level were 1) clinical admission assessment & investigation; 2) isolate identification and susceptibility testing; 3) isolate storage (local) & referral to reference laboratory; and 4) data system and review. At the national level these functions were 1) leadership; 2) training; 3) quality assurance; and 4) national reference laboratory. Each function was further broken down into sub-functions; for example “clinical admission assessment & investigation” has four sub-functions, namely i) clinical admission assessment; ii) clinical data; iii) clinical investigation; and iv) training and quality assurance, all describing the different activities relevant for clinical surveillance. Components are detailed in Table 1 and Table 2.

**Table 1.** Functions and sub-functions by level of AMR surveillance sophistication at a site.

Level of surveillance sophistication increasing from left to right					
		Level 1	Level 2	Level 3 - CORE	Level 4 - EXTENDED
**Aim of AMR** **surveillance**		Limited	Surveillance data drive local policy	Surveillance data drive national policy and international policy	Surveillance data drive national policy and international policy
**Functions**	**Sub functions**				
**Clinical admission** **assessment and** **investigation**	***Clinical*** ***admission*** ***assessment***	Clinical history and examination incomplete	Clinical history and examination, but no algorithms in use	Systematic clinical history and examination according to clinical algorithms in core patient group (< 5 years)	Systematic clinical history and examination according to clinical algorithms in all target patient groups
	***Clinical data***	Documentation incomplete	Paper based documentation or electronic system without linkage	Documentation into paper system using unique alphanumeric identifier which links to lab isolates	Standardised documentation into electronic system using unique alphanumeric identifier which links to lab isolates
	***Clinical*** ***investigation***	Clinical investigations ad-hoc	Clinical investigation based on clinical findings but not standardised	Standardised clinical investigation based on clinical findings (fever) in core patient group	Standardised clinical investigation based on clinical findings (fever) in all target patient groups
	***Training*** ***and quality*** ***assurance***	Not done	Ad hoc	Routine training to surveillance SOPs	Quality assurance of clinical data with external assessment
**Isolate identification** **and susceptibility** **testing**	***Sample*** ***transport from*** ***clinical to*** ***laboratory***	Samples not transported routinely	Samples transported, no standards in place to guide process	Samples transported according to local standard operating procedures	Samples transported according to international standards for biosafety
	***Sample*** ***registration***	No registration system	Paper based registration (logbook)	Local lab data system with manual linkage to clinical data on site	Local lab data system linked to clinical data with aggregation to national network (automated)
	***Culture***	No blood culture	No blood cultures	Automated blood culture, with processing the WHO 9 priority pathogens according to SOPs	Automated blood culture as well as cerebrospinal fluid, urine, stool and swab culture, processing all isolates according to SOPs
	***Susceptibility*** ***testing***	Not done	Done but not according to defined standards and/or without SOPs	According to SOPs for WHO 9 priority isolates; qualitative testing	According to SOPs for all isolates
	***Training*** ***and quality*** ***assurance***	Not done	Ad hoc	Routine training to surveillance SOPs	Quality assurance of lab with external assessment; accreditation to ISO 15189 standards
**Isolate storage** **(local) and referral to** **reference lab**	***Storage of*** ***isolates***	No biorepository, or no documentation of stored samples.	Biorepository of isolates, paper-based database with storage of at least a subset of resistant isolates defined by local policy (-20 or -80C)	Biorepository of isolates, with paper or electronic database with storage of all resistant isolates (-20 or -80C) defined by national policy	Biorepository of isolates, with electronic database of all stored resistant isolates defined by national and/or international policy, maintained at -80C.
	***Transport to*** ***reference lab***	Isolates are not transferred to reference lab	Isolates are transferred to ref lab on an ad-hoc basis	Isolates are transferred to ref lab at least annually but not according to defined SOPs	Isolates are transferred to ref lab at least annually according to international standards for biosafety
	***Training*** ***and quality*** ***assurance***	Not done	Ad hoc	Routine training for isolate storage and transport SOPs	Routine training for isolate storage and transport SOPs; Quality assurance of lab biorepository with external assessment
**Data system and** **review**	***Data system***	None	Not done	Local lab data system with manual linkage to clinical data on site; system not linked to national network; paper based or electronic biorepository database that is not linked	Local lab data system electronically linked to clinical data and automatically linked to national network
	***Data use***	Limited	Data used for individual clinical care; hospital review for treatment guidelines	Submit to national network	Submit to international network (through country level surveillance)
	***Data linkage***	No linkage	No linkage	Manual linkage	Automated linkage
	***Data*** ***governance***	No data sharing policy	Local data sharing policy (in-hospital)	Data sharing policy and agreements in place in collaboration with the Ministry of Health and/or national public health institute (see national level)	Data sharing policy and agreements in place in collaboration with the Ministry of Health and/or national public health institute (see national level)

**Table 2.** Functions and sub-functions by level of antimicrobial resistance (AMR) surveillance sophistication at national level.

Level of surveillance sophistication increasing from left to right					
		Level 1	Level 2	Level 3 - CORE	Level 4 - EXTENDED
**Aim of AMR** **surveillance**		Limited	Surveillance data drive local policy	Surveillance data drive national policy and international policy	Surveillance data drive national policy and international policy
**Functions**	**Sub functions**				
**Leadership**	***Data analysis***	None - no national coordinating body	National coordinating body but limited national data	National coordinating body reviews aggregated data with annual report	National coordinating body reviews aggregated data with annual report
	***Data*** ***governance***	None - no national coordinating body	National coordinating body but limited national data	National standards for data governance, legislation	National standards for data governance, legislation
	***Assessment of*** ***evidence***	None - no national coordinating body	National coordinating body but limited national data	National coordinating body reviews aggregated data with expert advice where needed and regional network	National coordinating body reviews aggregated data with expert advice where needed and regional network
	***Intervention***	None - no national coordinating body	National coordinating body but limited national data	Surveillance data drive national policy and international policy	Surveillance data drive national policy and international policy
**Training**	***Clinical***	None	None	Training programme "train the "trainers" in core clinical surveillance procedures	Established national and international standards and accreditations to deliver training programmes
	***Laboratory***	None	None	Training programme "train the "trainers" in core lab surveillance procedures	Established national and international standards and accreditations to deliver training programmes
	***Data***	None	None	Training programme "train the "trainers" in core data surveillance procedures	Established national and international standards and accreditations to deliver training programmes
**Quality** **Assurance**	***Clinical***	None	None	Annual audit of clinical surveillance at sites	Quarterly audit of clinical data submitted through automated systems and comparison with other sites
	***Site lab***	None	None	Annual audit of laboratory standards	QA assessment of laboratory site to international standards
	***Reference lab***	No reference laboratory in-country	National reference laboratory but no standard protocols for AMRS	National reference laboratory, does QA with repeat testing of a subset of isolates	National reference laboratory, does QA with repeat testing of a subset of isolates, can do molecular testing (e.g. PCR) and/or local or international collaboration in place for next generation sequencing of isolates
	***Data***	None	None	Annual audit of data systems at sites	Support for automated sharing of data from sites for national aggregation
**National** **reference lab**	***Storage of*** ***isolates***	No biorepository, or no documentation of stored samples.	Biorepository of isolates, paper- based database with storage of at least a subset of resistant isolates defined by local policy (-20 or -80C)	Biorepository of isolates, with paper or electronic database with storage of all resistant isolates (-20 or -80C) defined by national policy	Biorepository of isolates, with electronic database of all stored resistant isolates defined by national and/or international policy, maintained at -80C.
	***Transport to*** ***reference lab***	Isolates are not transferred to reference lab	Isolates are transferred to ref lab on an ad-hoc basis	Isolates are transferred to ref lab annually	Isolates are transferred to ref lab at least annually according to international biosafety standards

### Costing

In each sub-function, the interventions, activities and resources needed to move from one level to the next were defined. The costs of establishing and implementing (start-up costs), as well as running (recurrent costs) of the described activities were included and recorded following an ingredients approach, where unit costs and quantities were required to calculate total costs for each input. This allowed the cost estimates to be adapted more easily to different country settings, by applying local unit costs. The cost results were presented by year, ranging from year 1 to year 5. Due to the different capacities and models used in the different sites that were visited, as well as logistical constraints, costs were assessed in a single highly functioning site in Kenya (KEMRI-Wellcome Trust Research Programme), where clinical, laboratory and demographic surveillance systems are integrated. We used site specific costs, for personnel, for subsequent specific country estimates. Data collection for the costing involved interviews with key personnel, review of key documents and observation of a functioning clinical, laboratory and demographic surveillance system. The approach to the costing was pragmatic, prioritising the most accurate recording of high cost items (with most influence on estimates of total cost). All information was collected and analysed in Excel 2016.

## Results

### Desk based analysis

Capacity for AMR surveillance varies between sub-Saharan Africa and South-east Asia, and within countries in these regions. In the WHO African region (AFRO), a recent review reported only 2 of the 47 AFRO member states (Ethiopia and South Africa) had national AMR plans in place ^5, 10^, and 7/47 members (Ethiopia, Ghana, Kenya, Lesotho, South Africa, Tanzania and Zimbabwe) had overarching national infection prevention and control (IPC) policies ^11^. Establishment of AMR surveillance is limited, but capacity is being developed, for example in 24 hospital laboratories in Ghana ^12^. In contrast, South-East Asian countries have, are developing, or are initiating AMR surveillance systems, for example, Thailand, Vietnam, and Cambodia ^13^, respectively. This is in part supported by the more advanced development of networks and collaborations for AMR surveillance. Networks and examples of AMR surveillance and stewardship programmes are described in detail in Supplementary File 1, as well as surveillance of other infectious diseases. AMR surveillance strategies could also learn from and benefit from recent laboratory strengthening efforts as an important element of the work against the Ebola epidemic in West Africa since 2014 ( Supplementary File 1: Box 3) and for specific diseases, for example rotavirus ( Supplementary File 1: Box 4) ^14^.

Emerging themes in strengthening laboratory capacity for AMR surveillance included:

*1. Leadership*

Leadership, and the national policy addressing AMR, as well as international collaboration, are necessary to strengthen AMR surveillance in LMIC countries, to ensure an enabling policy environment. However expertise in microbiology is limited in these settings, and, whilst in the longer term this capacity is important to develop, in the interim leaders may need to be drawn from clinical infectious diseases and public health. Leaders are important for advocacy/championing, and for local credibility of the surveillance effort, as well as providing the focal point for activities.

*2. Training*

Where health systems are weak and still developing, there is a need to develop human resources across cadres of staff: clinical, laboratory and data management. Well-trained staff are likely to move elsewhere for better salaries; it is important that there is sufficient budget to ensure staff can be recruited and retained.

*3. Laboratory quality assurance*

In terms of strengthening laboratory capacity, it is clear that inadequate laboratory infrastructure limits the quality and the ability to reliably detect pathogens and conduct antimicrobial susceptibility testing.

There are different options for models of AMR surveillance (outlined in Supplementary File 1 and summarised in Supplementary File 1: Table 1). Overall, a model with active, continuous, comprehensive integrated population and laboratory disease surveillance would provide the most robust, comprehensive data. However, within the constraints of resources, an appropriately designed sentinel site system would also be appropriate. Whilst it would be feasible to assess AMR outside of a health care context, for example through cross-sectional studies of population colonisation (as part of a population based survey), these would not include surveillance of the most serious drug resistant infections, and fall outside the remit of public health surveillance as observational research studies requiring appropriate ethical approvals and individual informed consent.

Laboratory based surveillance identifies “a resistant organism”, but without clinical or population data cannot be fully interpreted and inform policy. To achieve the aim of AMR surveillance, and inform interventions, data are needed on:

• the proportion of the particular bacterial species identified (e.g. *Klebsiella pneumoniae*) that are resistant
• whether resistance is developing in a particular patient group (e.g. neonatal), or particular presentations (e.g. pneumonia)
• whether this is localised to a setting (e.g. ward, or geographic area)
• who seeks health care
• who lives in the population catchment area

Within the different models of surveillance, the functions are broadly consistent, and can be split into those provided at a national level and those provided at one or more sites, depending on capacity and the model in place, and including the themes identified above. At a national level, there is a need for leadership to support training, and quality across the system. A national AMR laboratory can support development of site laboratories and conduct more extensive testing (thus focussing resources, Supplementary File 1: Table 3, Figure 1). At a site level an integrated model includes development of laboratory capacity (isolate identification and susceptibility testing), clinical surveillance (systematic investigation of patients according to diagnosis), health care utilisation surveys (to assess representativeness of the health facility data) and census or enumeration data to determine the population catchment ( Supplementary File 1: Table 2, Figure 1). These functions were used to cost AMR surveillance ( Supplementary File 1: Tables 2 and 3) and consider how they were being achieved in the different models and contexts of the country case studies.

### Country case studies

Full site reports are included in Supplementary File 1, the findings for each country are summarised here.

***Malawi.*** Malawi faces substantial challenges due to extremely limited country resources, which reduces health system capacity. However, whilst it does not have the capacity for a comprehensive AMR surveillance system, it has been developing institutional infrastructure and policy for AMR surveillance. It also has considerable capacity through international collaboration and an academic centre of excellence (Malawi-Liverpool Wellcome Trust (MLW)) undertaking microbiological surveillance as part of a research programme. In order to take advantage of available research infrastructure i.e. sentinel surveillance capacity beyond Queen Elizabeth Central Hospital (QECH), the following should be considered for new surveillance sites in Malawi:

1. *Whether current capacity is representative of its area*
  The current AMR surveillance is conducted at the referral hospital (QECH), which limits the representativeness of the sampled patient population in relation to Blantyre district as a whole. It is therefore important to consider supporting surveillance capacity development in other sites, using, if possible, the expertise available in country.
2. *What the needs for investment/enhancement are in this setting*
  Clinical surveillance and sampling is being strengthened with triage nurses to support systematic investigation in QECH. Microbiological surveillance is very strong, but electronic linking of clinical and microbiological databases is needed at this site and extension sites.
3. *Whether the hospital is connected to national institutions, and regional networks*
  There is growing collaboration between MLW, QECH and the Ministry of Health in Lilongwe, which would be needed to support extension of AMR surveillance sites.

***Ethiopia.*** Ethiopia is developing a system for AMR surveillance that includes a national reference laboratory and sentinel regional laboratories across the country reporting data (continuous, passive surveillance). At present, whilst capacity is in development, there are some hospitals where the standard of clinical examination is adequate for surveillance, but there is limited microbiology laboratory capacity for processing specimens for culture, particularly blood cultures. Record keeping is not formalized or electronic, and there is potential for improved communication between the ward and the laboratory to link microbiology data to patients. In strengthening capacity for AMR surveillance, the following should be considered:

1. *Determine whether the hospital(s) are epidemiologically representative*
  It is important to include a range of health care settings for AMR surveillance; district hospitals, outside of the capital are more generalizable to the Ethiopian population as a whole than very urban settings.
2. *Establish surveillance within the hospital*
  Systems for development would be national guidelines supporting standardised clinical examination and systematic investigation. Laboratory capacity needs strengthening in terms of automated blood cultures to support AMR surveillance to a core level. Clinical and laboratory data should be linked, this could be through a double sided form with data collection for both.
3. *Connect the site to a national and regional network*
  The Ethiopian Public Health Institute is an arm of the Federal Ministry of Health and combines public health policy making with laboratory capacity in the same institution. Stronger regional links could support capacity development as well as continued partnership with international organizations.

***Vietnam.*** Vietnam has established national infrastructure for AMR and has worked with regional networks and international collaborators to develop a surveillance system, including a national reference laboratory and twenty laboratories attached to district hospitals. The hospitals are public and broadly representative, there are functioning microbiology laboratories and the physical, legal and ethical capacity to link these records for anonymised aggregation at individual case level. In strengthening capacity for AMR surveillance, the following should be considered:

1. *Consider the hospitals in the network*
  The hospitals in the network are mid points in the hierarchical system in Vietnam and broadly representative. However, there would be considerable benefit from understanding health care utilisation patterns and population catchments to support laboratory data interpretation. In addition, given the ready accessibility and high volume use of antibiotics in Vietnam, surveillance may be needed at lower levels of health care.
2. *Establish surveillance within hospital*
  It is important that clinical examination and investigations are strengthened in the AMR surveillance system, to have the benefits of an integrated model, as well as the strengthening of laboratory microbiology services.
3. *Build a network within country (and on to WHO)*
  Many of this activities are in place in Vietnam, supported by strong national leadership and collaboration, and a hierarchical public health care system where national strategies can be implemented into care.

### Costing

We developed a flexible model, based on an assumption that each country would have a number of sentinel sites with clinical and laboratory surveillance taking place in each site. We made an assumption that a country would aim for one surveillance facility per five million population, with a minimum of five surveillance facilities per country. However, cost results are presented for varying number of sites per country (2, 5 and 10), rather than for the target number of sites, with a focus on costing inputs required to achieve surveillance at level 3 (a core level) and level 4 (an extended level).

For a sentinel site ( Table 1), each sub-function was broken down into all input activities and resources required to achieve the different levels of surveillance sophistication (core and extended) and summarized under “interventions” below each sub-function in clinical surveillance, laboratory surveillance, isolate storage and transport, data system and review ( Supplementary File 1: Tables 4A–D). A number of inputs were costed independently of the sub-function framework, for example personnel costs required for laboratory surveillance or the cost of setting-up a laboratory including the cost of building and equipment. They are presented directly as inputs. The additional demographic component to surveillance was costed as an initial and final population enumeration round in year 1 and 5. Inputs of activities and resources required at the national surveillance level are shown in Table 2. The inputs ( Supplementary File 1: Tables 4A–D and 5) were used to cost the surveillance components by determining quantities and unit costs at different levels of surveillance sophistication.

Costs depend on existing capacity, number of sentinel sites proposed for surveillance, and, across countries, variability in costs of personnel. Illustrations are given for Kenya and Vietnam according to different numbers of sites ( Supplementary File 1: Figures 7 and 8) across clinical, laboratory, national and demographic surveillance, as well as data system & review functions.

The largest contributor to the total cost of AMR surveillance is laboratory surveillance at the sentinel site (including isolate storage and transport to national reference laboratory), together with the national reference laboratory cost. These laboratory costs range, depending on number of sites and country, between 67% and 77% of the total cost at level 3 and between 78% and 85% at level 4. Within the laboratory cost the largest contributors to the total costs are the initial set up cost of the laboratory, and personnel. The increase in cost from core laboratory functions to extended laboratory functions is substantial ( Supplementary File 1: Figures 6 and 7) and is driven by the increase in equipment and staff to support the increase in samples being processed (all sample types vs blood cultures), and isolates tested (all isolates vs WHO priority pathogens (excluding *Neisseria gonorrhoeae,* omitted as frequently tested for in outpatient settings with swabs)).

Within the overall envelope costed for AMR surveillance, the costs for clinical surveillance, data system and demographic surveillance are a much smaller proportion of total cost than laboratory surveillance. This is partly because for the clinical elements the costs of clinical staff providing care were not included, as in-post, except where additional clinical staff would be needed to undertake specific activities needed to support surveillance. In contrast, laboratory personnel costs were all included in the costing. However, this likely reflects the real-world situation, in terms of the need for recruitment of laboratory staff.

## Conclusions

We aimed to map and compare existing models for laboratory system strengthening for AMR surveillance in LMICs. The work was based on desk review and three case studies on Ethiopia, Malawi and Vietnam, as well as costing based on a site visit at KEMRI Wellcome Trust Research Programme, Kilifi, Kenya. In the settings described in sub-Saharan Africa and South-east Asia, the AMR surveillance models are continuous, largely passive, laboratory based, using sentinel sites in country. An integrated model would be preferable for AMR surveillance so that data can be interpreted in a known clinical and demographic context, and case study countries were examined with this in mind.

It was apparent that the three countries visited were very different in terms of institutional architecture, health systems and AMR surveillance capacity, even on the short site visits undertaken for this work. For example, in Ethiopia there was early development of laboratory capacity in some broadly representative public hospitals, but no linked clinical data system, or population data; in Malawi there was very limited laboratory capacity in public hospitals, but a highly functioning research centre conducting surveillance in Blantyre, with high quality laboratory data, basic clinical data and a population denominator for urban and rural Blantyre; in Vietnam there was a broadly functioning laboratory capacity with a network of representative public hospitals across the country, supported by a consortium of international collaborators and with major investment but conducted at a level of the referral hierarchy (District hospitals), which is strongly susceptible to biases attributable to antibiotic access lower down in the referral hierarchy.

There were factors that were consistent in supporting and driving success in AMR capacity ( Supplementary File 1: Figure 9). These include political commitment and collaboration amongst and between government and international stakeholders to utilise local and international expertise in supporting AMR surveillance development, as well as national policies on AMR surveillance developed within legal and ethical frameworks. Although an assessment of interventions in relation to AMR was beyond the scope of this work, political commitment and collaboration amongst stakeholders will also be crucial when considering and implementing these interventions (e.g. stewardship and antibiotic use), and initiating policy change.

It was clear that a functioning health system can support development of an integrated sentinel surveillance site system, progressing perhaps to comprehensive systems, more readily than a weaker health system. The integration of AMR surveillance into a health system requires an assessment of the form, state and functioning of a country’s health system. This is a central issue as the degree to which AMR surveillance will benefit national and local concerns (as opposed creating further burdens to staff) will largely be determined by the strength of its country’s health systems.

An important component, and common limitation of health systems in LMICs are the human resources to do work, across all cadres of staff, including clinical, laboratory, managerial, policy-making, data analysis, project management groups. Microbiological expertise is particularly limited. There were examples of support for human capacity development in the country case studies through Universities (national and international), research institutions (national and international) and interest in developing technical postgraduate programmes. However, where the health systems need strengthening there is insufficient training available in country to support the development of technical capacity to the level required. In these situations, AMR surveillance will be more likely to succeed if it is built up more slowly, using the expertise available from the most appropriate institutions. To ensure success at a “core level”, strengthening of surveillance may require a staged process with capacity built up over time. Here one site would be supported (which could be through a national reference laboratory, external partner, or research laboratory) to build capacity. An example of this would be utilising the expertise available in the Malawi Liverpool - Wellcome Trust Research Programme to support AMR surveillance in a district hospital. With development of capacity to a core level at a sentinel surveillance site, this site could subsequently serve as a training and support centre to develop capacity at another site, thus overcoming barriers to high quality surveillance when capacity is thinly spread across a weak health system. From this point, over time, a network of sites could be developed, as they are being developed in Vietnam, with the potential for sub-regional reference laboratories as the network expands.

Laboratory infrastructure with the capacity for standardised, quality diagnostics, are important. However, they have to be supported by appropriate logistical support (procurement, budget management), otherwise even the most highly equipped laboratories will not be able to function. With this is mind, securing a core set of standard activities, which can be maintained and result in useful, interpretable data is more important in the early stages of capacity development than the use of more advanced techniques. It also requires considerably less investment, and supporting five sentinel sites to a core level will likely produce more generalizable data than two sites, who have an extended capacity, for a similar investment.

Countries who have fragile and/or transitioning health systems are least able to respond to the present burden of infectious disease. Surveillance capacity depends on the existing health system and considerable investment is needed to develop laboratory infrastructure aligned to this. High level expertise in-country to provide advocacy, championing and trusted guidance to the Ministry of Health is important. Engagement with both the Ministry of Health and other national stakeholders is essential, combined with the expertise of in-country partners such as the public health organizations or academic institutions. Establishing whether these infections are treatable will inform treatment and facilitate a response; contributing to reducing the inequities in understanding disease burden worldwide.

## Data availability

The data referenced by this article are under copyright with the following copyright statement: Copyright: © 2017 Seale AC et al.

A full report on the data collected are included as Supplementary File 1. A costings tool in Excel 2016 can be made available through the corresponding author on request. This allows costs of AMR surveillance to be estimated based on the target number of sites in a country.

## Supplementary material

Supplementary File 1: A fully formatted report with all Tables and Figures entitled “Supporting surveillance capacity for antimicrobial resistance: Laboratory Capacity Strengthening for Drug Resistant Infections in Low and Middle Income Countries”.
