# Supplementary material for: Supporting surveillance capacity for antimicrobial resistance: Laboratory capacity strengthening for drug resistant infections in low and middle income countries
Source: Wellcome Open Res. 2017 Sep 26;2:91. [Version 1] doi: 10.12688/wellcomeopenres.12523.1 (PMC5686477; doi:10.12688/wellcomeopenres.12523.1)
Supplement: Supplementary file 1 [file wellcomeopenres-2-13561-s0000.tgz › 5cc2ca53-6843-4d7a-ade4-92a40d484b4d.pdf]

Funded by UK Government

2016

LONDON SCHOOL of HYGIENE & TROPICAL MEDICINE

Supporting surveillance capacity for antimicrobial resistance

LABORATORY CAPACITY STRENGTHENING FOR DRUG RESISTANT INFECTIONS IN LOW AND MIDDLE INCOME COUNTRIES

Contents

Abbreviations 3
Executive Summary 5
1. Introduction 6
1.1 Overview 6
1.2 Objectives 6
2. Methodology 7
2.2 Desk based analysis and conceptual model 7
2.3 Case studies 7
2.4 Analytic framework 8
2.5 Costing 8
3. Desk-based analysis and conceptual framework 9
3.1 Aims of AMR surveillance 9
3.2 Overview of current capacity for AMR Surveillance in Sub-Saharan Africa and South-east Asia 9
3.3 Developing capacity for AMR surveillance 13
Leadership 13
Training 13
Laboratory quality assurance 13
3.4 Surveillance options 13
Active vs Passive surveillance 13
Episodic vs Continuous 14
Sentinel vs Comprehensive 14
Population based survey vs Laboratory based surveillance vs integrated disease surveillance 14
3.5 Surveillance functions 14
4. Case study: Malawi 22
4.1 Context 22
4.2 Leadership 23
4.3 Training 23
4.4 Quality assurance 23
4.5 Surveillance functions 24
Laboratory infrastructure and logistics 24
Clinical surveillance capacity 25
Population: health care utilisation and catchment 26
4.6 Summary 26
5. Case study: Ethiopia 28
5.1 Context 28
5.2 Leadership 29
5.3 Training 30
5.4 Quality Assurance 30
5.5 Surveillance functions 32
Laboratory infrastructure and logistics 32
Clinical surveillance capacity 34
Population: health care utilisation and catchment 34
5.6 Summary 34
6. Case study: Vietnam 36
6.1 Context 36
6.2 Leadership 37
6.3 Training 38
6.4 Quality assurance 39
6.5 Surveillance functions 39
Laboratory infrastructure and logistics 39
Clinical surveillance 40
Population catchment 40
6.6 Summary 41
7. Costing AMR surveillance 42
7.1 Scope 42
7.2 Description of inputs for each function 42
7.3 Costing results 58
8. Conclusions 62
9. References 64
10. Acknowledgements and attributions 69
11. Appendices 70
A: Summary features of pilot surveillance study in three geographic regions in Ghana 70
B: Summary of AMR networks and governance in South Africa[85] 72
C: Laboratories assessed as part of SURVAC 74
D: Findings from SURVAC (Surveillance Epidémiologique en Afrique Centrale) program for rotavirus gastroenteritis surveillance in Central Africa 75
E: Current status of AMR surveillance efforts in the Philippines 77
F: Summary of initiatives taken to contain antibiotic resistance in Cambodia 80
G: Current status of AMR surveillance efforts in Thailand 81

Abbreviations

**AFENET**	African Field Epidemiology Network
**AFRO**	African Regional Office (of WHO)
**AHRI**	Armauer Hansen Research Institute
**AMR**	antimicrobial resistance
**AMRS**	antimicrobial resistance surveillance
**ANSORP**	Asian Network for Surveillance of Resistant Pathogens
**APEC**	Asia Pacific Economic Organisation
**APFID**	Asia Pacific Foundation for Infectious Diseases
**APUA**	Alliance for Prudent Use of Antibiotics
**AST**	antimicrobial susceptibility testing
**ASU**	Antibiotics Smart Use
**CAESAR**	Central Asian and Eastern European Surveillance of Antimicrobial Resistance
**CARB**	Combating Antimicrobial Resistance Bacteria
**CDC**	US Centers for Disease Control and Prevention
**CLSI**	Clinical and Laboratory Standards Institute
**CSF**	Cerebrospinal fluid
**DACA**	Drug Administration and Control Authority (of Ethiopia)
**DFID**	Department for International Development
**DH**	Department of Health
**DHIS**	District Health Information System
**DRI**	Drug Resistant Infection
**FMHACA**	Ethiopian Food, Medicine and Healthcare Administration and Control Authority
**EPHI**	Ethiopian Public Health Institute
**EVD**	Ebola Virus Disease
**FAO**	Food and Agriculture Organization
**EARS-Net**	European Antimicrobial Resistance Surveillance Network
**EUCAST**	European Committee on Antimicrobial Susceptibility Testing
**FELTP**	Field Epidemiology and Laboratory Training Programs
**FETP**	Field Epidemiology Training Programs
**FSA**	Food Security Agency
**EHR**	Electronic Health Records
**EQA**	External Quality Assurance
**GAP**	Global Action Plan
**GARV**	Group A Rotavirus
**GDP**	Gross Domestic Product
**GHS**	Global Health Security
**GLASS**	Global Antimicrobial Resistance Surveillance System
**GSA**	Global Security Agenda
**HMIS**	Health Management Information System
**ICEMR**	International Center of Excellence for Malaria Research
**IDSR**	Integrated Disease Surveillance and Response
**IHME**	Institute of Health Metrics Evaluation
**IQC**	Internal quality control

**JICA**	Japan International Cooperation Agency
**KEMRI**	Kenyan Medical Research Institute
**LCC**	Local coordinating centre
**LMIC**	Low- and Middle-Income Countries
**LSHTM**	London School of Hygiene and Tropical Medicine
**MALDI-TOF**	Matrix-assisted laser desorption/ionization time of flight
**MDR-TB**	Multi-drug resistant tuberculosis
**MLW**	Malawi-Liverpool Wellcome Trust
**MOU**	Memorandum of Understanding
**NAP**	National Action Plan
**NARST**	National AMR Surveillance Centre Thailand
**NCC**	National Coordinating Centre
**NDoH**	National Department of Health
**NGO**	Non-governmental organisation
**NRL**	National Reference Laboratory
**NTP**	National Training Programme
**OUCRU**	Oxford University Clinical Research Unit
**PFSA**	Pharmaceutical Fund and Supply Agency
**PMC**	Preventative Medicine Centers
**PPE**	Personal protective equipment
**QECH**	Queen Elizabeth Central Hospital
**ReLAVRA**	Latin American Antimicrobial Resistance Surveillance Network
**RRL**	Regional Reference Laboratory
**SARS**	Severe Acute Respiratory Syndrome
**SASCM**	South African Society for Clinical Microbiology
**STI**	Sexually Transmitted Infection
**TAG**	Technical Advisory Group
**UKNEQAS**	United Kingdom National External Quality Assurance Scheme
**USAID**	United States Agency for International Development
**VFA**	Vietnam Food Administration
**VINARES**	Vietnam Resistance Survey
**VITMES**	Vietnam TB Information Management Electronic System
**VPD**	Vaccine Preventable Disease
**WHO**	World Health Organization
**WHONET**	World Health Organization laboratory database software

Executive Summary

Development of antimicrobial resistance (AMR) threatens our ability to treat common and life-threatening infections. Identifying the emergence of AMR requires strengthening of surveillance for AMR, particularly in low and middle-income countries (LMICs) where the burden of infection is highest and health systems are least able to respond. This work aimed, through a combination of desk-based investigation, discussion with colleagues worldwide, and visits to three contrasting countries (Ethiopia, Malawi and Vietnam), to map and compare existing models and surveillance systems for AMR, to examine what worked and what did not work.

Current capacity for AMR surveillance varies considerably in LMICs, but where systems are being developed they are focussed on laboratory surveillance. This approach limits our understanding of the epidemiology of AMR and the extent to which laboratory results can inform local, national and international public health policy. An integrated model, combining clinical, laboratory and demographic surveillance in sentinel sites is more informative and the costs for clinical and demographic surveillance are proportionally much lower than laboratory costs across different countries and numbers of sentinel sites.

The speed and extent to which AMR surveillance can be strengthened depends on the functioning of the health system, and the resources available. Where there is existing laboratory capacity, it may be possible to develop 5-20 sentinel sites with a long term view of establishing comprehensive surveillance; but where health systems are weaker and laboratory infrastructure less developed, available expertise and resources may limit this to 1-2 sentinel sites. Prioritising core functions, such as automated blood cultures reduces the investment needed at each site. Expertise to support AMR surveillance in LMICs may come from a variety of international, or national, institutions, working on research or policy. It is important that these organisations collaborate to support the health systems on which AMR surveillance is built, as well as improving technical capacity specifically relating to AMR surveillance. Strong collaborations, and leadership, drive successful AMR surveillance systems across countries and contexts.

1

Introduction

1.1 Overview

Since the introduction of antibiotics into medicine in the 1940s, they have provided **dependable treatment** for many infectious diseases. The emergence of antimicrobial resistance (AMR) and related drug resistant infections (DRIs) challenges this.[1] One of the early priorities identified to tackle the emergence of AMR and DRIs is to strengthen surveillance of AMR in low and middle income countries (LMICs).[2]

Despite LMICs having the highest incidence of infection (and associated mortality and morbidity) there are significant gaps in our understanding of the aetiology and incidence of infectious diseases in these contexts.[3,4] This constrains national governments and international organisations in their efforts to detect evolving trends and emerging threats.

Data on the true prevalence of AMR in LMICs are limited. In 2014, the World Health Organization (WHO) summarized the most recent information on AMR surveillance for a selected set of nine bacteria–antibacterial drug combinations of public health importance from 129 Member States.[5] Among WHO regions, the greatest volume of country-level data were obtained from the European Region and the Region of the Americas, where longstanding regional surveillance and collaboration exist. In contrast, **data from LMIC were underrepresented, and in some countries, lacking**. However, there is no evidence to suggest that the burden of AMR for many common pathogens in LMICs is not increasing in line with the rest of the world,[5] and can be the place of emergence.[6] Understanding current capacity for AMR surveillance in LMICs is important, as well as considering how to develop capacity in different settings and contexts, and their requisite costs.

Modelled estimates put the number of AMR attributable deaths per year in Europe at 25,000 with €1.5 billion in associated health care costs and productivity losses;[7] in the United States there are 23,000 deaths per year attributable to AMR at a direct cost of US$20 billion and an indirect cost of US$35 billion in productivity losses.[8,9] There are no reliable estimates available on the economic losses due to AMR available from LMICs, but they are likely to be considerable.

Despite the economic losses identified in high income countries, funding to tackle AMR has, until recently, been limited. This is in part because costs associated with establishing and running AMR surveillance were considered to be high.

1.2 Objectives

With a view to informing future capacity building for AMR surveillance in LMIC settings, this work aimed to characterise the requirements for achieving capacity to detect the emergence of AMR at national levels. The specific objectives were

i) To map and compare existing models for laboratory system strengthening (for AMR surveillance), including:

- The identification of specific contexts and geographical settings where certain models have been more or less successful
- An analysis of challenges and barriers in different geographical and sociodemographic contexts
- A consideration of costs and sustainability
- An assessment of governance issues, including the ability to share data

ii) To map and compare existing AMR surveillance systems in at least three countries, identifying:

- Different approaches for monitoring emergence and spread of resistance in different countries or regional settings, including the range of baseline data gathered
- The best models and mechanisms for surveillance, capacity strengthening and training in the different country or regional settings

Methodology

2

2.2 Desk based analysis and conceptual model

A team of experts in health systems, health economics, anthropology, microbiology, laboratory capacity, surveillance, and epidemiology was brought together. This group discussed and developed an initial outline of a **conceptual model for AMR surveillance** on which to frame the desk based analysis, case studies and costing of AMR.

A desk-based analysis of capacity for AMR surveillance in Sub-Saharan Africa and South-east Asia was done, first by reviewing the World Health Organization's (WHO) AMR report and then looking at **those countries that had been able to contribute data**, and identifying which components of the conceptual model they had put in place (or other) to do this. The challenges faced in these systems were also considered. Where there were few or no data in WHO sub-regions, examples of other surveillance systems that had been established, outside of the context of AMR surveillance were reviewed.

2.3 Case studies

Site visits were made to Malawi, Ethiopia and Vietnam, and capacity in these countries reviewed according to the **components included in the conceptual model and the functions that would be needed** to improve/develop AMR surveillance, as well as the **strengths of the systems and models** in place. This work involved extensive review of relevant guidelines and health system and policy literature, as well as discussions with in-country personnel, including laboratory staff, policy-makers, clinicians, microbiologists, veterinarians and epidemiologists.

2.4 Analytic framework

We identified the **functions, sub-functions and activities** needed for AMR surveillance **at a national and site level**, and defined four levels of AMR surveillance capacity involving increasing complexity.

Capacity at Level 1 represents the most limited system, with incomplete clinical data, minimal clinical investigation and no laboratory testing. At level 2 there is some laboratory testing but no standardization of processes in place. Level 3 represents a **core surveillance system** with standardized processes, focusing on **invasive disease in children under five years** and the identification and **antimicrobial susceptibility testing of WHO priority pathogens** (*Escherichia coli, Klebsiella pneumoniae, Staphylococcus aureus, Streptococcus pneumoniae, Acinetobacter baumannii, Salmonella* spp., *Shigella* spp., *Neisseria gonorrhoeae*). Level 4 represents **extended** AMR surveillance capacity, with capacity to include AMR surveillance from all patients and all sample types.

The four functions at the sentinel site level were 1) clinical admission assessment & investigation; 2) isolate identification and susceptibility testing; 3) isolate storage (local) & referral to reference laboratory and 4) data system and review. At the national level these functions were 1) leadership; 2) training; 3) quality assurance and 4) national reference laboratory.

Each function was further broken down into sub-functions; for example "clinical admission assessment & investigation" has four sub-functions, namely i) clinical admission assessment; ii) clinical data; iii) clinical investigation and iv) training and quality assurance, all describing the different activities relevant for clinical surveillance.

2.5 Costing

In each sub-function the interventions, activities and resources needed to move from one level to the next are defined. The costs of **establishing and implementing** (start-up costs), as well as **running** (recurrent costs) of the described activities were included and recorded following an **ingredients approach**, where unit costs and quantities are required to calculate total costs for each input. This allows the cost estimates to be adapted more easily to different country settings, by applying local unit costs. The cost results were presented by year, ranging from year 1 to year 5.

Due to the different capacities and models used in the different sites that were visited, as well as logistical constraints, **costs were assessed in a single highly functioning site** in Kenya (KEMRI-Wellcome Trust Research Programme), where clinical, laboratory and demographic surveillance systems are integrated. We used site specific costs, for personnel, for subsequent specific country estimates.

Data collection for the costing involved interviews with key personnel, review of key documents and observation of a functioning clinical, laboratory and demographic surveillance system. Interviewees included clinicians, nurses, the surveillance officer, laboratory manager, procurement manager, microbiologist, lab technicians, finance officer, human resources manager, head of information technology (IT), IT project manager, software developer, data manager, training and demographic surveillance leads. The approach to the costing was pragmatic, prioritising the most accurate recording of high cost items (with most influence on estimates of total cost). All information was collected and analysed in Excel.

Desk-based analysis and conceptual framework 3

3.1 Aims of AMR surveillance

The aim of AMR surveillance (box 1) is to provide data that informs local, national and international policy and interventions to control AMR and reduce Drug Resistant Infections (DRIs). The World Health Organization Global Action Plan sets out suggested **infrastructure for AMR surveillance**, as one component of a **national action plan.**[10]

Box 1: Defining AMR surveillance

Public health surveillance is "the on-going, systematic collection, analysis, and interpretation of health-related data with the *a priori* purpose of preventing or controlling disease or injury, or of identifying unusual events of public health importance, followed by the dissemination and use of information for public health action"[11].

Antimicrobial resistance (AMR) surveillance is an application of public health surveillance, which may refer to the development of resistance in any microbe. This analysis considers capacity for the detection of AMR in bacteria only, which result in drug resistant (bacterial) infections (DRIs). Viral, fungal and parasite resistance problems were beyond the scope of this work.

3.2 Overview of current capacity for AMR Surveillance in Sub-Saharan Africa and South-east Asia

Capacity for AMR surveillance varies between sub-Saharan Africa and South-east Asia, and within countries in these regions. In the WHO African region (AFRO) a recent review reported only **2 of the 47 AFRO member states** (Ethiopia and South Africa) **had national AMR plans** in place and 7/47 members (Ethiopia, Ghana, Kenya, Lesotho, South Africa, Tanzania and Zimbabwe) had overarching national infection prevention and control (IPC) policies.[12] In contrast South-east Asian countries have, or are developing, AMR surveillance systems, for example, Thailand and Vietnam, respectively. This is in part supported by the **more advanced development of networks and collaborations** for AMR surveillance.

In 1996, the Asian Network for Surveillance of Resistant Pathogens (ANSORP) was founded and has collaborated internationally to report AMR in major bacterial pathogens in 113 hospitals in 65 cities. In addition, the Asia Pacific Foundation for Infectious Diseases (APFID)

supports efforts to reduce DRIs in the Asia-Pacific region; **communication** of scientific issues is undertaken by ISAAR (International Symposium on Antimicrobial Agents and Resistance); **international microbial collection** and biorepository information is supported through the Asian Bacterial Bank (http://grbio.org/institution/asian-bacterial-bank). These networks have facilitated **active collaboration with the World Health Organization, Asia Pacific Economic Cooperation (APEC), regional academic societies and health-care organizations in individual countries** to prepare **international strategies and action plans**.

There are examples in the literature where countries in sub-Saharan Africa have developed their own infrastructure. **Ghana** has established **an AMR working group to create a policy platform,** develop a **policy framework** and implementation plan and to help **raise awareness** on AMR. This group is chaired by the Director of Pharmacy at the Ministry of Health and incorporates **key stakeholders in healthcare, regulatory authorities, academia, research institutions, veterinary clinics, and civil society organisations**. It builds on the Ministry of Health's Essential Medicines List and National Drug Policy (2010) and AMR engagement and dissemination work, such as the **first ever African Conference on antibiotic use and resistance held in Ghana** (March, 2015). There has subsequently been **capacity development for AMR surveillance in 24 laboratories in Ghana**, including capacity to enable bacteriological culture using in-house standard operating protocols. Surveillance data were stored and analysed using WHONET program files (results summarised in Appendix A).[13]

South Africa, whilst not typical of many other countries in sub-Saharan Africa, has AMR surveillance programmes led by the National Antibiotic Surveillance Forum (NASF)/South African Society for Clinical Microbiology (SASCM) and the National Department of Health (NDoH) (Appendix B).

Box 2: WHO external quality assessment (EQA) programme of national public health laboratories in Africa

Since 2002, the Regional Office for Africa of the World Health Organization (WHO) has invited national public health laboratories and related facilities in Africa to **participate in an external quality assessment (EQA) programme**. Specimens and questionnaires associated with bacterial enteric diseases, bacterial meningitis, plague, tuberculosis and malaria are sent annually to test diagnostic proficiency. By 2009, 78 laboratories from 48 WHO member states participated in the scheme and surveys were returned for up to 93% of participating laboratories.[11]

For bacterial enteric diseases and meningitis components, bacterial identification was acceptable in 65% and 69% of cases, respectively, but serotyping and antibiotic susceptibility testing and reporting were frequently unacceptable. Of the antimicrobial susceptibility test results, 43% of laboratories were rated 'unacceptable' for enteric pathogens and 75% were rated 'unacceptable' for meningitis.

The findings of this EQA programme highlight deficiencies in **equipment and consumables** (appropriate reagents), the need for **trained staff** and **logistical support; quality control mechanisms** and adherence to **internationally recognized guidelines**. The **African Society for Laboratory Medicine (ASLM)** is working with African countries to develop career planning for laboratory personnel to support **recruitment and retention.**

In South-east Asia, there are some advanced AMR surveillance programmes. In **Thailand**, for example (Appendix H)**,** national AMR surveillance has been established since 2000 by the National AMR Surveillance Centre Thailand (NARST). In addition, **antimicrobial stewardship** is encouraged through a pilot program called Antibiotics Smart Use (ASU). This aims to promote behavioural changes with respect to drug use at the level of prescriber (doctor, nurse, and pharmacist) and patient/consumer. A local healthcare team plans and names their **own Antibiotic Smart Use project** (giving a sense of **ownership**) based on **local and/or provincial policy**. The local healthcare team can request support from the ASU program, in the form of materials, speakers and/or technical support.

There is also development of capacity for AMR in the **Philippines, bringing together relevant stakeholders and programmes in a One Health Approach** (Appendix F).

Cambodia has more limited data on AMR in comparison to Thailand, but **health systems** are only now recovering from many years of civil war. Laboratory capacity has been developed in several hospitals (government and non-governmental) across Cambodia, some supported by **international collaboration**. In 2011, the Cambodian Ministry of Health (MoH) organised the '**First National Workshop on Antibiotic Resistance'** in close **collaboration** with many **national and international stakeholders**[14] (see Appendix G). This brought together **policy-makers, clinicians, pharmacists, laboratory technicians** and other professionals dealing with the problems of bacterial infection and AMR across the country. Antibiotic resistance data from fledgling and experienced laboratories were presented, confirming high rates of resistance in key pathogens to most antibiotics currently available in Cambodia. **A five-point plan** was discussed, which included initiatives from government and non-governmental partners, focusing on **rational prescribing, clinical practice** and **guidelines, improved laboratory services, infection prevention, and enhanced education** at all levels.

Following the meeting, **dedicated workshops** were organised for laboratory technicians and for clinicians. For laboratory personnel, particular attention was given to **quality control and stock management**. Priority pathogens (e.g. *Staphylococcus aureus*, *Escherichia coli*, *Salmonella* Typhi) were covered, but attention was also given to **pathogens of particular relevance in the country**, such as *Burkholderia pseudomallei*, *Salmonella enterica* and *Streptococcus suis*. For clinicians, training focused on the **interpretation of bacterial culture results** and the **medical management of difficult-to-treat cases**. The need for **locally adapted guidelines and training** at all levels on AMR was recognised (details in Appendix G).

With limited data on AMR surveillance programmes in sub-Saharan Africa much can be learnt from **surveillance of other diseases**, whilst acknowledging that capacity for high quality laboratory surveillance is limited (see Box 2). There have, more recently been laboratory strengthening efforts as an important element of the work against the Ebola epidemic in West Africa since 2014 (see Box 3) and for specific diseases, for example rotavirus (see Box 4).[15]

Box 3: Lessons from Ebola virus disease outbreak; the role of field epidemiology training programme in the fight against the epidemic, Liberia, 2014 [16]

There have been challenges, particularly with human resources, to the roll out and implementation of the World Health Organization (WHO) Regional Office for Africa's Integrated Disease Surveillance and Response (IDSR) strategy. In response, the African Field Epidemiology Network (AFENET) was established between 2005 and 2006 as a network of Field Epidemiology Training Programs (FETPs) and Field Epidemiology and Laboratory Training Programs (FELTPs) to support public health programs through disease surveillance and outbreak response in Africa. With its emphasis on a One Health approach for solving public health issues, the program has trained physicians, veterinarians and laboratory scientists to work jointly on human, animal and environmental health issues, and is in use in 20 countries in sub-Saharan Africa.

Despite this, recent rapid spread of Ebola Virus Disease (EVD) in Liberia, Sierra Leone and Guinea, was partly attributed to weak health systems, and lack of skills and knowledge among health workers, in addition to challenges with post-conflict recovery. However, the emergency has resulted in the training of health workers on Ebola infection control and prevention, and helped to build capacity for future outbreaks of EVD.[16] The skills and experiences acquired by these health professionals were instrumental in the control of EVD in Liberia, in addition to health systems strengthening through capacity building of the existing health workers and integrating surveillance of other diseases in the EVD surveillance activities.

Box 4: SURVAC (Surveillance Epidémiologique en Afrique Centrale)

SURVAC (Surveillance Epidémiologique en Afrique Centrale) was a **surveillance demonstration project (2009-2014)** to strengthen disease surveillance for rotavirus gastroenteritis, paediatric bacterial meningitis and later enhanced surveillance for measles and rubella and other priority outbreak diseases.[15] This was done in Cameroon (CAE), The Democratic Republic of the Congo (DRC) and Central African Republic (CAR).

The three overarching goals of SURVAC were to:

1.) Strengthen surveillance, response and quality through training and infrastructure;

2.) Implement a quality surveillance and response program for vaccine preventable diseases/syndromes, including laboratory capacity, networks and data management systems and

3.) Strengthen communications infrastructure and advocacy to ensure sustainability through the respective Ministries of Health. To do this, the relevant Ministries of Health listed potential surveillance laboratories and a multi-institutional team assessed laboratory capacity and systems. There were five laboratories assessed in Cameroon, six in DRC, five in CAE (see appendix C).

SURVAC showed that with **human and financial resources** including **training and mentorship**, the implementation of surveillance using molecular technology was feasible in these countries. **Motivation** was high in this project, and **teamwork between epidemiologists and laboratory scientists was** key for success. Challenges for establishment of surveillance included **procurement** (shortage of reagents, delays in clearance at customs), **staff turnover** (especially once trained), **data management**, **theft of equipment and civil unrest** (appendix D). [15]

3.3 Developing capacity for AMR surveillance

Emerging themes in strengthening laboratory capacity for AMR surveillance identified include:

Leadership

Leadership, and the **national policy** addressing AMR, as well as **international collaboration**, are necessary to strengthen AMR surveillance in LMIC countries, and ensure an enabling policy environment. However **expertise in microbiology is limited** in these settings, and, whilst in the longer term this capacity is important to develop, in the interim leaders may need to be drawn from **clinical infectious diseases and public health**. Leaders are important for advocacy/championing, and for local credibility of the surveillance effort, as well as providing the focal point for activities.

Training

Where health systems are weak and still developing, there is a need to develop **human resources** across cadres of staff: **clinical, laboratory and data management.** Well-trained staff are likely to move elsewhere for better salaries; it is important that there is sufficient budget to ensure staff can be **recruited and retained**.

Laboratory quality

In terms of strengthening laboratory capacity, it is clear that **inadequate laboratory infrastructure limits the quality and the ability to reliably detect pathogens and conduct antimicrobial susceptibility testing (AST)**.

The **logistical challenges** relating to this, and the processes required to support laboratories are wide ranging, from **procurement**, in terms of equipment and consumables; to **servicing,** for example equipment should be bought with service contracts or with capacity to **train staff** to maintain equipment; to the need for appropriate **transport and storage** of laboratory equipment, consumables, specimens and isolates.

3.4 Surveillance options

There are different options for models of AMR surveillance, (summarised in Table 1) within which these themes would need to be addressed. Overall, **a model with active, continuous, comprehensive integrated population and laboratory disease surveillance** would provide the most robust, comprehensive data, as discussed below. However, within the constraints of resources, an appropriately designed sentinel site system would also be appropriate.

Active vs Passive surveillance

Passive surveillance models rely on reporting from sites; an **active** model would require a surveillance team to contact sites regularly for data. A passive model is less costly, and increases ownership at a surveillance site, but success of the surveillance system as a whole depends on complete and timely reporting from sites, making an active system preferable.

Episodic vs Continuous

Whilst in theory AMR surveillance **could be done on an episodic basis**, compared to continuous surveillance this **limits the data available**, and **decreases the ability to detect trends** in the causes of serious disease (assuming AMR surveillance on human health is set in a health care facility). Continuous surveillance also has the advantage that it is **linked to clinical gains**, you don't have to explain to clinical staff that you are withdrawing surveillance for a period of time. Surveillance becomes part and parcel of clinical care and is accepted as a clinical duty.

Sentinel vs Comprehensive

Comprehensive surveillance has the advantage that all health care providers are included in a system, which **limits bias** as the whole population is included. However, this is challenging where capacity is limited. **Sentinel systems require less capacity** as specific sites are selected, but efforts are needed to reduce bias and ensure these sites reflect the population.

Population based survey vs Laboratory based surveillance vs integrated disease surveillance

Whilst it would be feasible to assess AMR **outside of a health care context, for example through cross-sectional studies of population colonisation** (as part of a population based survey) these would **not include surveillance of the most serious DRIs**, and **fall outside the remit of public health surveillance** (Box 1) as observational research studies requiring appropriate ethical approvals and individual informed consent.

Laboratory based surveillance identifies "a resistant organism" but **without clinical or population data cannot be fully interpreted and inform policy**. To achieve the aim of AMR surveillance, and inform interventions, data are needed on:

- the proportion of the particular bacterial species identified (e.g. *Klebsiella pneumoniae*) that are resistant,
- whether resistance is developing in a particular patient group (e.g. neonatal), or particular presentations (e.g. pneumonia)
- whether this is localised to a setting (e.g. ward, or geographic area)
- who seeks health care
- who lives in the population catchment area

3.5 Surveillance functions

Within the different models of surveillance, the functions are broadly consistent, and can be split into those provided at a national level and those provided at one or more sentinel sites, depending on capacity and the model in place, and including the themes identified above.

1. **At a national level**, there is a need **for leadership** to support **training,** and **quality** across the system. A **national AMR laboratory** can support development of site laboratories and conduct more extensive testing (thus focussing resources, Table 2, Figure 1).

2. **At a site level** an integrated model includes development of **laboratory capacity** (isolate identification and susceptibility testing), **clinical surveillance** (systematic investigation of patients according to diagnosis**), health care utilisation surveys** (to assess representativeness of the health facility data) and census or enumeration data to determine the **population catchment** (Table 3, Figure 1).

These functions were thus used to cost AMR surveillance (Tables 2 and 3) and consider how they were being achieved in the different models and contexts of the country case studies.

Laboratory methods focussed on conventional approaches. Whilst molecular methods are attractive, and may become more so, they are not currently established for routine clinical and/or surveillance purposes in the context of the range of bacterial pathogens of interest for AMR. In addition, isolation of the organism (through conventional culture) permits further testing and detailed examination (for example whole genome sequencing) to establish phylogeny and the evolution of AMR.

Figure 1: Antimicrobial resistance surveillance model.

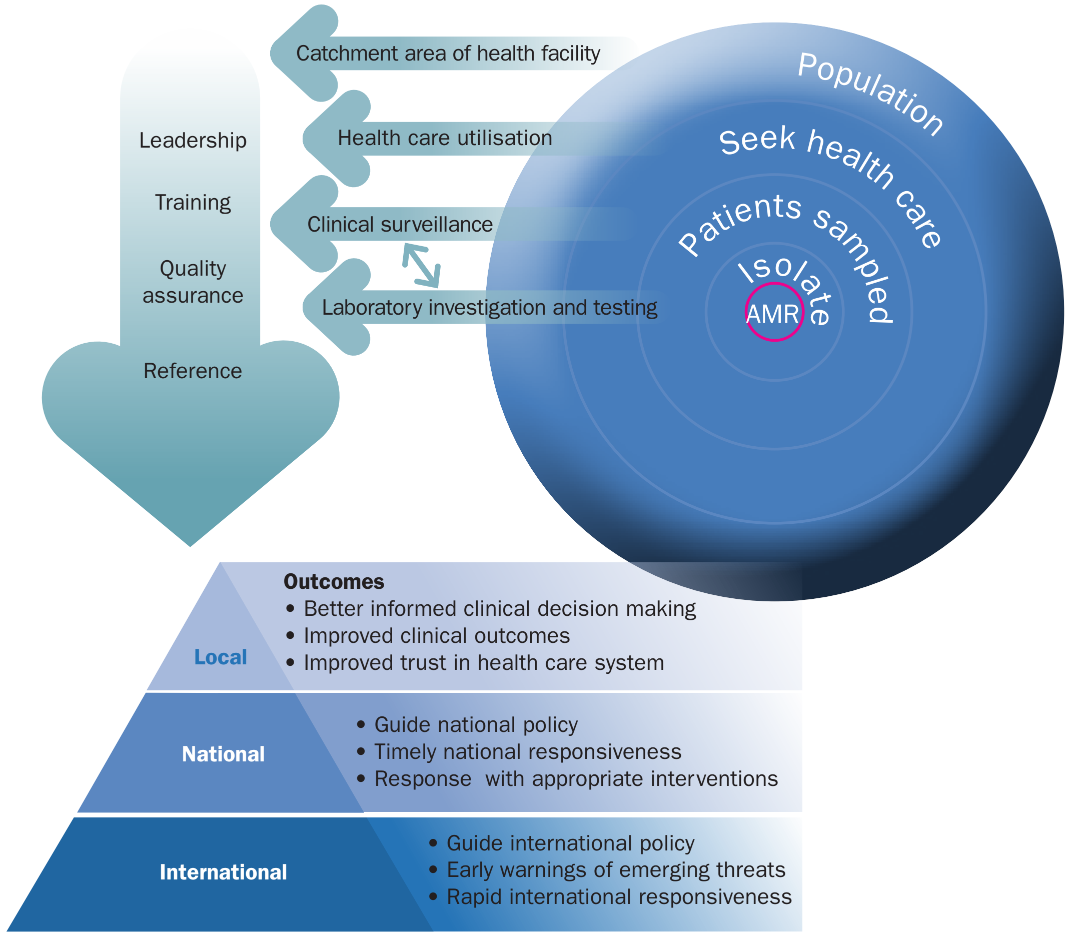

Table 1: Advantages and disadvantages of different types of surveillance

		Definition	Advantages	Disadvantages
Activity type	Active	Staff members contact heath care providers or the population to seek information about health conditions.	Increase response and thus AMR data quality (where available)	Cost in staff time; site ownership reduced
	Passive	Surveillance staff receive reports from health care facilities or others	Less cost in terms of staff time	Lower response and thus AMR data
Time course	Continuous	Ongoing surveillance	Increased data, clinical surveillance is integrated into care	Continued running costs; site fatigue
	Episodic	Surveillance for a limited period of time, repeated	Reduced running costs, decreased site fatigue	Decreased data, surveillance seen as an additional activity, outside of standard care
Number of sites	Comprehensive	Includes all health care providers/laboratories in the surveillance system to report all cases of a defined condition.	Increased data, representative of patients accessing health care system across the country	Cost of many sites, development and maintenance of capacity across these sites
	Sentinel surveillance	Requires an arranged sample of reporting sources to agree to report all cases of a defined condition, such that inferences can be made about the rest of the population.	Reduces costs, less capacity to be developed, easier to maintain quality at fewer sites	Decreased data with fewer sites, may not be representative if sites are not chosen strategically
System	Population based survey	Use standardised questionnaires, investigations and protocols to assess population levels of particular health conditions and/or other characteristics, e.g. Demographic and Health Surveys undertaken regularly in countries.	Less capacity needed if teams move across areas, less cost	Investigations not done for clinical care require individual consent, dunlikely to include AMR in serious illness cases
	Clinical surveillance	Syndromic surveillance; data on common clinical syndromes obtained from many settings and lab data could be modelled	Cost low, could be used alongside AMR data from laboratory surveillance	Limitations of models may limit usability of data
	Laboratory surveillance	Laboratory confirmed surveillance	AMR data from clinical settings	Laboratory capacity needed, AMR data not set in context of clinical and demographic data
	Integrated disease surveillance and response	Links epidemiologic and laboratory data in communicable disease surveillance systems across health facilities, and associated public health action.	AMR data set in context of clinical and demographic data to inform public health action	Cost likely higher than laboratory or clinical surveillance, or population based surveys

Table 2: Functions and sub-functions by level of surveillance sophistication at a site

Level of surveillance sophistication increasing from left to right					
		Level 1	Level 2	Level 3 - CORE	Level 4 - EXTENDED
Aim of AMR Surveillance		Limited	Surveillance data drive local policy	Surveillance data drive national policy and international policy	Surveillance data drive national policy and international policy
Functions	Subfunctions				
Clinical admission assessment and investigation	Clinical admission assessment	Clinical history and examination incomplete	Clinical history and examination, but no algorithms in use	Systematic clinical history and examination according to clinical algorithms in core patient group (< 5 years)	Systematic clinical history and examination according to clinical algorithms in all target patient groups
	Clinical data	Documentation incomplete	Paper based documentation or electronic system without linkage	Documentation into paper system using unique alphanumeric identifier which links to laboratory isolates	Standardised documentation into electronic system using unique alphanumeric identifier which links to laboratory isolates
	Clinical investigation	Clinical investigations ad-hoc	Clinical investigation based on clinical findings but not standardised	Standardised clinical investigation based on clinical findings (fever) in core patient group	Standardised clinical investigation based on clinical findings (fever) in all target patient groups
	Training and quality assurance	Not done	Ad hoc	Routine training to surveillance SOPs	Quality assurance of clinical data with external assessment
Isolate identification and susceptibility testing	Sample transport from clinical site to laboratory for processing	Samples not transported routinely	Samples transported, no standards in place to guide process	Samples transported according to local standard operating procedures	Samples transported according to international standards for biosafey
	Sample registration	No registration system	Paper based registration (logbook)	Local laboratory data system with manual linkage to clinical data on site	Local laboratory data system linked to clinical data with aggregation to national network (automated)
	Culture	No blood culture	No blood cultures	Automated blood culture, with processing the WHO 8 priority pathogens according to SOPs	Automated blood culture as well as csf, urine, stool and swab culture, processing all isolates according to SOPs
	Susceptibility testing	Not done	Done but not according to defined standards and/or without SOPs	According to SOPs for WHO 8 priority pathogens; qualitative testing	According to SOPs for all isolates
	Training and quality assurance	Not done	Ad hoc	Routine training to surveillance SOPs	Quality assurance of laboratory with external assessment; accrediation to ISO 15189 standards
Isolate storage (local) and referral to reference lab	Storage of isolates	No biorepository, or no documentation of stored samples.	Biorepository of isolates, paper-based database with storage of at least a subset of resistant isolates defined by local policy (-20 or -80C)	Biorepository of isolates, with paper or electronic database with storage of all resistant isolates (-20 or -80C) defined by national policy	Biorepository of isolates, with electronic database of all stored resistant isolates defined by national and/or international policy, maintained at -80C.
	Transport to reference lab	Isolates are not transferred to reference lab	Isolates are transferred to ref lab on an ad-hoc basis	Isolates are transferred to ref laboratory at least annually but not according to defined SOPs	Isolates are transferred to reference laboratory at least annually according to international standards for biosafety
	Training and quality assurance	Not done	Ad hoc	Routine training for isolate storage and transport SOPs	Routine training for isolate storage and transport SOPs;<br><br>Quality assurance of laboratory biorepository with external assessment
Data system and review	Data system	none	not done	Local laboratory data system with manual linkage to clinical data on site; system not linked to national network; paper based or electronic biorepository database that is not linked	Local laboratory data system electronically linked to clinical data and automatically linked to national network
	Data use	Limited	Data used for individual clinical care; hospital review for treatment guidelines	Submit to national network	Submit to international network (through country level surveillance)
	Data linkage	No linkage	No linkage	Manual linkage	Automated linkage
	Data governance	No data sharing policy	Local data sharing policy (in-hospital)	Data sharing policy and agreements in place in collaboration with the Ministry of Health and/or national public health institute (see national level)	Data sharing policy and agreements in place in collaboration with the Ministry of Health and/or national public health institute (see national level)

Table 3: Functions and sub-functions by level of surveillance sophistication at national level

Level of surveillance sophistication increasing from left to right					
		Level 1	Level 2	Level 3 - CORE	Level 4 - EXTENDED
Aim of AMR Surveillance		Limited	Surveillance data drive local policy	Surveillance data drive national policy and international policy	Surveillance data drive national policy and international policy
Functions	Subfunctions				
Leadership	Data analysis	None - no national coordinating body	National coordinating body but limited national data	National coordinating body reviews aggregated data with annual report	National coordinating body reviews aggregated data with annual report
	Data governance	None - no national coordinating body	National coordinating body but limited national data	National standards for data governance, legislation	National standards for data governance, legislation
	Assessment of evidence	None - no national coordinating body	National coordinating body but limited national data	National coordinating body reviews aggregated data with expert advice where needed and regional network	National coordinating body reviews aggregated data with expert advice where needed and regional network
	Intervention	None - no national coordinating body	National coordinating body but limited national data	Surveillance data drive national policy and international policy	Surveillance data drive national policy and international policy
Training	Clinical	None	None	Training programme “train the “trainers” in core clinical surveillance procedures	Established national and international standards and accreditations to deliver training programmes
	Laboratory	None	None	Training programme “train the “trainers” in core lab surveillance procedures	Established national and international standards and accreditations to deliver training programmes
	Data	None	None	Training programme “train the “trainers” in core data surveillance procedures	Established national and international standards and accreditations to deliver training programmes
Quality Assurance	Clinical	None	None	Annual audit of clinical surveillance at sites	Quarterly audit of clinical data submitted through automated systems and comparison with other sites
	Site lab	None	None	Annual audit of laboratory standards	QA assessment of laboratory site to international standards
	Reference lab	No reference laboratory in-country	National reference laboratory but no standard protocols for AMRS	National reference laboratory, does QA with repeat testing of a subset of isolates	National reference laboratory, does QA with repeat testing of a subset of isolates, can do molecular testing (eg PCR) and/or local or international collaboration in place for next generation sequencing of isolates
	Data	None	None	Annual audit of data systems at sites	Support for automated sharing of data from sites for national aggregation
National reference lab	Storage of isolates	No biorepository, or no documentation of stored samples.	Biorepository of isolates, paper-based database with storage of at least a subset of resistant isolates defined by local policy (-20 or -80C)	Biorepository of isolates, with paper or electronic database with storage of all resistant isolates (-20 or -80C) defined by national policy	Biorepository of isolates, with electronic database of all stored resistant isolates defined by national and/or international policy, maintained at -80C.
	Transport to reference lab	Isolates are not transferred to reference lab	Isolates are transferred to ref lab on an ad-hoc basis	Isolates are transferred to ref lab annually	Isolates are transferred to ref lab at least annually according to international biosafety standards

SUPPORTING SURVEILLANCE CAPACITY FOR ANTIMICROBIAL RESISTANCE

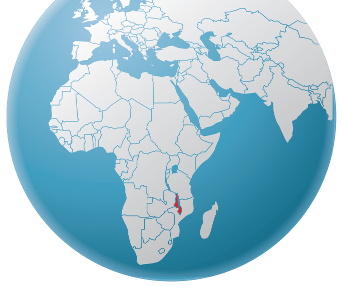

4 Case study: Malawi

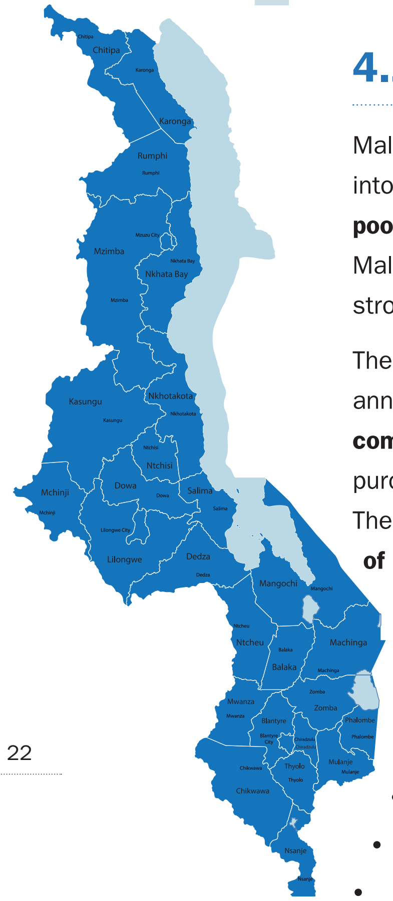

4.1 Context

Malawi has a population of over 16.1 million and a surface area of 118,500 km^2 , divided into three administrative regions and 28 districts (figure 2).[17] It is **amongst the world's poorest countries**, with a GDP/capita of $255.[18-20] Four of the five highest causes of death in Malawi are attributed to infectious diseases; HIV/AIDS, lower respiratory infections, malaria, stroke and diarrhoeal disease.[21]

The Malawian government is estimated to spend 9.6% of its GDP and 16.8% of its total annual expenditure on health.[19,23] However, **the majority of health system funding (74%) comes from international donor aid.** The government thus covers ~60% of health care purchases, while households cover roughly a third and other sources cover the remainder.[24] There can be **insufficient stocks of essential medicines (including some antibiotics), lack of basic equipment, transport and communication, and shortage of trained staff.**[25]

The Malawian Ministry of Health is guided by the Public Health act (1948), National Health Plan (1986), Essential Health Package and more recent initiatives. Health care provision is organised through a tiered health care and referral system including:

- Mobile clinical posts – for vaccination and nutritional assessments
- Health centres
- District Hospitals
- Tertiary referral centres

Since 2015, patients must go to a district hospital prior to referral to an urban, tertiary referral hospital, of which there are four in Malawi: Lilongwe, Blantyre, Zomba and Mizuzu. The extensive network of health facilities means that means that 81% of the population are reported to reside within 8km of a health facility or other forms of health care infrastructure. However, **inadequate resources pose challenges to health care delivery, especially in rural areas**. Government provided services are free of charge at point of access and account for 63% of available health delivery services.[26] The private sector, (non and limited for profit services), also plays an important role through NGO and faith-based organizations, especially in rural areas.[17]

There are limited data on AMR in Malawi. Microbiological culture and AST are not done routinely due to **lack of financial resources, equipment and personnel**.[27] However, there are **research facilities** investigating AMR, and the University of North Carolina working with a hospital in Lilongwe has reported **high levels (>80%) of resistance to commonly used antibiotics**.[28] The Liverpool School of Tropical Medicine (LSTM) has also reported the emergence of multi-drug resistance in invasive *Salmonella enterica* serovar Enteritidis and *S. enterica* serovar Typhimurium infection.[29] Emerging data from the Malawi-Liverpool Wellcome Trust programme suggest levels of antibiotic prescribing are very high in hospital, and rationalisation of prescribing important.

4.2 Leadership

The **Malawian Ministry of Health is engaging with the Malawi-Liverpool Wellcome Trust programme (MLW)**; a collaboration between the University of Malawi College of Medicine, the Liverpool School of Tropical Medicine (LSTM), University of Liverpool, and The Wellcome Trust. Both WHO and CDC also have programmes in Malawi.

The Malawian government is a signatory of the Integrated Disease Surveillance and Response (IDSR), coordinated and implemented by the Epidemiology Unit at the Malawian Ministry of Health. It has also, as part of its 2014-2019 WHO country cooperation strategic agenda, set strengthening of the country's 'early warning system for preparedness detection and response to emergencies and disease epidemics' as a key priority.[30]

However, an in-depth national surveillance review (2012), reported **that two out of three zones** and **most of the country's districts (78%) did not have surveillance work plans**, leading to a **lack of standardization** (e.g. case definitions), **trained personnel and data reporting and harmonization**. Due to a lack of funds and infrastructure, the Epidemiology unit's IDSR work has largely focused on **outbreak management** (primarily cholera and measles). In the field, Health Surveillance Assistants (HSAs), who work as a link between health facility and community levels, play a key role in passive and active surveillance activities, as well as other health-related activities.[31]

4.3 Training

The **Malawi College of Medicine** trains Malawian medical and public health staff for clinical and research activities and laboratory technicians through national post-graduate programmes. To support technical staff capacity, the MLW research programme **recruits staff and trains them using its in-house** capacity. MLW is currently working with the Malawian Medical College to expand training on AMR as part of a **post-graduate programme**. It has also increased salaries for trained to staff in order to retain them; loss of trained staff to non-public health/research facilities is common, due to wide **discrepancies in salaries between organisations**.

4.4 Quality assurance

The **Public Health Institute of Malawi** was set up in 2012, and part of its strategy was to develop a **National Public Health Reference Laboratory in Lilongwe** mandated to oversee the public health laboratory services throughout the country. This includes microbiology, with its remit being to support national surveys, conduct disease surveillance, **ensure quality assurance**, and offer **confirmatory testing** for other laboratories as well as providing training to personnel.

However, at present the **most advanced microbiology capacity is at the MLW research programme at Queen Elizabeth Central Hospital (QECH) in Blantyre**, which was been conducting blood culture surveillance in adult inpatients since 1998. QECH is the main hospital for an urban, peri-urban and rural population of about one million, and also serves as the referral hospital for the southern region of the country.[32]

Box 5: Malawi has a number of country-specific surveillance programmes and projects, these include

Vaccine preventable disease (VPD) surveillance: Focuses on acute flaccid paralysis, measles, and neonatal tetanus, coordinated by the national Expanded Programme on Immunisation. Electronic and paper based records, guidelines and reference materials are reported but there are serious gaps in data quality and harmonization.

Paediatric bacterial meningitis (PBM) sentinel case-based surveillance: Data have been obtained from sentinel surveillance at QECH as part of MLW work.

Rotavirus surveillance: Initiated in 1997 through research work, the consistency and degree of case-based surveillance data has since fluctuated.

HIV surveillance: HIV sentinel surveillance was conducted in over 54 sites in 2007 and the behavioral surveillance survey most recently in 2013-14

TB/MDR-TB surveillance: Annual MDR-TB surveillance data have been provided by the MoH to the WHO since 2007. HSAs play an important role in TB surveillance through passive case finding.

Malaria surveillance system: The National Malaria Control Programme gathers data through passive surveillance of inpatient and outpatient malaria cases and sentinel surveillance. However, its 2011-15 strategic plan states it is limited by human resources and in terms of a robust surveillance system. The Malawi International Center of Excellence for Malaria Research (ICEMR) has conducted biannual community-based household surveillance in low and high transmission settings and seasons.

4.5 Surveillance functions

Laboratory infrastructure and logistics

Laboratory capacity within MLW research laboratories is high, with procedures (registration, electronic laboratory information systems, transport, quality control and external quality assurance) in place for blood and cerebrospinal fluid surveillance, which is done for adults and children admitted to the QECH, see Box 6.

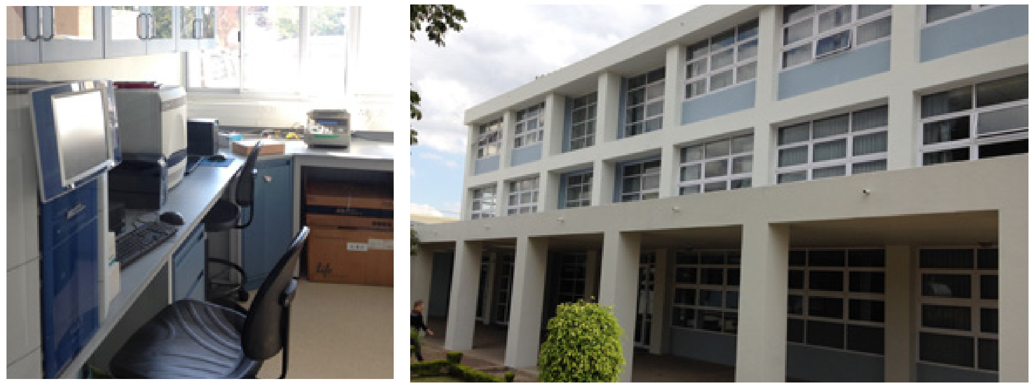

MLW laboratory and research building

In contrast there is **no capacity for blood culture processing in district level hospitals**, although there has been **transfer** of blood culture bottles from a district hospital **for processing in MLW** (mainly for *Salmonella* Typhi and Paratyphi surveillance).

Capacity outside of research programmes at a district level is limited to microscopy and Gram/acid-fast staining, usually stool, urine, sputum, and in some cases, cerebrospinal fluid (when reagents are available). At health centres, diagnostics are largely limited to rapid diagnostic tests for malaria. There has, however, been capacity development for TB diagnostics (GeneXpert) through the national TB programme. There is no reliable electricity supply and generator back-up for laboratory facilities is rare outside of major urban centres.

Box 6: Laboratory capacity in Malawi Liverpool Wellcome-Trust Programme

In QECH (Blantyre) samples are registered by the hospital (**laboratory information management service**) and blood cultures and cerebrospinal fluid (CSF) are transferred across to the MLW laboratory for processing.

Blood cultures and CSF are processed using **automated BACTEC machines** with **standard methods for identification** when blood cultures flag positive.[33] Other samples are processed for research purposes. Selective agars are made in-house and chromogenic agars are available (although infrequently used due to cost). Reagents and consumables are **procured internationally** (through Liverpool University) supporting reliable stocks.

Antimicrobial susceptibility testing is done using **disc diffusion methods**. This includes: amoxicillin, chloramphenicol, oxacillin, ceftriaxone, ciprofloxacin and gentamicin. Zone sizes are recorded and S/I/R assigned based on CLSI guidelines. E-tests are available for use in some contexts (e.g. ciprofloxacin for *Salmonella* spp.) Bacterial isolates from blood cultures are stored in **-80°C freezers.** Inventories are currently paper/spreadsheet based.

The laboratory has a sophisticated **double generator back-up system** and **security is with swipe-card access**. Staff in the MLW unit have appropriate access to **personal protective equipment (PPE)**, including gowns, disposable gloves and eye protection. **Class II biosafety cabinets** have been installed, which are well-maintained with **reliable equipment servicing contracts** with external partners.

Clinical surveillance capacity

Clinical management is based on syndromic diagnoses in Malawi. Guidelines for adult diagnoses are currently being developed at QECH with MLW.

Clinical surveillance data for the Ministry of Health are recorded with a web-based District Health Information System (DHIS2), developed to allow reporting on IDSR, malaria, VPD surveillance, pneumonia and others.[34] However, there are major challenges for the implementation and maintenance of Malawi's health information system, which limit its functionality and the quality of data that it produces.[35] These include: **lack of necessary equipment and communication infrastructure; inconsistent supply of electricity and internet; limited data analysis; and a lack of human resources and supervision**.

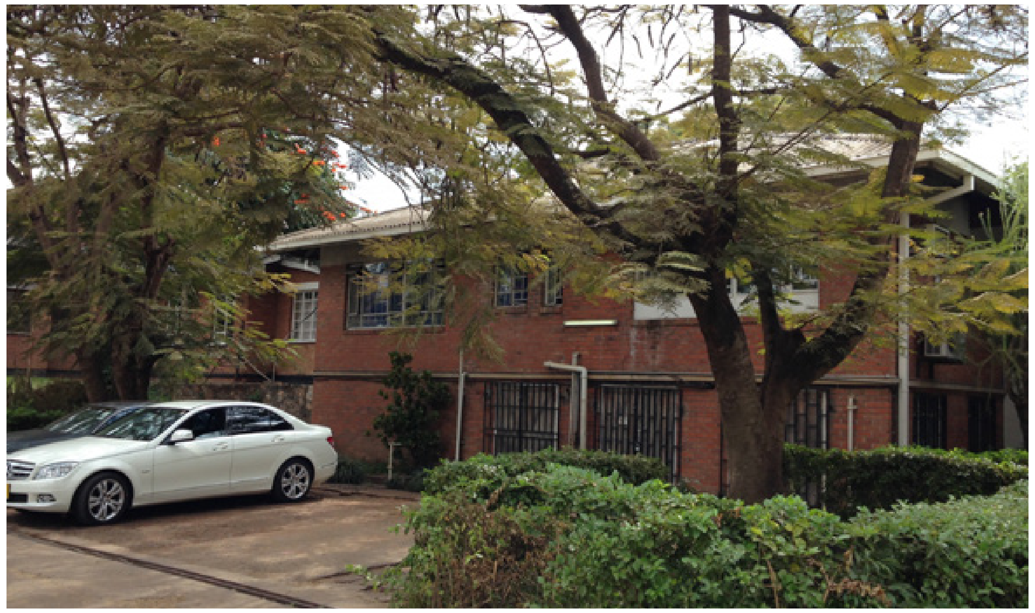

Queen Elizabeth Central Hospital, Blantyre

In Blantyre, the collaboration between MLW and QECH has allowed the development of clinical surveillance in QECH. Clinical data on all medical admissions are stored in an electronic database. The clinical database includes basic demographic data, HIV status and the physician-assigned diagnosis. Data linkage is difficult due to reliance on patient name/date of birth as the identifier, and manual data entry issues. Implementation of electronic patient healthcare identifiers (the "Surveillance Programme of IN-patients and Epidemiology" [SPINE] ID), to register samples has been challenging.[36]

Population: health care utilisation and catchment

Health care utilisation varies in Malawi. This may be related to distance, and rural residence[37] as well as expectation of the standard of care.[37] MLW is developing mHealth to improve understanding of health care utilisation. There is a national population census every 10 years (1998, 2008), and in Blantyre this is used to estimate minimum incidence of blood stream infections. There have also been intermittent censuses/enumerations around more peripheral health centres for research purposes so incidences can be estimated.

4.6 Summary

Malawi faces substantial challenges due to **extremely limited country resources, which reduces health system capacity**. However, whilst it does **not have the capacity for a comprehensive AMR surveillance system**, it has been developing institutional infrastructure and policy for AMR surveillance. It also has considerable capacity through **international collaboration and an academic centre of excellence undertaking microbiological surveillance as part of a research programme.**

In order to take advantage of available research infrastructure (i.e. sentinel surveillance capacity beyond QECH), the following should be considered for new sites:

Whether current capacity is representative of its area

- Is the catchment population well defined? This is likely to be a setting where most of the population will use the clinical facility, linked to good population denominator data (e.g. a recent census)
- Is the access to clinical care open to all, and to what extent are health resources used by patients who are sick?
- Are the results generalisable to the province or region and are they likely to be perceived as credible by national decision makers?

The current AMR surveillance is conducted at the referral hospital (QECH), and does not include sampling in private clinic patients which limits the representativeness of the sampled patient population in relation to Blantyre district as a whole. It is therefore important to consider supporting surveillance capacity development in other sites, using, if possible the expertise available in country.

What are the needs for investment/enhancement in this setting

- Is clinical surveillance of adequate quality?
- Is there associated microbiological surveillance and if not, what would be required to establish this?
- Are the clinical and microbiological records linked and if not, what would be required to establish this?

Clinical surveillance and sampling is being strengthened with triage nurses to support systematic investigation in QECH. Microbiological surveillance is very strong, but electronic linking of clinical and microbiological databases is needed at this site and extension sites.

Whether the hospital is connected to national institutions, and regional networks (WHO-AFRO)

- Establish data flows to Ministry of Health, WHO-AFRO
- Link to Regional quality assurance
- Establish an MOU to form a regional repository of isolates
- Link to network activities, communication and governance

There is growing collaboration between MLW, QECH and the Ministry of Health in Lilongwe, which would be needed to support extension of AMR surveillance sites.

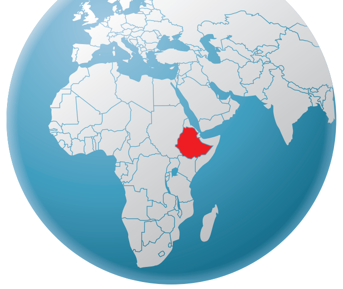

5 Case study: Ethiopia

5.1 Context

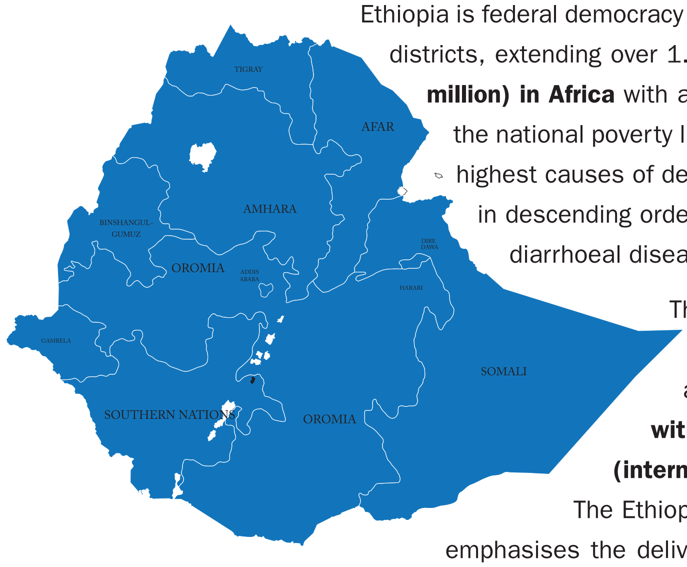

Ethiopia is federal democracy and the country is divided into nine regions, 62 zones and 523 districts, extending over 1.1 million km^2.[38] It **is the third most populated country (99.5 million) in Africa** with a GDP/capita of $619.[39,40] A third of its population lives below the national poverty line with 66% having no access to piped water.[41] Four of the five highest causes of death in Ethiopia are attributed to infectious diseases. They are, in descending order of proportion of overall deaths: lower respiratory infections, diarrhoeal disease, HIV/AIDS, TB and stroke.[42]

The Ethiopian government spends **~4.9% of its GDP and 15.7% of its total annual expenditure on health**.[19,23] This is approximately a third of the country's total health expenditure, **with households paying a further third, donors and NGOs (international and national) 37% and private funds and employers 2%.** The Ethiopian Federal Ministry of Health's (FMoH) National Health Policy emphasises the delivery of health through a **politically and fiscally decentralised health system to its nine regions**. Regional disparities in spending exist due to differences in regional governments' management of federally provided funds.[38]

Figure 4: Divisions of Ethiopia's health system.[44]

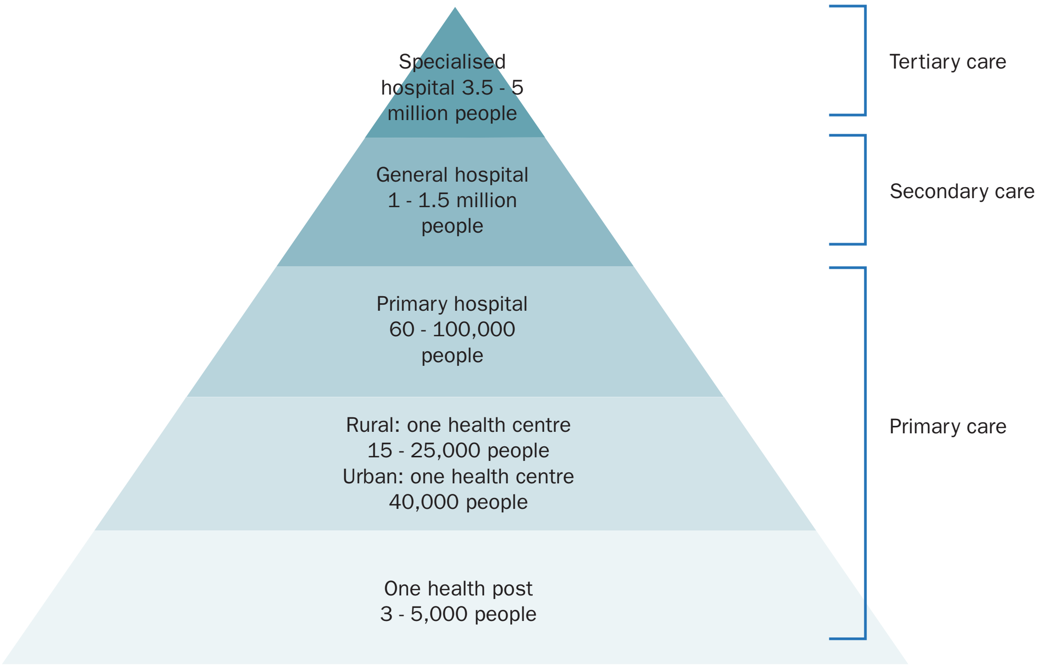

Ethiopia's health care provision is divided into a **three tier system** (see Figure 4).[44] Patients are referred from health posts to health centres, from health centres to district hospitals, and then on to tertiary referral centres; with the exception of urban populations, who can attend tertiary referral centres directly. However, there is a **deficit of health professionals** in Ethiopia. For example, estimates per 10,000 people are at <0.5 physicians, <0.5 pharmaceutical personnel, <0.5 environmental and public health workers, and 2 nursing and midwifery workers, which fall well below the WHO recommended minimum threshold (23 doctors, nurses and midwives per 10 000 population) of health workers necessary to deliver essential maternal and child health services.[45]

Many of the **tertiary referral hospitals are affiliated to universities**, with training programmes for clinical and laboratory staff (e.g. Hiwot Fana hospital in Harar and Haramaya University's College of Health Sciences; Black Lion Hospital and Addis Ababa University's School of Medicine). **Partnerships** with external/international scientific/clinical staff, research and healthcare development organisations also occur (e.g. Black Lion and Yekatit 12 hospitals' affiliation with Fredskorpset [FK Norway; http://www.fredskorpset.no]).

Recent studies in Ethiopia suggest that in general, **medical staff have limited knowledge and awareness of AMR**,[46,47] and data are limited,[48] with independent AMR prevalence surveys as part of student research projects with AHRI, and a 2009 Drug Administration and Control Authority (DACA) baseline survey.[48,49]

5.2 Leadership

Prior to 2009, Ethiopia had limited policies regulating antibiotic use and addressing AMR. Following the first **national 'Antimicrobials Use, Resistance and Containment Baseline Survey' in 2009,** Ethiopian Food, Medicine and Healthcare Administration and Control Authority (FMHACA) developed **a national strategic framework for prevention and containment of AMR**, which aimed to establish a national alliance to tackle AMR and outline the **requisite surveillance**, infection prevention and control, rational use of antimicrobial medicines, and research and education. This has been consolidated into a **National Action Plan (NAP) for Combating Antibiotic-Resistant Bacteria (CARB)**.

A number of **national and international stakeholders** have been involved in the development of AMR work in Ethiopia. Prior to FMHACA's development of a national framework, a number of USAID funded partners, including the Alliance for the Prudent Use of Antibiotics (APUA), produced a report on 'Preserving the Efficacy of Antimicrobial Drugs in Ethiopia.' This was based on a **call-to-action workshop, collaborating with Ethiopian stakeholders in AMR**.[50] Many stakeholders continue to be involved.

The **CDC** is currently **working with the Ethiopian Public Health Institute (EPHI) as part of the Global Security Agenda** (GSA). The CDC is supporting the **'training of trainers'** for EPHI staff for the purpose of AMR surveillance and policy development, which **includes laboratory and data-related techniques** (e.g. management, storage and analysis). Regional Laboratory staff also receive intermittent training from the EPHI National Reference Laboratory teams.

FHMACA has been involved in developing Ethiopia's AMR strategy since its initial conception in 2006, including producing a **national framework, drug vendor formularies** and together with Pharmaceutical Fund and Supply Agency (PFSA), is **responsible for procurement**.[51,52] The PFSA, an independent entity but reports to FMOH, is responsible for supplying public health institutions with **quality assured essential pharmaceuticals and laboratory products at affordable prices**.[53] This includes a **national list for procurement of 'essential pharmaceuticals'** and the development of pharmaceutical forecasting plans together with

SUPPORTING SURVEILLANCE CAPACITY FOR ANTIMICROBIAL RESISTANCE

health facilities to meet their needs.[54] To further support this, the PFSA invests in the strengthening and expansion of relevant health services (human resources, transport and logistics).

Stakeholders in AMR in Ethiopia have collaborated in workshops, the national survey and the development of Ethiopia's NAP. **Effective communication** is important, especially in relation to questions of surveillance and research, staff training and protocol development. Central to this, and to achieving synergy with the aims of other stakeholders, is the necessity to **clearly delineate roles and the division of labour** in relation to AMR. It may be productive to learn from stakeholder engagement in **existing country-specific programmes, for example those targeting HIV/AIDS, tuberculosis, and malaria,** although these are largely externally funded they have developed, within the national infrastructure, mechanisms for importing a consistent supply of drugs, reagents and equipment, as well as training staff, conducting surveillance and providing appropriate care and treatment.

5.3 Training

The Armauer Hansen Research Institute (AHRI) is a **nationally and internationally-funded organization that is now the mandated research arm of the Federal Ministry of Health** and **conducts laboratory research and training**, in collaboration with organizations across Ethiopia, including government hospitals, universities and non-governmental organisations (NGOs). It supports AMR-related studies through training and provides **technical guidance and equipment** to postgraduate students from Ethiopian universities. This includes the use of laboratory equipment, as well as statistical and epidemiological analysis. Regional universities are also developing capacity; for example, at Haramaya University, Oromia, the **College of Health and Medical Sciences** is investing in new training laboratory infrastructure, with a view to providing bench space for laboratory science, pharmacology and microbiology trainees. The current training program facilitates bench experience in basic culture-based approaches, staining and microscopy, basic serology and blood count/biochemical testing. The Department of Medical Laboratory Sciences runs a BSc in Medical Laboratory sciences and an MSc in Medical Microbiology (~4 year programmes).

There are challenges to retention of staff in Ethiopia, where loss of staff occurs to **international organizations, as well as through** emigration due to the higher salaries.[55]

This raises important questions when considering the capacity development needed.

5.4 Quality Assurance

The Ethiopian Food, Medicine and Health Care Administration and Control Authority (FMHACA) is responsible for providing **national regulation, standards, licenses, quality assurance and safety control of pharmaceuticals, food, health facilities and health professionals**.[56] For AMR, this involves establishing standard treatment guidelines and developing surveillance strategies, including laboratory guidelines and measures for conducting quality assurance, together with EPHI and PFSA.

The National Reference Laboratory (NRL) at the EPHI, based in Addis Ababa participates in an **external quality control scheme** (the WHO/National Institute for Communicable Diseases (NICD) External Quality Assurance programme), facilitated by the WHO regional office for Africa. This includes evaluating practices for testing bacterial enteric diseases, bacterial meningitis, malaria and tuberculosis.[11] ASM/CDC capacity-building has emphasised the importance of internal and external quality control.

Box 7: Ethiopia has a number of country-specific surveillance programmes and projects, including

TB/HIV sentinel surveillance system: HIV trend data based on unlinked anonymous testing of left over blood in selected antenatal clinics. Initiated in 1989; follows World Health Organization/Joint United Nations Programme on AIDS guidelines. TB was incorporated into this surveillance system after 2008.

Behaviour Surveillance Survey: Setup in 2002 alongside ANC sentinel surveillance to track trends in HIV/AIDs related knowledge, behaviour and attitudes amongst targeted subgroups; last round was conducted in 2005.

HIV passive surveillance system: supported by the health management information systems (HMIS), it is based on reporting of AIDS cases and sexually transmitted infections (STIs) from relevant health facilities; however, the data are subject to considerable underreporting.

Maternal Death Surveillance and Response: Initiated in 2013 by the Federal Ministry of Health with the object of identifying, reporting and reviewing maternal deaths. Data are collected from communities and combined with any relevant data from the respective health facilities, which are responsible for the aggregation and sharing of data with higher level facilities and committees, including feeding the data into the HMIS system for analysis and dissemination.

Malaria surveillance data based on IDSR: based on over 15 indicators collected monthly and, more recently, weekly by the IDSR system from health facilities in zones but also districts, sub-cities, towns and referral hospitals.

Ethiopia is also signatory to the WHO Regional Office for Africa Integrated Disease Surveillance and Response, with standard reporting forms for notifiable diseases at all hospital and health centers (district level); electronic forms are limited to zone and regional levels.[57]

5.5 Surveillance functions

Box 8: Research institution capacity – Armauer Hansen Research Institute

The Armauer Hansen Research Institute (AHRI) has research laboratories on the ALERT Hospital site in Addis Ababa. It has capacity for conventional and molecular microbiology, and a Level 3 laboratory.

AHRI also has infrastructure for storage at +4°C, -20°C and -80°C capacity. There are catalogues of isolates in some cases going back decades, depending on projects and the associated ethical approvals for long-term/repeat use (frequently difficult to secure these permissions). Storage was in cryopreservant, and the inventory for the freezers was electronic (FreezerWorks).

Laboratory infrastructure and logistics

The National Reference Laboratory (NRL) is located at the Ethiopian Public Health Institute in Addis Ababa (see Box 9). **Regional Reference Laboratories (RRL) are located in the major urban centres of the nine regions of Ethiopia**. These regional laboratories are central collection points for samples from district health centres, and samples and isolates can be referred from the RRL to the NRL for further testing.

The RRLs perform culture for CSF, urethral swabs and urines, typically processing a maximum of 5 specimens per week. Microscopy is performed for stool specimens, and acid-fast staining for sputum specimens. Methods are similar to those used in the NRL, although there is **no capacity to perform blood cultures** (either manual or automated). In the RRLs, sample registration is paper-based. As with the NRL, **results are reported to patients or relatives**. The results of samples are stored in paper-based format. Facilities for sample storage for ~3 months are at +4°C, -20°C.

Both the National and Regional laboratories undertake **internal quality control** of reagents and assays for culture-based microbiology, using "known" strains. Internal Quality Control (IQC) is undertaken ad hoc. Control processes and results are documented, e.g. fridge temperatures.

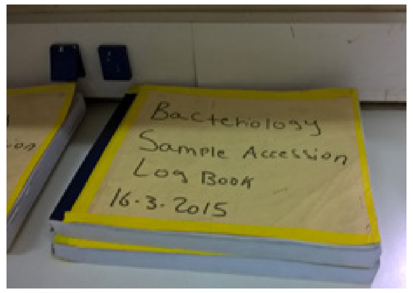

There is **additional capacity** in regional (and hospital) laboratories for processing of specimens for HIV and TB, through **national programmes**, including automated extraction (Abbott 2000) and processing for HIV viral load assessment, and the capacity to quantify CD4 white cell counts.

The district hospital laboratory visited had no culture facilities, but performed **staining and microscopy for CSF and sputum** specimens and urinalysis. Sample registration was **paper based** and results were communicated to relatives. **Reagents were reported to sometimes be in short supply**; including those for Gram staining at the time of visit. There were **limited hand-washing facilities** for lab staff, and no disposable gloves were observed. Quality control was undertaken when reagents were delivered, with access to +4°C refrigeration but no capacity for freezing at -20 or -80°C. There was no back-up generator capacity. Class II biosafety cabinets were installed, but were **not functional** in the hospital laboratory (donated but never properly installed and subsequently used for storage). Infectious waste was typically collected in bags and incinerated on premises at all sites. Liquid waste was disposed of down the sink, with the exception of a University training facility, where drainage was into a septic tank. All hospitals had designated bins (or cardboard boxes) for the disposal of sharps – these were also incinerated.

Private laboratories also exist in Ethiopia; the regulation of these is unclear and examples were not assessed during this visit. They do not contribute to current national bacteriological surveillance.

Box 9: National reference laboratory capacity

The **NRL is based in Addis Ababa** and mainly **processes samples** from patients who are advised to attend NRL rather than samples or isolates sent; blood, urine, cerebrospinal fluid (CSF), ear/nose/throat (ENT), and genital specimens.

Requests are **paper based**, with forms documenting **basic demographic information** (name, date of birth and sex, as well as clinical diagnosis). Samples are assigned a laboratory number for processing; the outcomes of laboratory tests are documented on the request form, as well as in **large laboratory logbooks** (single copy; stored in drawers). Limited electronic data entry is undertaken and clinical details are not stored.

Isolate species identification is performed using **agar-based, and bench testing approaches**, including Gram staining, India ink testing, selective agars, coagulase testing, latex-based Streptococcal grouping and *Salmonella* and *Shigella* spp. serotyping. **Test reagents frequently expire**, however, before they are used up, and timeframes for re-stocking remain unpredictable due to issues with **procurement.**

Selective agars are made in-house and include blood, MacConkey, chocolate, Thayer-Martin, Mueller-Hinton agars and Saboraud slopes for mycological culture. Blood culture medium is manufactured in-house, but frequently clots. Positivity is measured daily by eye. Low numbers of positive cultures are observed. A BacTec automated blood culture machine is available, but the NRL is **unable to procure single-use BacTec media bottles on a reliable basis**. Throughput is low, with a maximum of five blood cultures typically processed per week.

Susceptibility testing is undertaken using **disk diffusion** (CLSI guidelines). Zone sizes are measured and recorded, and a susceptible/intermediate/resistant (SIR) category assigned. E-tests/automated susceptibility testing are not available.

The **NRL has access to +4°C, -20°C and -80°C capacity for isolate storage**. Class II biosafety cabinets are installed and **staff have access to PPE**. The RRL had a separate, walled off room for processing multi-drug resistant TB specimens, but this was not a Level 3 facility.

Clinical surveillance capacity

Ethiopia's Health Information System uses the **health management information systems (HMIS)** with population data from the **Central Statistical Agency of Ethiopia**. The Federal Ministry of Health's Health Management Information System (HMIS) is the **primary platform for reporting and the sharing of data between facilities and government offices**, across the country's different administrative levels. At present, **paper records** are the primary method of record keeping at the district and sub district. However, the systems are being **reformed and standardized**, with plans to expand the electronic system to district levels. HMIS is used to register and **report births and deaths** from all districts, collate **health expenditure** data and provide support for a number of country programmes, including **passive surveillance of TB/HIV**. The IDSR system has been harmonised with the HMIS (e.g. for malaria surveillance) to minimise workload and streamline data collection, linkage and dissemination.[57] At present there are **no mechanisms linking laboratory surveillance to clinical data**, limiting the potential for surveillance data to inform policy and drug formularies.

National evaluations of HIS in 2007 and 2011 concluded that while the quality of data by indicators (e.g. health system and status, morbidity and mortality) was adequate, data management, records and infrastructure were generally poor.[58] Similarly, an international review of the IDSR system (over ten years ago) cited **deficiencies in training, lab reagents, lab and data equipment**, **non-standardised data recording and reporting**, and mechanisms of communication.[59]

Population: health care utilisation and catchment

The most reliable and complete surveillance data are from the capital.[60] In addition, six universities in Ethiopia have **demographic surveillance sites,** collaborating with the US Centers for Disease Control and Ethiopian Public Health Association, and are in the process of developing a **network to standardize and share their results**.

5.6 Summary

Ethiopia is developing a system for AMR surveillance which includes a national reference laboratory and sentinel regional laboratories across the country reporting data (continuous, passive surveillance). At present, whilst capacity is in development, there are some hospitals where the standard of clinical examination is adequate for surveillance, but there is limited microbiology laboratory capacity for processing specimens for culture, particularly blood cultures. Record keeping is not be formalized or electronic, and there is potential for improved communication between the ward and the laboratory to link microbiology data to patients. In strengthening capacity for AMR surveillance, the following should be considered:

Determine whether the hospital(s) are epidemiologically representative

- Is the catchment population well defined, or if private, is there a well-defined population of insured or able to make out of pocket payments (who are not unusual for the country)?

It is important to include a range of health care settings for AMR surveillance; district hospitals, outside of the capital are more generalizable to the Ethiopian population as a whole than urban settings.

Establish surveillance within the hospital

- standardize clinical examination and investigation
- laboratory standardization, training
- record linkage within hospitals

Systems for development would be national guidelines supporting standardised clinical examination and systematic investigation. Laboratory capacity needs strengthening in terms of automated blood cultures to support AMR surveillance to a **core** level. Clinical and laboratory data should be linked, this could be through a double sided form with data for both.

Connect the site to a national and regional network (and on to WHO-AFRO)

- Establish data flows to Ministry of Health, WHO and regional network
- Link to Regional quality assurance
- Establish an MOU to form a regional repository of isolates
- Link to network activities, communication and governance

The Ethiopian Public Health Institute is an arm of the FMoH and combines public health policy making with laboratory capacity in the same institution. Stronger regional links could support capacity development as well as continued partnership with international organizations.

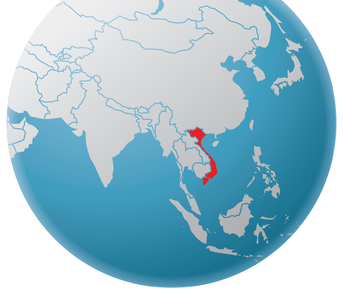

6 Case study: Vietnam

6.1 Context

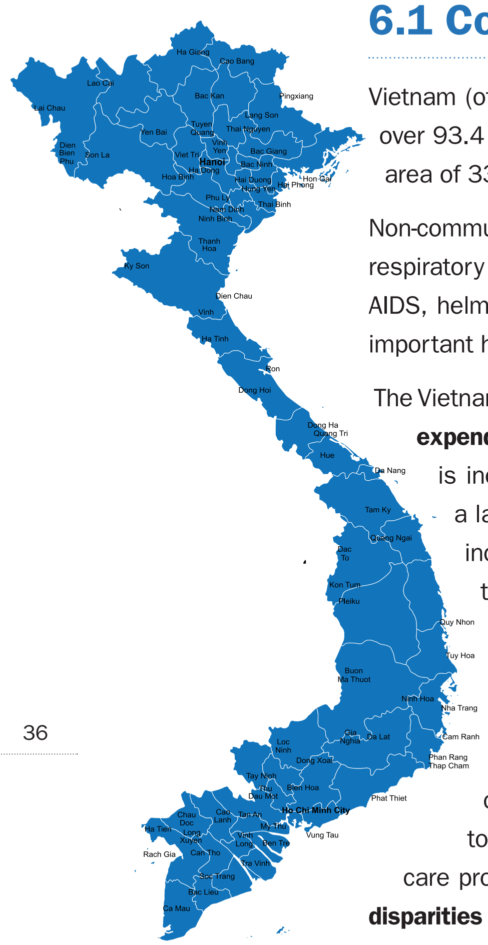

Vietnam (officially, the Socialist Republic of Vietnam) is a **socialist state** with a population of over 93.4 million and is divided into 4 zones, 58 provinces and 5 municipalities, covering an area of 333,210 km^2.[61,62] It has GDP/capita of $2111.[63,64]

Non-communicable diseases are leading causes of death in Vietnam, together with lower respiratory infections and TB.[65] The Vietnamese Ministry of Health (VMoH) also lists HIV/AIDS, helminths, preterm birth complications, influenza and vaccine preventable diseases as important health concerns.[66]

The Vietnamese government is estimated to spend **7.1% of its GDP and 14.2% of its total annual expenditure on health.**[19,23] However, whilst government spending in Vietnam on health care is increasing, **health insurance, user fees and other sources** are increasingly covering a larger portion of the costs of public hospitals. Taken together with **private providers'** increasing role (60% of all outpatient contacts are with private health care providers),[67] these changes suggest a shift to an increasingly **market-guided character**.[68]

The VMoH is responsible for the overall management of health care, with departments and agencies, such as the General Department of Preventive Medicine. It is guided by a 'Master Plan on development of Vietnam's health care system up to 2010 with a vision to 2020.' The implementation of health programs, policies and delivery of care is organized through **administrative divisions and is hierarchically decentralized** to the hamlet/village level (Figure 5). In spite of significant improvements in health care provision and its decentralization, important challenges continue to exist,[68,69] namely: **disparities in health indicators and access to services, especially for rural and highland areas**; lack of human resources and effective management; overcrowded health facilities; poor quality of services; and lack of technical abilities and equipment.

Figure 5: Organisation of health institutions offering curative and preventative services in Vietnam[68]

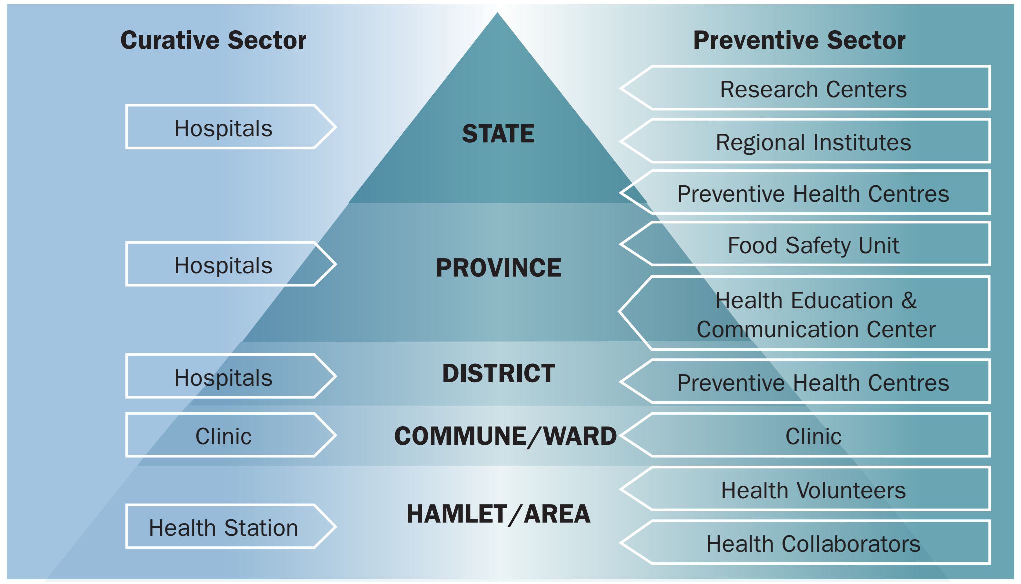

There are **four regional public health institutions responsible for laboratory confirmation, monitoring and guiding disease surveillance and responses**. They include, the WHO and CDC-supported National Influenza Center laboratories at the National Institute of Hygiene and Epidemiology (NIHE) in Hanoi and the Institute Pasteur – Ho Chi Minh City and Nha Trang and the Tay Nguyen Institute for Hygiene and Epidemiology (TIHE).[70] Public health statutory surveillance is predominantly passive and requires local health units to report to higher levels facilities if the number of inpatients crosses a predetermined threshold. In the case of a severe outbreaks or a death, surveillance data sharing is permitted beyond the jurisdiction of the health facilities and preventative medicine centers.[71]

A **situation analysis of antibiotic use and resistance** found high antibiotic consumption and AMR levels in Vietnam.[72] Economic reforms and development in Vietnam have contributed to the deregulation of pharmaceuticals and greater **availability and access to antibiotics.** This, together with a **lack of confidence in laboratory results**,[73] have likely contributed to overuse of antibiotics,[74] with high levels of AMR.[75,76]

6.2 Leadership

Vietnam has the most advanced AMR surveillance system of the three countries visited. It is a member of the Asian **Network for Surveillance of Resistant Pathogens (ANSORP)**, and was the **first country in WHO's Western Pacific Region** to approve an **AMR National Action Plan (NAP)**.[77]

Collaboration across organisations has been strong, through the work of **Ministry of Health** in **collaboration** with **other government ministries** and international organisations, including Oxford University Clinical Research Unit (OUCRU). The Ministry of Health, Ministry of Agriculture and Rural Development, Ministry of Trade and Industry and Ministry of Natural Resources and Environment signed an **aide-memoire in 2014**, committing **to coordinate and collaborate** on the AMR NAP. This led to the creation of a **National Steering Committee** on the prevention of drug resistance, which coordinates AMR activities across different ministries and sectors, and has oversight of many related sub-committees.

The WHO, Japan International Cooperation Agency (JICA), CDC (working via the GSA), the Food and Agriculture Organization of the United Nations and the Oxford University Clinical Research Unit, with the Hospitals for Tropical Diseases in Hanoi and Ho Chi Minh, have all agreed to **provide further technical expertise** for both surveillance and research, to ensure Vietnam's work **aligns with international standards and norms**.[77,78]

These collaborations build on previous work. The Ministry of Health has worked on a Newton Fund project with the National Hospital for Tropical Diseases, Health Strategy and Policy Institute (HSPI), National Institute for Health and Clinical Excellence (NICE) International of the United Kingdom and Oxford University Clinical Research Unit (OUCRU) to update and design AMR relevant policies and guidelines. There are other international universities also working on AMR in Vietnam and the **Vietnamese National Working Group for Global Antibiotic Resistance Partnership (GARP),** includes members from MoH, National Hospital of Tropical Medicine, OUCRU, Ministry of Agriculture and Rural Development and Bach Mai Hospital. This group has conducted policy analysis and capacity assessment in Vietnam through a **situational analysis**, with a focus on antibiotic supply chain and management.[79]

SUPPORTING SURVEILLANCE CAPACITY FOR ANTIMICROBIAL RESISTANCE

Following the NAP and under the oversight of the National Steering Committee, a a national AMR surveillance system is developing capacity and building on the platform which supported the **Vietnam Resistance Project (VINARES)** coordinated by OUCRU in Hanoi and Linköping University, Sweden, collecting AMR data from 16 hospitals.[76] Financial support is currently provided by the Fleming Fund and, with the technical expertise of OUCRU, a National Reference Laboratory in Hanoi is being built.

Box 10: Vietnam has a number of country-specific surveillance programmes and projects, including

Malaria surveillance is coordinated by the National Malaria Control Program (NMCP), but a recent study reported deficiencies in the information system.[80]

The Vietnam National Tuberculosis Control Programme (NTP) is responsible for routine collection of TB surveillance data, which draws on the Viet Nam TB Information Management Electronic System (VITMES); a web-based information system collating data on TB notification and treatment outcomes. Challenges include limited ability to disaggregate data and demographic characteristics, incomplete coverage and notification.[75]

HIV/AIDS sentinel surveillance provides annual prevalence data and will include behavioral questions, replacing the Integrated Biological and Behavioral Surveillance surveys.[81]

Surveillance of agriculture-related infections, specifically foodborne diseases (FBD) is under the Vietnam Food Administration (VFA) and requires notification to the Food Security Agency (FSA). The surveillance data are on outbreaks of food poisoning or gastroenteritis, and in some cases from initial reports in daily media. Other forms of relevant surveillance have not yet been established (e.g. syndromic, behavioural risk factor and AMR).[71]

Vietnam also has **SARS and influenza surveillance system**, which is run through the National Institute of Hygiene and Epidemiology and also includes surveillance for antiviral resistance and data on zoonotic diseases.

6.3 Training

OUCRU Hanoi and Ho Chi Minh provides training for laboratory staff, which started in the 16 hospitals that formed part of the Vietnam Resistance Project, (VINARES) project, and supports **post-graduate training** through research activities.

The CDC (and the WHO) has supported capacity building for surveillance through the **Field Epidemiology Training Programme**, which has trained **epidemiologists, public health staff and veterinarians** working at different administrative levels of the health systems in field epidemiology and One Health epidemiology. They are working towards provincial preventative medicine centres' (PMC) laboratories being accredited to International Organization for Standardisation (ISO).[71]

Much of the CDC's GHSA work has focused on assisting Vietnam to meet the International Health Regulations and Asia Pacific Strategy for Emerging Diseases, with the aim to improve detection and response through improving surveillance and laboratory systems. This has led to the establishment of Emergency Operations Centre together with the **creation of guidelines and relevant training, laboratory system strengthening and the creation of an emergency response information system**.[70] This work has concentrated on SARS, Middle East Respiratory Syndrome, coronavirus, and influenza, but more recently has extended to include Zika virus and **as of the GHSA 2015 five-year action plan, also AMR**.[82,83] The GHSA work has also identified the need for further contributions to the National Laboratory Strategic Plan and the National Steering Committee on Emerging Disease Control and Prevention, as well as further investments in **staffing, infrastructure and reagents**.[70]

6.4 Quality assurance

The national reference laboratory will work towards **international accreditation and will have external quality assurance assessment.** The NRL will support site laboratories to incorporate **external quality assurance schemes (UKNEQAS) in collaboration with the Centers for Disease Control**.

Box 11: National reference laboratory capacity

The Ministry of Health in Vietnam is establishing a National Reference Laboratory in Hanoi. This is with support from the Fleming Fund and Oxford University Clinical Research Unit in Hanoi, the Centres for Disease Surveillance and Control, and the regional World Health Organization. The NRL will be based in a new referral hospital for infectious diseases, currently under construction in Hanoi.

At the NRL there will be capacity for cultures of all specimen types (including automated blood cultures), identification of isolates using standard methods as well as mass-spectrometry (MALDI-TOF) and disc-diffusion testing with AMR susceptibilities reported according to CLSI guidelines. Isolates will be transported to the reference laboratory, and stored in -80°C freezers, with additional capacity for freeze-drying storage. Laboratory data management systems will be electronic, with pod systems to transport specimens from the wards in the new national referral hospital.

6.5 Surveillance functions

Laboratory infrastructure and logistics

The **national reference laboratory is being built** to international standards in Hanoi, and will have considerable capacity, supported by the research and hospital laboratory capacity in the Hospital for Tropical Diseases in Ho Chi Minh and Hanoi. This will include extensive whole genome sequencing and bioinformatics capacity (1000 genomes/year). Following this, the MoH is considering whether in the future additional laboratories will be required at the level of the NRL across the country, for example in southern and central Vietnam. In terms of sites, a **network of up to 30 site laboratories are being connected**; linked to district level hospitals. Cultures are done for blood, cerebrospinal fluid, sputum, urine, and stool. Blood cultures at

sites include automated and non-automated methods. Sample registration is paper-based at site laboratories.

At sites, **susceptibility testing will be done using disk diffusion methods**, with susceptible/intermediate/resistant (S/I/R) profiles reported using **CLSI standards.**

Clinical surveillance

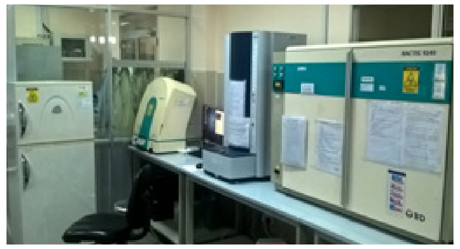

Vietnam's Health Information System (HIS) follows the structure of the four administrative levels relevant to health service delivery (central, provincial, district and commune) with information flow tending to be uni-directional from low to higher organisational levels; while annual reports are limited to the upper three administrative levels (central, provincial and district). Attempts to introduce electronic health records (EHR) have been motivated by the need for accounting.[84] Up until 2010 there was no overall strategy or standardized software for IT and communication and limited investment reduced infrastructure and trained staff, especially at district and commune levels; policy and planning coherence; and technical support. **Further investment and development, including an overall master plan for Vietnam's HIS**, are key aspects for its new **five-year health sector plan**. The CDC (and JICA) has contributed to efforts to develop Epi Info tools appropriate to the Vietnamese HIS for surveillance systems and to enable the real-time reporting of relevant data, including AMR surveillance.[70]

Population catchment

Health care utilisation follows the hierarchical system with referral "up" from first level health care clinics to district and then referral to hospitals. Health care utilisation surveys have and are being done to support research and surveillance in Ho Chi Minh.

The catchment area of the hospitals included in the network are not currently defined. However there have been enumerations within Ho Chi Minh City to support surveillance and research.

Box 12: Research institution capacity – Oxford University Clinical Research Unit (OUCRU)

The combined hospital-OUCRU research laboratories in Ho Chi Minh and Hanoi provide capacity for identification of isolates using standard methods as well as mass-spectrometry (MALDI-TOF); Disk diffusion is also used at the combined hospital-OUCRU research laboratories in Ho Chi Minh and Hanoi, with use of E-tests to report minimum inhibitory concentrations (MICs) for certain antimicrobial-pathogen combinations, reported using Clinical Laboratory and Services Institute (CLSI) standards.

At the OUCRU laboratories (integrated with the Hospital for Tropical Diseases in Ho Chi Minh) there is substantial capacity in term of -80°C storage, but also use of freeze-drying methods which allows bacterial isolates to be stored in cupboards at room temperature.

There are electronic clinical databases at the Hospitals for Tropical Diseases in both Hanoi and Ho Chi Minh, which can be linked to laboratory results. Bespoke systems are used, primarily for research.

6.6 Summary

Vietnam has established a national infrastructure for AMR and has worked with regional networks and international collaborators to develop a surveillance system including a national reference laboratory and network of laboratories attached to district hospitals.

The hospitals are public and broadly representative, there are functioning microbiology laboratories and the physical, legal and ethical capacity to link these records for anonymised aggregation at individual case level. In strengthening capacity for AMR surveillance, the following should be considered:

Consider the hospitals in the network

- is the drainage population well defined?
- how do they perform on a clinical/microbiology audit?
- is there a local champion to lead the site?
- are clinical/microbiology records adaptable for linkage?

The hospitals in the network are mid points in the hierarchical system in Vietnam and broadly representative. However, there would be considerable benefit from understanding health care utilisation patters and population catchments to support laboratory data interpretation. In addition, given the ready accessibility and high volume use of antibiotics in Vietnam, surveillance may be needed at lower levels of health care.

Establish surveillance within hospital

- standardize clinical examination and investigation
- laboratory standardization, training
- record linkage within hospitals

It is important that clinical examination and investigations are strengthened in the AMR surveillance system, to have the benefits of an integrated model, as well as the strengthening of laboratory microbiology services.

Build a network within country (and on to WHO)

- record linkage between hospitals
- building network structure, activities, communication and governance
- establish external quality assurance (ideally through a national public health laboratory)
- establish a repository for collation and investigation of isolates (e.g. for genomic analyses)

Many of this activities are in place in Vietnam, supported by strong national leadership and collaboration, and a hierarchical public health care systems where national strategies can be implemented into care.

7

Costing AMR surveillance

7.1 Scope

The costing methodology follows a 2-dimensional modular framework, described in detail in section 2.4 and 2.5. Costs for setting up an integrated model sentinel site AMR surveillance system in a LMIC, and supporting it over the course of 5 years were calculated. The model does not, however, include the potential economic benefits (cost savings) from a well-functioning AMR surveillance system, for example savings from better informed clinical care or increased responsiveness to AMR emergence.

The model is flexible, based on an assumption that each country would have a number of sentinel sites (depending on population size and epidemiological & demographic diversity) with clinical and laboratory surveillance taking place in each site. An assumption was made that a country would aim for one surveillance facility per 5 million population, with a minimum of five surveillance facilities per country. This would mean that Kenya would aim for 10 sentinel sites, Ethiopia 20 sentinel sites, Malawi 5 sentinel sites and Vietnam 19 sentinel sites. To implement a surveillance system with such a large number of facilities is very costly. Therefore cost results are presented for varying number of sites per country (2, 5 and 10), rather than for the target number of sites, with a focus on costing inputs required to achieve surveillance at level 3 (a **core** level) and level 4 (an **extended** level).

7.2 Description of inputs for each function

For a sentinel site (functioning as per Table 2) each sub-function was broken down into all input activities and resources required to achieve the different levels of surveillance sophistication (**core** and **extended**) and summarized under "interventions" below each sub-function in:

- Table 4A for clinical surveillance
- Table 4B for laboratory surveillance
- Table 4C for isolate storage and transport
- Table 4D for data system & review

Sub-functions and their respective interventions were coded in the same colour with different shades in the tables. A number of inputs were costed independently of the sub-function framework, for example in table 4B personnel costs required for laboratory surveillance or the cost of setting-up a laboratory including the cost of building and equipment. They are presented directly as inputs. The additional demographic component to surveillance was costed as an initial and final population enumeration round in year 1 and 5. Inputs of activities and resources required at the national surveillance level (see Table 3) are shown for each function in Table 5.The inputs described in table 4A to 4D and at national level in table 5 were used to cost the surveillance components by determining quantities and unit costs at different levels of surveillance sophistication.

A detailed costing for each input can be found (Costing Annexe), where costs are estimated for each input by year, across a five year period. In worksheet "country code input" the country for all calculations can be altered. At the time of writing the report personnel costs for Malawi have not been made available and therefore the final costs presented in this report exclude Malawi.

Table 4A: Clinical surveillance: Clinical admission, examination and investigation

Clinical admission assessment and investigation				
	Level 1	Level 2	Level 3 - CORE	Level 4 - EXTENDED
Clinical admission assessment	Clinical history and examination incomplete	Clinical history and examination, but no algorithms in use	Systematic clinical history and examination according to clinical algorithms in core patient group (< 5 years) and WHO core pathogens excluding Neisseria gonorrhea; only BC	Systematic clinical history and examination according to clinical algorithms in all target patient groups; includes BC, CSF, stool, urine and swap sampling
Interventions	For surveillance zero costs assumed	Level 1–>2:<br>strengthening of clinical leadership locally;<br>strengthening of local clinical training;<br>employement of more senior HCWs;<br>–> considered essential for a functioning health facility<br>For surveillance zero costs assumed	Level 2–>3:<br>Start-up: Writing of national paediatric guidelines or protocols of clinical care: selection of a national taskforce of 30-50 pediatrician and senior nurses, organization of first workshop with duration of 2 weeks; designing and editing of guidelines; printing and distribution of booklets (will depend on HCW force in country); raise awareness and training of HCW force<br>Recurrent: Subsequent triannual review workshops for adaptation of guidelines with pediatric taskforce; adaptation of previous guidelines; printing and distribution of booklets<br>Employement of sufficient and well qualified HCWs (see Personnel + cost worksheet)<br>! Costs of minimum required staff have not been included in the cost calculation, except if for a surveillance specific activity!	Level 3–>4:<br>Start-up: Writing of national adult guidelines or protocols of clinical care: selection of a national taskforce of 30-50 clinicians and senior nurses, organization of first workshop with duration of 2 weeks; designing and editing of guidelines; printing and distribution of booklets; raise awareness and training of HCW force<br>Recurrent: Subsequent triannual review workshops with adult taskforce; adaptation of previous guidelines; printing and distribution of booklets (will depend on HCW force in country); HCW able to perform CSF sampling required in each facility (trained on the job)<br>Employment of sufficient and well qualified HCWs (see Personnel + cost worksheet)<br>! Costs of minimum required staff have not been included in the cost calculation, except if for a surveillance specific activity!

Table 4A: Clinical surveillance: Clinical admission, examination and investigation (cont)

Clinical admission assessment and investigation (cont)				
	Level 1	Level 2	Level 3 - CORE	Level 4 - EXTENDED
Clinical data	Documentation incomplete	Paper based documentation or electronic system without linkage	Documentation into paper system using unique alphanumeric identifier which links to lab isolates	Standardised documentation into electronic system using unique alphanumeric identifier which links to lab isolates
Interventions	For surveillance zero costs assumed	Level 1–>2:<br>For surveillance zero additional costs assumed as system without linkage not desirable for surveillance	Level 2–>3:<br>Start-up: Set up of paper based system with labels that links to lab isolates and storage; Writing and Start-up of SOPs; training workshop for clinicians, lab staff and administrators;<br>Recurrent: Annual quality assurance checks of documentation process	Level 3–>4:<br>Start-up: Training of all clinical staff (physicians, nurses, nursing assistants) for 3 days per staff<br>Recurrent: in house refresher trainings 1 hour per staff per year
Clinical investigation	Clinical investigations ad-hoc	Clinical investigation based on clinical findings but not standardised	Standardised clinical investigation based on clinical findings (fever) in core patient group (<5 years)	Standardised clinical investigation based on clinical findings (fever) in all target patient groups
Interventions	For surveillance zero costs assumed	Level 1–>2:<br>Employment of well trained and motivated doctors<br>For surveillance zero additional costs assumed	Level 2–>3:<br>Miminum number of health care workers required as outlined in worksheet “HR + costs”; standardized guidelines of clinical care as above for pediatric care	Level 2–>3:<br>Miminum number of health care workers required as outlined in worksheet “HR + costs”; standardized guidelines of clinical care as above for adult care in addition to pediatrics
Training and quality assurance	Not done	Ad hoc	Routine training to surveillance SOPs	Quality assurance of clinical data with external assessment
Interventions	For surveillance zero costs assumed	For surveillance zero costs assumed, training would be ad hoc, most likely on the job and initiated as well as conducted locally in most instances	Level 2–>3:<br>Start-up: Writing of pediatric surveillance SOPs (e.g. taking blood cultures, operating and maintaining equipment, investigations such as microscopy, blood count, full blood glucose; recognition of sick child according to clinical guidelines, medical history, clinical investigations according to clinical guidelines); initial training to surveillance SOPs<br>Recurrent: refresher training to pediatric surveillance SOPs	Level 3–>4:<br>Start-up: Writing of adult surveillance SOPs (e.g. taking blood culture, operating and maintaining equipment, investigations such as microscopy, blood count, blood glucose; recognition of sick person according to clinical guidelines, medical history, clinical investigations according to clinical guidelines); initial training to surveillance SOPs; external quality assurance (EQA) of clinical data through an annual consultant visit per country, revision of clinical data at national level and visit to 2 sites, standardization of clinical data<br>Recurrent: refresher training to adult surveillance SOPs, annual EQA of clinical data at national level and visit to 2 sites

SUPPORTING SURVEILLANCE CAPACITY FOR ANTIMICROBIAL RESISTANCE

Table 4B: Laboratory surveillance: Isolate identification and susceptibility testing

**Isolate identification and susceptibility testing**				
	Level 1	Level 2	Level 3 - CORE	Level 4 - EXTENDED
National guidelines of good laboratory practice	no national guidelines in place	no national guidelines in place	no national guidelines in place	National guidelines written by a national taskforce
Interventions	n/a	n/a	Level 2–>3:<br>Start-up:<br>Recurrent:	Level 3–>4:<br>Start-up: Writing of national laboratory guidelines of good laboratory practice including laboratory equipment and building, sample transport, registration, culture, susceptibility testing, reporting, laboratory safety and biosafety, Q&A, data information to clinician, waste management, national reference laboratory, and general good laboratory practice based on international guidelines and standards: selection of national taskforce of 20-30 people including lab managers, microbiologists and lab technicians; organization of first workshop for taskforce with duration of 2 weeks; designing and editing of guidelines; printing and distribution of booklets; raising awareness and training of laboratory workforce in all surveillance facilities; writing of national Standard operating procedures (SOPs); adaptation of SOPs to facility level<br>Recurrent: Subsequent triannual review workshops with national taskforce: adaptation of previous guidelines; printing and distribution of booklets; raising awareness and training of laboratory workforce in all surveillance facilities<br>Employment of sufficient and well qualified laboratory personnel (see Personnel + cost worksheet)
Set up cost of laboratory	costing not done	costing not done	Cost of building a minimum 80 sqm laboratory with non porous flooring, with minimum equipment for level 3 (see worksheet “equipment”)	Cost of building a minimum 140 sqm laboratory with non porous flooring, ergonomically designed with contained microbiology area, with “dirty corridor” for sample transport and waste management, with minimum equipment for level 4 (see worksheet “equipment”)
Personnel cost	costing not done	costing not done	Minimum personnel cost as indicated for level 3 (see worksheet “personnel + cost”)	Minimum personnel cost as indicated for level 3 (see worksheet “personnel + cost”)
Sample transport from clinical site to laboratory for processing	Samples not transported routinely	Samples transported, no standards in place to guide process	Samples transported according to local standard operating procedures	Samples transported according to international standards for biosafey
Interventions	For surveillance zero costs assumed	costing not done	Level 2–>3:<br>Start-up: Writing and implementing of sample transportation SOPs at local level; training of all personnel involved with sample transport;<br>Recurrent: Usually the laboratory is within or adjacent to clinical site: samples transported by foot<br>If transported by vehicle: assumed samples are added to vehicle already journeying between clinic and laboratory;<br>ice as transport medium and storage containers;<br>no costs of transport added to cost estimate as highly dependant on the set up of surveillance site;<br>Annual re-training of all staff on sample transportation	Level 3–>4:<br>Start-up: Adaptation of national SOPs on sample transportation according to international standards on biosafety to local facility level; training of all personnel involved with sample transport;<br>Recurrent: Usually transported by foot, if transported by vehicle, assumed to be add-on; ice as transport medium and storage containers;<br>no costs of transport added to cost estimate as highly dependant on the set up of surveillance site;<br>Annual re-training of all staff on sample transportation;<br>Neisseria gonorrhea: routine sampling and testing of 5-10 pregnant women per month at ANC department of each surveillance site (all taken on same day); subsequent transport within 4 hours to laboratory; training of relevant staff including clinical, laboratory and transport staff; materials required for transport are CO_2 jars.
Sample registration	No registration system	Paper based registration (logbook)	Local lab data system with manual linkage to clinical data on site	Local lab data system linked to clinical data with aggregation to national network (automated)

Table 4B: Laboratory surveillance: Isolate identification and susceptibility testing (cont)

Isolate identification and susceptibility testing (cont)				
	Level 1	Level 2	Level 3 - CORE	Level 4 - EXTENDED
Interventions	zero costs	Cost of training on logbooks, cost of logbooks 2/year (US$100 each); costing not done	Level 2–>3:<br>Start-up: Programming simple lab data management system without links to clinical data, cost of programming it, cost of training every person (ward, registry, lab) on system (for details see worksheet "data system and review");<br>Recurrent: annual refresher training on lab data management system; dedicated lab assistant who does the sample registration<br>Costs in "data system and data review" worksheet	Level 3–>4:<br>Start-up: Programming more sophisticated lab data management system electronically linked to clinical data and automatically linked to national network; cost of programming it, cost of training every person (ward, registry, lab) on system (for details see worksheet "data system and review");<br>Recurrent: annual refresher training on data management system; dedicated lab assistant who does the sample registration<br>Costs in "data system and data review" worksheet
Culture	No blood culture	No blood cultures	Automated blood culture, with processing the 7 out of 8 WHO priority pathogens according to SOPs (excludes Neisseria gonorrhoeae)	Automated blood culture as well as csf, urine, stool and swab culture, processing all isolates according to SOPs
Susceptibility testing	Not done	Done but not according to defined standards and/ or without SOPs	According to SOPs for WHO 7 out of 8 WHO priority pathogens (excludes Neisseria gonorrhoeae) qualitative testing	According to SOPs for all pathogens
Intervention for culture and susceptibility testing	zero costs	Costing not done, as difficult to define scope of work performed	Level 2–>3:<br>Start-up: Writing and implementing of sample processing SOPs at local level; training of all personnel involved with sample processing;<br>A core equipment and a minimum amount of personnel is required for a reliable blood culture processing and susceptibility testing; See worksheet "equipment" and worksheet "HR + cost" for level 3<br>Recurrent: Annual refresher training for all personnel involved with sample processing; Maintenance and replacement of core equipment (see worksheet "equipment"); ongoing cost per test (excludes cost of equipment and personnel)	Level 3–>4:<br>Start-up: 1. National guidelines on good laboratory practice are required; (details above) 2. adaptation of national SOPs on sample processing to facility level; 3. training of all staff on guidelines and SOPs (for costing of 1-3 see above); 4. Core equipment and a minimum amount of personell is needed for a reliable sample (blood, csf, urine, stool, swap) processing; See worksheet "equipment" and worksheet "HR + cost" for level 4<br>Recurrent: Annual refresher training for all personnel involved with sample processing;<br>Maintenance and replacement of core equipment; ongoing cost per test (excludes cost of equipment and personnel)
Training and quality assurance, accreditation	Not done	Ad hoc training and quality assurance	Routine training to surveillance SOPs	Quality assurance of laboratory with external assessment; accreditation according to ISO 15189
Interventions	level 1–2: zero costs	zero cost assumed	Level 2–>3:<br>Start-up: Cost of writing, approving local SOPs for all laboratory processes; training for each staff;<br>Recurrent: Annual re-fresher training for each staff; adaptation of SOPs; drafting of new SOPs when required; quality assurance done in-house by member of staff; no accrediation process	Level 3–>4:<br>Start-up: Cost adapting national SOPs to local level (costed above), training for each staff (costed above); ISO15189 accreditation intital cost<br>Recurrent: Annual re-fresher training for each staff; adaptation of SOPs; drafting of new SOPs when required (costed above); employment of quality assurance manager (costed above in "personnel");<br>ISO15189 accreditation yearly cost; cost of 2 external quality assurance service providers

Table 4C: Isolate storage (local) and referral to reference laboratory

Isolate storage (local) and referral to reference lab				
	Level 1	Level 2	Level 3 - CORE	Level 4 - EXTENDED
Storage of isolates	No biorepository, or no documentation of stored samples.	Biorepository of isolates, paper-based database with storage of at least a subset of resistant isolates defined by local policy (-20 or -80C)	Biorepository of isolates, with paper or electronic database with storage of all resistant isolates (-20 or -80C) defined by national policy	Biorepository of isolates, with electronic database of all stored resistant isolates defined by national and/or international policy, maintained at -80C.
Interventions	For surveillance zero costs assumed	Level 1–>2:<br>costing not done	Level 2–>3:<br>Start-up: -80C freezer and -20 freezer, already costed in worksheet “lab cost detail”; set-up of local biorepository database, already costed in worksheet “Data system and review detail”<br>Recurrent: maintenance of freezers; (already costed in worksheet “lab cost detail”	Level 3–>4:<br>Start-up: writing of national guidelines and SOPs for the storage of isolates (costed in worksheet “lab cost detail”); -80C freezers (costed in worksheet “lab cost detail”); set up of electronic system with linked biorepository database (costed in worksheet “data system and review”)<br>Recurrent: maintenance of freezers, dedicated lab technician for biorepository; (all costed in worksheets “lab cost detail”)
Transport to reference lab	Isolates are not transferred to reference lab	Isolates are transferred to ref lab on an ad-hoc basis	Isolates are transferred to ref lab at least annually but not according to defined SOPs	Isolates are transferred to ref lab at least annually according to international standards for biosafey
Interventions	For surveillance zero costs assumed	Level 1–>2:<br>costing not done	Level 2–>3:<br>Start-up: no start up interventions required<br>Recurrent: Annual transfer of isolates to national reference laboratory, cost of transport by plane and/or car; cost of transport equipment	Level 3–>4:<br>Start-up: writing of national guidelines and SOPs for the transportation of samples according to international standards of biosafety (already costed in worksheet “lab cost detail”)<br>Recurrent: Twice a year transfer of isolates to national reference laboratory, cost of transport by plane and/or car, cost of transport equipment
Training and quality assurance	Not done	Ad hoc	Routine training for isolate storage and transport SOPs	Routine training for isolate storage and transport SOPs;<br>Quality assurance of lab biorepository with external assessment
Interventions	For surveillance zero costs assumed	Level 1–>2:<br>costing not done	Level 2–>3:<br>Initial training and annual refresher training of all laboratory staff on local isolate storage and transport SOPs	Level 3–>4:<br>Initial training and annual refresher training of all lab personnel on national isolate storage and transport SOPs; External quality assurance (EQA) of biorepository through an annual consultant visit per country, revision of biorepository database at national level and visit to 2 sites, standardization of biorepository database data

Table 4D: Data system & review

Data system and review				
	Level 1	Level 2	Level 3 - CORE	Level 4 - EXTENDED
Data system	none	not done	Local lab data system with manual linkage to clinical data on site; system not linked to national network; paper based or electronic biorepository database that is not linked	Local lab data system electronically linked to clinical data and automatically linked to national network
Interventions	zero costs	zero costs	Level 2–>3:<br>Start-up: Programming of simple lab data system (i.e. filemaker), not linked to clinical data system; training of all relevant staff on data system<br>Recurrent: On going maintenance of lab data system; yearly in-house refresher training	Level 3–>4:<br>Start-up: Programming of user-friendly data management system at national level, linking clinical and laboratory data in each facility, but also with linkage to the national network; System with ability to create automatic reports; adaptation of data management system to requirements in each facility; Training of facility staff (clinical, laboratory, QA) on use of data management system;<br>Recurrent: On-going maintenance and support of data management system; yearly in-house refresher training
Data use	Limited	Data used for individual clinical care; hospital review for treatment guidelines	Submit to national network	Submit to international network (through country level surveillance)
Interventions	zero costs	Level 1–>2:<br>costing not done	Level 2–>3:<br>Start-up: External training of one member of staff per facility in analyzing data and creating reports; at facility level there will be one member of staff who will is given responsibility to submit report<br>Recurrent: Annual online refresher training in analyzing data and creating reports	Level 3–>4:<br>Start-up: Data management system programmed with ability to create automatic reports, which are sumitted to national network on a monthly basis; from national network submission to international network<br>Recurrent: at facility level one member of staff is given responsibility to submit report (probably senior clinical microbiologist)<br>no additional costs assumed
Data linkage	No linkage	No linkage	Manual linkage	Automated linkage
Interventions	zero costs	zero costs	Level 2–>3:<br>No intervention needed	Level 3–>4:<br>No additional intervention needed, data system is programmed with linkage between the modules;
Data governance	No data sharing policy	Local data sharing policy (in-hospital)	Data sharing policy and agreements in place in collaboration with the Ministry of Health and/or national public health institute (see national level)	Data sharing policy and agreements in place in collaboration with the Ministry of Health and/or national public health institute (see national level)
Interventions	zero costs	Level 1–>2:<br>Agreemement of data sharing at local level; drafting and signing of data confidentiality agreements by all relevant parties<br>costing not done	Level 2–>3:<br>Agreemement of data sharing at national level; drafting and signing of data confidentiality agreements by all parties at facility and national level<br>costing not done	Level 3–>4:<br>Agreemement of data sharing at national level; drafting and signing of data confidentiality agreements by all parties at facility and national level<br>costing not done

Table 5: National Surveillance

**National surveillance**					
Level of surveillance sophistication increasing from left to right					
		Level 1	Level 2	Level 3 - CORE	Level 4 - EXTENDED
Functions	Subfunctions				
Core personnel at national level	n/a	n/a	n/a	Employment of core surveillance staff at national level (assumed the same at level 3 and 4):<br>1 Senior project manager to oversee national surveillance activites<br>1 Senior clinical microbiologist for lead on laboratory surveillance<br>1 Senior medical officer/ consultant for lead on clinical surveillance<br>1 Senior data manager for lead on surveillance data analysis<br>1 Senior database administrator (already costed in system and review<br>1 Communication officer<br>1 Training coordinator to coordinate training programmes with lab, clinical, QA and data lead<br>1 Quality assurance manager to oversee national quality assurance programme<br>2 Administrators to support national surveillance programme	
Leadership	Data analysis	None - no national coordinating body	National coordinating body but limited national data	National coordinating body reviews aggregated data with annual report	National coordinating body reviews aggregated data with annual report
	Data governance	None - no national coordinating body	National coordinating body but limited national data	National standards for data governance, legislation	National standards for data governance, legislation
	Assessment of evidence	None - no national coordinating body	National coordinating body but limited national data	National coordinating body reviews aggregated data with expert advice where needed and regional network	National coordinating body reviews aggregated data with expert advice where needed and regional network
	Intervention	None - no national coordinating body	National coordinating body but limited national data	Surveillance data drive national policy and international policy	Surveillance data drive national policy and international policy
Interventions	-	n/a	n/a	Start-up: Set up of national coordinating body consisting of people with clinical, laboratory, epidemiological, statistical, legislative and policy experience (not costed)<br>Legal consultancy to support drafting of national data governance agreements between sites and national coordinating body;<br>use of international bodies like WHO for international data governance agreements<br>Recurrent: Regular quarterly national coordinating body meetings and emergency meeting in case of threat (not costed)	
Training	Clinical	None	None	Training programme "train the "trainers" in core clinical surveillance procedures	Established national and international standards and accreditations to deliver training programmes
	Laboratory	None	None	Training programme "train the "trainers" in core lab surveillance procedures	Established national and international standards and accreditations to deliver training programmes
	Data	None	None	Training programme "train the "trainers" in core data surveillance procedures	Established national and international standards and accreditations to deliver training programmes
Interventions	-	n/a	n/a	Start-up: National training programme established and delivered by core national surveillance personnel (costed above); site specific training costs already costed in other worksheets ("clinical", "lab", "isolate storage", "data system and review"); national lead will receive external training and be supported by international groups (academic institutions or WHO)<br>Recurrent: Continued external support of training progammes; continuation of in-country training in the respective areas<br>No additional costs added here	

SUPPORTING SURVEILLANCE CAPACITY FOR ANTIMICROBIAL RESISTANCE

Table 5: National Surveillance (cont)

National surveillance (cont)					
Level of surveillance sophistication increasing from left to right					
		Level 1	Level 2	Level 3 - CORE	Level 4 - EXTENDED
Functions	Subfunctions				
Quality Assurance	Clinical	None	None	Annual audit of clinical surveillance at sites	Quarterly audit of clinical data submitted through automated systems and comparison with other sites
	Site lab	None	None	Annual audit of laboratory standards	QA assessment of laboratory site to international standards
	Reference lab	No reference laboratory in-country	National reference laboratory but no standard protocols for AMRS	National reference laboratory, does QA with repeat testing of a subset of isolates	National reference laboratory, does QA with repeat testing of a subset of isolates, can do molecular testing (eg PCR) and/or local or international collaboration in place for next generation sequencing of isolates
	Data	None	None	Annual audit of data systems at sites	Support for automated sharing of data from sites for national aggregation
Interventions	-	n/a	n/a	Employment of one Quality assurance manager who takes the national lead and works together with national lead on lab, clinical surveillance and surveillance data (costed above in personnel)	
Setting up and running cost of national reference lab				Costs of personnel and starting a national reference laboratory including equipment; national reference lab assumed to function like a "level 4 laboratory" at level 3 (see equipment worksheet for details)	Costs of personnel and starting a national reference laboratory including equipment; national reference lab assumed to function like a "level 5 laboratory" at level 4 (see equipment worksheet for details)
National reference lab	Storage of isolates	No biorepository, or no documentation of stored samples.	Biorepository of isolates, paper-based database with storage of at least a subset of resistant isolates defined by local policy (-20 or -80C)	Biorepository of isolates, with paper or electronic database with storage of all resistant isolates (-20 or -80C) defined by national policy	Biorepository of isolates, with electronic database of all stored resistant isolates defined by national and/or international policy, maintained at -80C.
	Transport to reference lab	Isolates are not transferred to reference lab	Isolates are transferred to ref lab on an ad-hoc basis	Isolates are transferred to ref lab annually	Isolates are transferred to ref lab at least annually according to international biosafety standards
Interventions	-	n/a	n/a	Equipment for isolate storage costed above as equipment in "setting up costs of national reference lab"<br>Data base and data system described and costed in worksheet "Data system and review"; Transport from site to national reference laboratory including equipment already described and costed in worksheet "isolate storage"; national reference lab assumed to function like a level 4 laboratory at level 3 (see equipment worksheet for details)<br>No additional costs added here	Equipment for isolate storage costed above as equipment in "setting up costs of national reference lab"<br>Data base and data system described and costed in worksheet "Data system and review"; Transport from site to national reference laboratory including equipment already described and costed in worksheet "isolate storage"; national reference lab assumed to function like a level 5 laboratory at level 4 (see equipment worksheet for details)<br>No additional costs added here

7.3 Costing results

Costs are dependent on **existing capacity**, **number of sentinel sites** proposed for surveillance, and, across countries, **variability in costs of personnel**.

Table 6 shows the costs for Kenya, Ethiopia and Vietnam for 2, 5, 10, 19 (Vietnam only) and 20 (Ethiopia only) sites. The costs were broken down into the categories clinical, laboratory, national and demographic surveillance, as well as data system & review. The laboratory surveillance cost in table 6 include the cost of the functions "laboratory surveillance at site level", "isolate storage & transport" and the "national reference laboratory". In figures 6 and 7 the costs by function are displayed for Kenya and Vietnam by varying number of sentinel sites (2 and 5 for Kenya, 2, 5, and 10 for Vietnam), assuming five years of surveillance. For example at level 3 the total cost over 5 years for 5 sentinel sites (in addition to national reference laboratory) was £9,298,221, £4,679,193 and £4,344,348 for Kenya, Ethiopia and Vietnam respectively. The costs by year, ranging from year 1-5 are displayed in figure 8 for Kenya, Ethiopia and Vietnam.

The largest contributor to the total cost is **laboratory surveillance at the sentinel site** (including isolate storage and transport to national reference laboratory), together with the **national reference laboratory cost.** These laboratory costs combined range, depending on number of sites and country, between 67% and 77% of the total cost at level 3 and between 78% and 85% at level 4. Within the laboratory cost the largest contributors to the total costs are the initial set up cost of the laboratory, and personnel (for detail see figure 9). The **increase in cost from core laboratory functions to extended laboratory functions is substantial** (Figures 6 and 7) and is driven by the substantial increase in equipment and staff to support the increase in samples being processed (all sample types vs blood cultures), and isolates tested (all isolates vs World Health Organization priority pathogens (excluding *Neisseria gonorrhoeae*)).

The higher costs for Kenya can be explained by the much higher personnel costs in Kenya, which also contribute to a lower share of the total costs in year 1 (around 37% in Kenya, versus around 50% for Ethiopia and Vietnam) due to the high ongoing staff costs.

Within the overall envelope costed for AMR surveillance, **the costs for clinical surveillance, data system and demographic surveillance** (population enumerations at beginning and end for (level 4) extended only) **are a much smaller proportion of total cost than laboratory surveillance**. This is partly because for the clinical elements the costs of clinical staff providing care were not included, as in-post, except where additional clinical staff would be needed to undertake specific activities needed to support surveillance (developing clinical guidelines, adapting national standard operating procedures for clinical surveillance). In contrast, laboratory personnel costs were all included in the costing. However, this likely reflects the real-world situation, in terms of the need for recruitment of laboratory staff.

Table 6: Costs by function for 2, 5, 10 and target number of sites in Kenya, Ethiopia and Vietnam

		**Activity**	**Clinical surveillance**		**Laboratory surveillance***		**Data system and review**		**National surveillance**		**Demographic surveillance**		**Total**	
	Sites**	Level	3-CORE	4-EXT	3-CORE	4-EXT	3-CORE	4-EXT	3-CORE	4-EXT	3-CORE	4-EXT	3-CORE	4-EXT
**Kenya (target N=10)**	2	**£**	**£210,938**	**£230,452**	**£3,719,870**	**£7,308,852**	**£49,904**	**£257,226**	**£1,032,686**	**£1,032,686**	**£580,319**	**£535,643**	**£5,593,717**	**£9,364,860**
		%	*3.8%*	*2.5%*	*66.5%*	*78.0%*	*0.9%*	*2.7%*	*18.5%*	*11.0%*	*10.4%*	*5.7%*	*100.0%*	*100.0%*
	5	**£**	**£242,614**	**£265,129**	**£6,504,910**	**£12,731,723**	**£67,214**	**£304,344**	**£1,032,686**	**£1,032,686**	**£1,450,797**	**£1,339,108**	**£9,298,221**	**£15,672,991**
		%	*2.6%*	*1.7%*	*70.0%*	*81.2%*	*0.7%*	*1.9%*	*11.1%*	*6.6%*	*15.6%*	*8.5%*	*100.0%*	*100.0%*
	10	**£**	**£295,407**	**£322,923**	**£11,146,643**	**£21,769,841**	**£96,065**	**£382,875**	**£1,032,686**	**£1,032,686**	**£2,901,594**	**£2,678,217**	**£15,472,395**	**£26,186,542**
		%	*1.9%*	*1.2%*	*72.0%*	*83.1%*	*0.6%*	*1.5%*	*6.7%*	*3.9%*	*18.8%*	*10.2%*	*100.0%*	*100.0%*
**Ethiopia (target N=20)**	2	**£**	**£134,528**	**£175,889**	**£1,872,496**	**£4,062,905**	**£25,057**	**£126,289**	**£351,299**	**£351,299**	**£354,218**	**£324,545**	**£2,737,599**	**£5,040,928**
		%	*4.9%*	*3.5%*	*68.4%*	*80.6%*	*0.9%*	*2.5%*	*12.8%*	*7.0%*	*12.9%*	*6.4%*	*100.0%*	*100.0%*
	5	**£**	**£159,547**	**£236,039**	**£3,244,766**	**£6,763,768**	**£38,034**	**£169,368**	**£351,299**	**£351,299**	**£885,546**	**£811,363**	**£4,679,193**	**£8,331,836**
		%	*3.4%*	*2.8%*	*69.3%*	*81.2%*	*0.8%*	*2.0%*	*7.5%*	*4.2%*	*18.9%*	*9.7%*	*100.0%*	*100.0%*
	10	**£**	**£201,245**	**£336,287**	**£5,531,883**	**£11,265,205**	**£59,664**	**£241,165**	**£351,299**	**£351,299**	**£1,771,092**	**£1,622,726**	**£7,915,184**	**£13,816,682**
		%	*2.5%*	*2.4%*	*69.9%*	*81.5%*	*0.8%*	*1.7%*	*4.4%*	*2.5%*	*22.4%*	*11.7%*	*100.0%*	*100.0%*
	20	**£**	**£284,641**	**£536,784**	**£10,106,117**	**£20,268,080**	**£102,923**	**£384,759**	**£351,299**	**£351,299**	**£3,542,184**	**£3,245,452**	**£14,387,165**	**£24,786,374**
		%	*2.0%*	*2.2%*	*70.2%*	*81.8%*	*0.7%*	*1.6%*	*2.4%*	*1.4%*	*24.6%*	*13.1%*	*100.0%*	*100.0%*
**Vietnam (target N=19)**	2	**£**	**£125,955**	**£168,071**	**£1,895,403**	**£4,024,571**	**£16,461**	**£98,330**	**£192,158**	**£192,158**	**£263,007**	**£240,493**	**£2,492,985**	**£4,723,622**
		%	*5.1%*	*3.6%*	*76.0%*	*85.2%*	*0.7%*	*2.1%*	*7.7%*	*4.1%*	*10.5%*	*5.1%*	*100.0%*	*100.0%*
	5	**£**	**£150,814**	**£227,927**	**£3,315,111**	**£6,698,987**	**£28,747**	**£141,512**	**£192,158**	**£192,158**	**£657,518**	**£601,232**	**£4,344,348**	**£7,861,817**
		%	*3.5%*	*2.9%*	*76.3%*	*85.2%*	*0.7%*	*1.8%*	*4.4%*	*2.4%*	*15.1%*	*7.6%*	*100.0%*	*100.0%*
	10	**£**	**£192,244**	**£327,689**	**£5,681,290**	**£11,156,348**	**£49,224**	**£213,483**	**£192,158**	**£192,158**	**£1,315,036**	**£1,202,465**	**£7,429,953**	**£13,092,143**
		%	*2.6%*	*2.5%*	*76.5%*	*85.2%*	*0.7%*	*1.6%*	*2.6%*	*1.5%*	*17.7%*	*9.2%*	*100.0%*	*100.0%*
	19	**£**	**£266,820**	**£507,259**	**£9,940,412**	**£19,179,598**	**£86,082**	**£343,030**	**£192,158**	**£192,158**	**£2,498,569**	**£2,284,683**	**£12,984,042**	**£22,506,729**
		%	*2.1%*	*2.3%*	*76.6%*	*85.2%*	*0.7%*	*1.5%*	*1.5%*	*0.9%*	*19.2%*	*10.2%*	*100.0%*	*100.0%*

* laboratory surveillance including isolate storage and transport and national reference laboratory

** number of sites in addition to national reference laboratory

Figure 6: Costs by function for sentinel sites (2 or 5) by level (core or extended) for Kenya

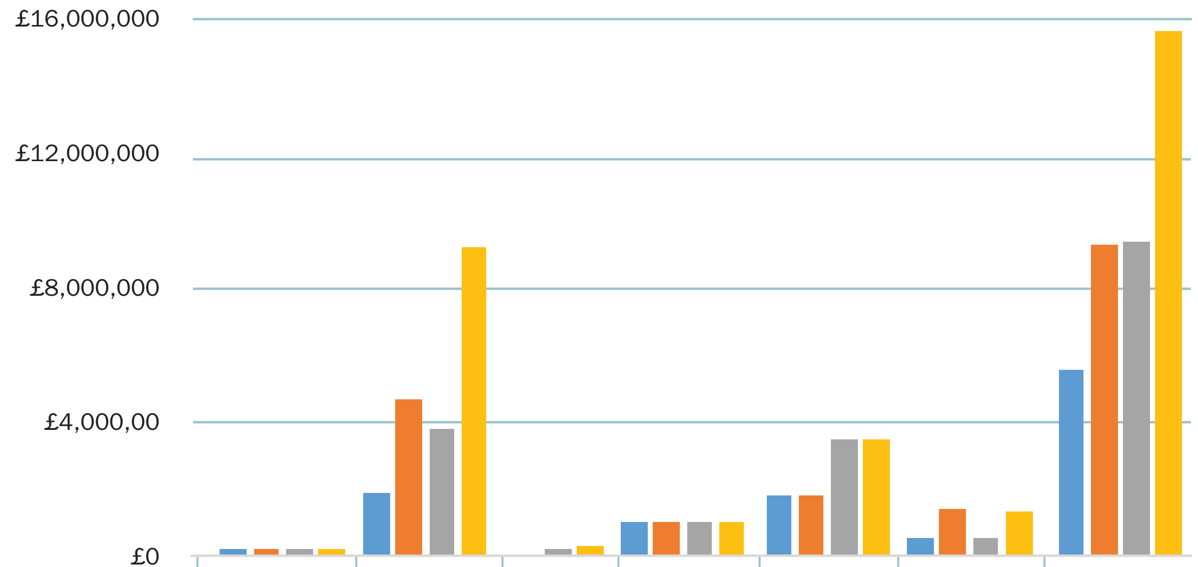

	Clinical surveilance	Laboratory surveilance incl isolate storage	Data system and review	National surveilance	National reference laboratory	Demographic surveilance	Total
Kenya: 2 Sentinel sites to level 3	£210,938	£1,869,893	£49,904	£1,032,686	£1,849,977	£580,319	£5,593,717
Kenya: 5 Sentinel sites to level 3	£242,614	£4,654,933	£67,214	£1,032,686	£1,849,977	£1,450,797	£9,298,221
Kenya: 2 Sentinel sites to level 4	£230,452	£3,820,577	£257,226	£1,032,686	£3,488,275	£535,643	£9,364,860
Kenya: 5 Sentinel sites to level 4	£265,129	£9,243,448	£304,344	£1,032,686	£3,488,275	£1,339,108	£15,672,991

Figure 7: Costs by function for sentinel sites (2, 5 or 10) by level (core or extended) for Vietnam

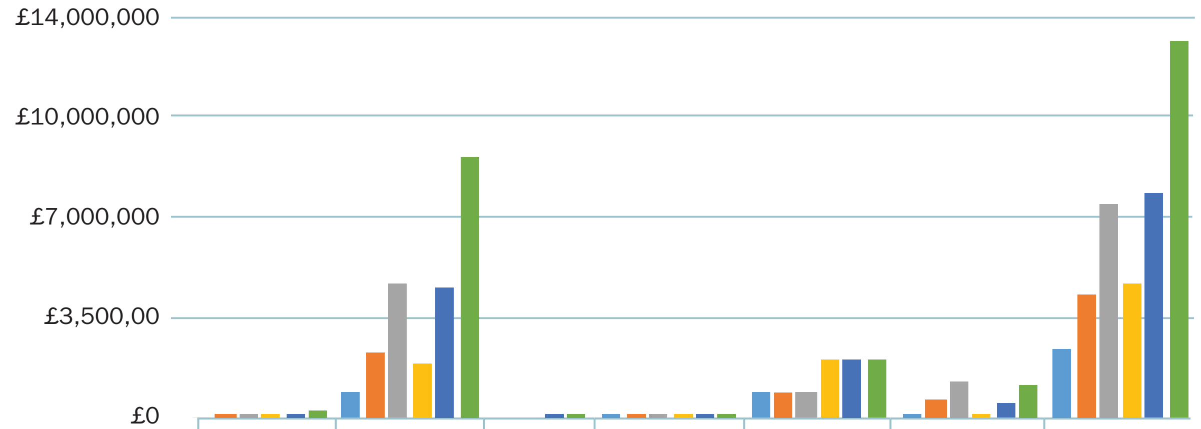

	Clinical surveilance	Laboratory surveilance incl isolate storage	Data system and review	National surveilance	National reference laboratory	Demographic surveilance	Total
Vietnam: 2 Sentinel sites to level 3	£125,995	£959,672	£16,461	£192,158	£935,732	£263,007	£2,492,985
Vietnam: 2 Sentinel sites to level 4	£168,071	£1,923,002	£98,330	£192,158	£2,101,569	£240,493	£4,723,622
Vietnam: 5 Sentinel sites to level 3	£150,814	£2,379,379	£28,747	£192,158	£935,732	£657,518	£4,344,348
Vietnam: 5 Sentinel sites to level 4	£227,927	£4,597,419	£141,512	£192,158	£2,101,569	£601,232	£7,861,817
Vietnam: 10 Sentinel sites to level 3	£192,244	£4,745,558	£49,224	£192,158	£935,732	£1,315,036	£7,429,953
Vietnam: 10 Sentinel sites to level 4	£327,689	£9,054,780	£213,483	£192,158	£2,101,569	£1,202,465	£13,092,143

Figure 8: Costs over the course of five years for surveillance at 5 sentinel sites in Kenya, Ethiopia and Vietnam at level 3 and 4

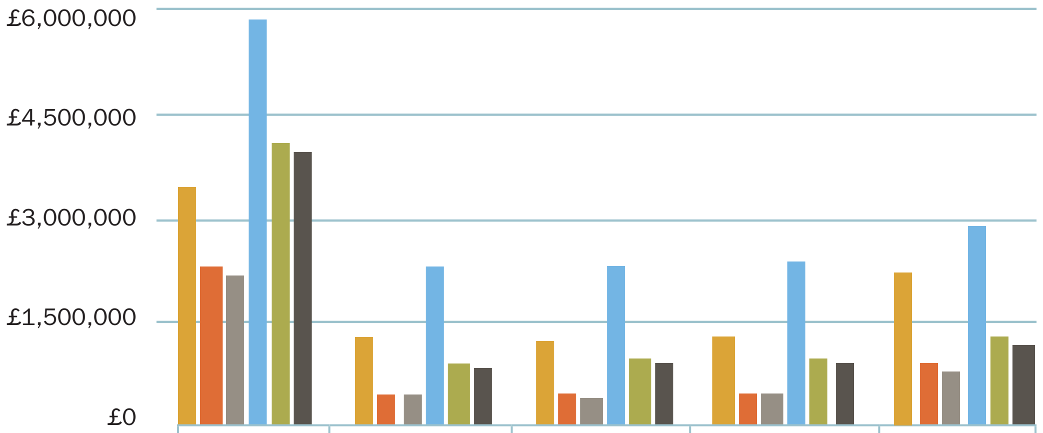

	Year 1	Year 2	Year 3	Year 4	Year 5
Kenya Level 3	£3,442,192	£1,281,751	£1,260,856	£1,325,869	£1.987,554
Ethiopia Level 3	£2,306,137	£481,523	£467,844	£511,771	£911,917
Vietnam Level 3	£2,165,784	£461,571	£488,342	£490,250	£778,401
Kenya Level 4	£5,825,007	£2,304,748	£2,286,989	£2,350,903	£2,904,344
Ethiopia Level 4	£4,117,311	£941,984	£999,216	£972,127	£1,301,198
Vietnam Level 4	£3,948,232	£888,773	£952,369	£922,159	£1,149,285

Higher costs in year 1 are due to the inclusion of the set-up costs

Figure 9: Main cost drivers of laboratory surveillance (including costs from site laboratory surveillance, sample transport & storage and national reference laboratory)

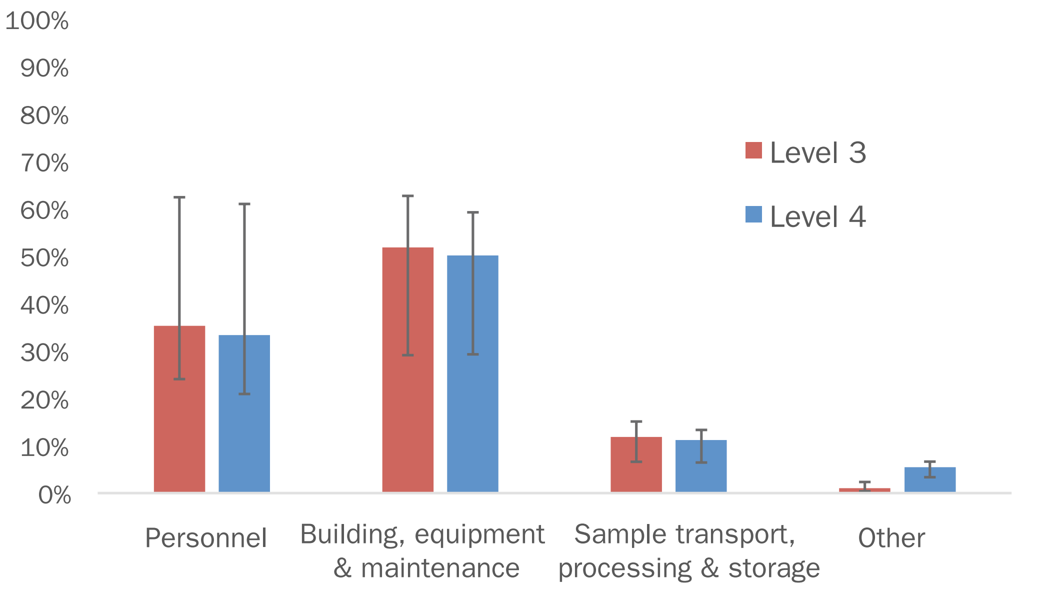

The uncertainty ranges shown for each bar represent the range of the estimate depending on country and number of sites.

8 Conclusions

We aimed to map and compare existing models for laboratory system strengthening for AMR surveillance in LMICs. The work was based on desk review and three case studies on Ethiopia, Malawi and Vietnam, as well as costing based on a site visit at KEMRI Wellcome Trust Research Programme, Kilifi, Kenya. In the settings described in sub-Saharan Africa and South-east Asia, the AMR surveillance models are **continuous, largely passive, laboratory based, using sentinel sites in country**. An **integrated model would be preferable for AMR surveillance** so that data can be interpreted in a known clinical and demographic context, and case study countries were examined with this in mind.

It was apparent that the three countries visited were very different in terms of institutional architecture, health systems and AMR surveillance capacity, even on the short site visits undertaken for this work. For example, in **Ethiopia** there was **early development of laboratory capacity in some broadly representative public hospitals**, but no linked clinical data system, or population data; in **Malawi** there was very limited laboratory capacity in public hospitals, but **a highly functioning research centre conducting surveillance** in Blantyre, with high quality laboratory data, basic clinical data and a population denominator for urban and rural Blantyre; in **Vietnam** there was a **broadly functioning laboratory capacity with a network of representative public hospitals across the country**, supported by a consortium of international collaborators and with major investment but conducted at a level of the referral hierarchy (District Hospitals) which is strongly susceptible to biases attributable to antibiotic access lower down in the referral hierarchy.

There were factors which were consistent in supporting and driving success in AMR capacity (Figure 9). These include **political commitment and collaboration** amongst and between **government and international stakeholders** to utilise local and international expertise in supporting AMR surveillance development, as well as national policies on AMR surveillance developed within **legal and ethical frameworks.** Although an assessment of interventions in relation to AMR was beyond the scope of this work, political commitment and collaboration amongst stakeholders will also be crucial when considering and implementing these interventions (e.g. stewardship and antibiotic use), and initiating policy change.

It was clear that a **functioning health system** can support development of an integrated sentinel surveillance site system, progressing perhaps to comprehensive systems, more readily than a weaker health system. The integration of AMR surveillance into a health system requires an **assessment of the form, state and functioning of a country's health system**. This is a central issue as the degree to which AMR surveillance will benefit national and local concerns (as opposed creating further burdens to staff) will largely be determined by the strength of its country's health systems.

An important component, and common limitation of **health systems** in LMICs are the **human resources** to do work, across all cadres of staff, including **clinical, laboratory, managerial, policy-making, data analysis, project management** groups. Microbiological expertise is particularly limited. There were examples of support for human capacity development in the country case studies through Universities (national and international), research institutions (national and international) and interest in developing technical postgraduate programmes.

However, where the health systems need strengthening there is **insufficient training available in country to support the development of technical capacity** to the level required. In these situations, AMR surveillance will be more likely to succeed if it is built up more slowly, **using the expertise available from the most appropriate institutions**. To ensure success at a "core level", strengthening of surveillance may require a **staged process with capacity built up over time**. Here one site would be supported (which could be through a national reference laboratory, external partner, or research laboratory) to build capacity. An example of this would be utilising the expertise available in the Malawi Liverpool - Wellcome Trust Research Programme to support AMR surveillance in a district hospital. With development of capacity to a core level at a sentinel surveillance site, this site could subsequently serve as a **training and support centre** to develop capacity at another site, thus **overcoming barriers** to high quality surveillance when capacity is thinly spread across a weak health system. From this point, over time, a network of sites could be developed, as they are being developed in Vietnam, with the potential for sub-regional reference laboratories as the network expands.

Laboratory infrastructure with the capacity for **standardised**, quality diagnostics, are important. However, they have to be supported by appropriate **logistical support** (procurement, budget management), otherwise even the most highly equipped laboratories will not be able to function. With this is mind, securing a **core set of standard activities,** which can be maintained and result in useful, interpretable data is more important in the early stages of capacity development than the use of more advanced techniques. It also requires considerably less investment, and supporting five sentinel sites to a **core** level will likely produce more generalizable data than two sites who have an **extended** capacity, for a similar investment.

Countries who have fragile and/or transitioning health systems are least able to respond to the present burden of infectious disease. Surveillance capacity depends on the existing health system and considerable investment is needed to develop laboratory infrastructure aligned to this. High level expertise in-country to provide advocacy, championing and trusted guidance to the Ministry of Health is important. Engagement with both the Ministry of Health and other national stakeholders is essential, combined with the expertise of in-country partners such as the Centers for Disease Control or academic institutions. Establishing whether these infections are treatable will inform treatment and facilitate a response; contributing to reducing the inequities in understanding disease burden worldwide.

9 References

1. Shallcross LJ, Howard SJ, Fowler T, Davies SC. Tackling the threat of antimicrobial resistance: from policy to sustainable action. *Philosophical transactions of the Royal Society of London Series B, Biological sciences* 2015; **370**(1670): 20140082.
2. O'Neill J. Antimicrobial Resistance: Tackling a crisis for the health and wealth of nations2014. amr-review.org/ (accessed 6.6.16).
3. Murray CJ, Barber RM, Foreman KJ, et al. Global, regional, and national disability-adjusted life years (DALYs) for 306 diseases and injuries and healthy life expectancy (HALE) for 188 countries, 1990-2013: quantifying the epidemiological transition. *Lancet* 2015; **386**(10009): 2145-91.
4. Boerma T, Mathers CD. The World Health Organization and global health estimates: improving collaboration and capacity. *BMC medicine* 2015; **13**: 50.
5. World Health Organization. Antimicrobial resistance: global report on surveillance 2014. www.who.int/drugresistance/documents/surveillance report/en/ (accessed 05.05.16).
6. Yong D, Toleman MA, Giske CG, et al. Characterization of a new metallo-beta-lactamase gene, bla(NDM-1), and a novel erythromycin esterase gene carried on a unique genetic structure in Klebsiella pneumoniae sequence type 14 from India. *Antimicrobial agents and chemotherapy* 2009; **53**(12): 5046-54.
7. European Centre for Disease Prevention and Control and the European Medicines Agency. The bacterial challenge: time to react. A call to narrow the gap between multidrug-resistant bacteria in the EU and the development of new antibacterial agents.2009. http://ecdc.europa.eu/en/publications/Publications/0909_TER_The_Bacterial_Challenge_Time_to_React.pdf (accessed 16.07.16).
8. Center for Disease Prevention and Control. Antibiotic Resistance Threats in the United States, 2013. http://www.cdc.gov/drugresistance/pdf/ar-threats-2013-508.pdf (accessed 27.7.16).
9. The Centre for Disease Dynamics Economics & Policy. Global Antibiotic Resistance Partnership: The State of the World's Antibiotics.2015. https://cddep.org/sites/default/files/swa_2015_final.pdf) (accessed 03.07.2016).
10. World Health Organization. Global Action Plan on Antimicrobial Resistance 2015. http://apps.who.int/iris/bitstream/10665/193736/1/9789241509763_eng.pdf?ua=1 (accessed 6.6.16).
11. Frean J, Perovic O, Fensham V, et al. External quality assessment of national public health laboratories in Africa, 2002-2009. *Bulletin of the World Health Organization* 2012; **90**(3): 191-9a.
12. Essack SY, Desta AT, Abotsi RE, Agoba EE. Antimicrobial resistance in the WHO African region: current status and roadmap for action. *Journal of public health (Oxford, England)* 2016; doi10.1093/pubmed/fdw015.
13. Opintan JA, Newman MJ, Arhin RE, Donkor ES, Gyansa-Lutterodt M, Mills-Pappoe W. Laboratory-based nationwide surveillance of antimicrobial resistance in Ghana. *Infection and drug resistance* 2015; **8**: 379-89.
14. Vlieghe E, Sary S, Lim K, et al. First National Workshop on Antibiotic Resistance in Cambodia: Phnom Penh, Cambodia, 16–18 November 2011. *Journal of Global Antimicrobial Resistance* 2013; **1**(1): 31-4.
15. Waku-Kouomou D, Esona MD, Pukuta E, et al. Strengthening laboratory capacity through the surveillance of rotavirus gastroenteritis in Central Africa: the Surveillance Epidemiologique en Afrique Centrale (SURVAC) Project. *Tropical medicine & international health : TM & IH* 2016; **21**(1): 122-30.

16. Lubogo M, Donewell B, Godbless L, et al. Ebola virus disease outbreak; the role of field epidemiology training programme in the fight against the epidemic, Liberia, 2014. *The Pan African medical journal* 2015; **22 Suppl 1**: 5.
17. World Health Organization. Comprehensive Analytical Profile: Malawi 2014. http://www.aho.afro.who.int/profiles_information/index.php/Malawi:Index. (accessed 27.7.16).
18. World Bank. Table of GDP per Capita (current US$)2016. http://data.worldbank.org/indicator/NY.GDP.PCAP.CD. (accessed 29.07.2016).
19. World Bank. Table of Health Expenditure, Total (% of GDP)2016. http://data.worldbank.org/indicator/SH.XPD.TOTL.ZS/countries. (accessed 27.07.2016).
20. World Bank. Malawi Overview, 2016. http://www.worldbank.org/en/country/malawi (accessed 27.7.16).
21. Center for Disease Prevention and Control. Global Health - Malawi. *2016. https://www.cdc.gov/globalhealth/countries/malawi/ (accessed 07.07.16)*
22. United Nations. Malawi Country Profile. 2009. http://www.mw.one.un.org/country-profile/ (accessed 29.07.2016).
23. World Bank. Health Expenditure, Public (% of Government Expenditure) *World Bank Data*, 2016. http://data.worldbank.org/indicator/SH.XPD.PUBL.GX.ZS (accessed 27.7.16).
24. World Health Organization. Health System Financing Profile by Country: Malawi, 2014. *Global Health Expenditure Database*, 2014. http://apps.who.int/nha/database/Country_Profile/Index/en (accessed 27.7.16).
25. Mueller DH, Lungu D, Acharya A, Palmer N. Constraints to implementing the Essential Health Package in Malawi. *PloS one* 2011; **6**(6): e20741.
26. World Health Organization. Country Cooperation Strategy: Malawi 2014. http://www.who.int/countryfocus/cooperation_strategy/ccsbrief_mwi_en.pdf (accessed 27.7.16).
27. Norton EB, Archibald LK, Nwanyanwu OC, et al. Clinical predictors of bloodstream infections and mortality in hospitalized Malawian children. *The Pediatric infectious disease journal* 2004; **23**(2): 145-51; discussion 51-5.
28. Makoka MH, Miller WC, Hoffman IF, et al. Bacterial infections in Lilongwe, Malawi: aetiology and antibiotic resistance. *BMC infectious diseases* 2012; **12**: 67.
29. Gordon MA, Graham SM, Walsh AL, et al. Epidemics of invasive Salmonella enterica serovar enteritidis and S. enterica Serovar typhimurium infection associated with multidrug resistance among adults and children in Malawi. *Clinical infectious diseases : an official publication of the Infectious Diseases Society of America* 2008; **46**(7): 963-9.
30. World Health Organization. Ethiopia: Health System Outcomes. *African Observatory, Regional Office for Africa*, 2014. http://www.aho.afro.who.int/profiles_information/index.php/Ethiopia:Health_system_outcomes. (accessed 27.7.16).
31. Ministry of Health Malawi. Health Surveillance Assistant Facilitator's Guide2009. https://www.advancingpartners.org/sites/default/files/malawi_health_surveillance_assistant_training_manual_facilitators_guide.pdf (accessed 27.7.16).
32. Segula D, Frosch AP, SanJoaquin M, et al. Prevalence and spectrum of illness among hospitalized adults with malaria in Blantyre, Malawi. *Malaria journal* 2014; **13**: 391.
33. Gordon MA, Walsh AL, Chaponda M, et al. Bacteraemia and mortality among adult medical admissions in Malawi–predominance of non-typhi salmonellae and Streptococcus pneumoniae. *The Journal of infection* 2001; **42**(1): 44-9.
34. Health Information Systems Program Malawi. Current Reporting Forms2011. http://www.hispmalawi.org.mw/index.php?page=pages&pid=46 (accessed 29.7.16).
35. Health Information Systems Program Malawi. National Roll Out2011. http://www.hispmalawi.org.mw/index.php?page=pages&pid=42 (accessed 27.7.16).

36. SanJoaquin MA, Allain TJ, Molyneux ME, et al. Surveillance Programme of IN-patients and Epidemiology (SPINE): implementation of an electronic data collection tool within a large hospital in Malawi. *PLoS medicine* 2013; **10**(3): e1001400.

37. Masangalawe C, Kandulu A, van Oosterhout JJ. Which health care facilities do adult Malawian antiretroviral therapy patients utilize during intercurrent illness?A cross sectional study. *BMC health services research* 2011; **11**: 345.

38. Wamai RG. Reviewing Ethiopia's Health System Development. *Japan Medical Association Journal* 2009; **52**(4).

39. World Bank. Data: Ethiopia 2016. http://data.worldbank.org/country/ethiopia. (accessed 27.07.2016).

40. World Bank. World Bank national accounts data, and OECD National Accounts data files.2016. http://data.worldbank.org/indicator/NY.GDP.PCAP.CD (accessed 27.7.16).

41. World Bank. Ethiopia Poverty Assessment 2015. https://openknowledge.worldbank.org/handle/10986/21323. (accessed 27.07.2016).

42. Center for Disease Prevention and Control. Global Health - Ethiopia2016. http://www.cdc.gov/globalhealth/countries/ethiopia/ (accessed 27.7.16).

43. United Nations Office for the Coordination of Humanitarian Affairs. Ethiopia: Administrative Map 2013. http://reliefweb.int/map/ethiopia/ethiopia-administrative-map-27-mar-2013 (accessed 27.7.16).

44. World Health Organization. Ethiopia:Service Delivery - The Health System. African Observatory, Regional Office for Africa, World Health Organisation. http://www.aho.afro.who.int/profiles_information/index.php/Ethiopia:Service_delivery_-_The_Health_System. (accessed 27.7.16).

45. World Health Organization. Health workforce 2016. http://www.who.int/hrh/workforce_mdgs/en/ (accessed 27.7.16).

46. Abera B, Kibret M, Mulu W. Knowledge and beliefs on antimicrobial resistance among physicians and nurses in hospitals in Amhara Region, Ethiopia. *BMC pharmacology & toxicology* 2014; **15**: 26.

47. Gebretekle GB, Serbessa MK. Exploration of over the counter sales of antibiotics in community pharmacies of Addis Ababa, Ethiopia: pharmacy professionals' perspective. *Antimicrobial resistance and infection control* 2016; **5**: 2.

48. Teshale A, Desta K, Mulugeta G, et al. Prevalence And Antibiotics Susceptibility Pattern Of Common Bacterial Uropathogens Isolated From Pregnant Women Attending Antenatal Care Clinic At St. Paul Hospital Millennium Medical College And Selam Health Center, Addis Ababa, Ethiopia. *International Journal Of Medical Science And Clinical Invention* 2015; **2**(8).

49. Drug Administration and Control Authority. Antimicrobial Use, Resistance And Containment Baseline Survey Syntheses Of Findings. *Ethiopia: Drug Administration and Control Authority of Ethiopia in collaboration with Management Sciences for Health, Strengthening Pharmaceutical Systems* 2009. http://www.fmhaca.gov.et/documents/AMR_Baseline_Survey.pdf (accessed 6.6.16).

50. Joshi M, Miralles M. Antimicrobial Resistance Advocacy and Containment in Ethiopia: Report of Initial Activities in February–March 2006. Submitted to the U.S. Agency for International Development by the Plus Program. Arlington, VA: Management Sciences for Health.

51. Ethiopian Food Medicine and Health Care Administration and Control Authority. National Strategic Framework For Prevention And Containment Of Antimicrobial Resistance. 2009. http://www.fmhaca.gov.et/documents/National_AMR_Strategy.pdf (accessed 27.7.16)

52. Ethiopian Food Medicine and Health Care Administration and Control Authority. Ethiopian Rural Drug Vender Formulary2011. http://www.fmhaca.gov.et/documents/RDV_Formulary.pdf (accessed 27.7.16).

53. Ministry of Health Federal Democratic Republic of Ethiopia. Pharmaceuticals Fund and Supply Agency.2012. http://www.moh.gov.et/en_GB/pfsa. (accessed 27.7.16).

54. Ministry of Health Federal Democratic Republic of Ethiopia. Health Sector Development Programme IV 2010/11 – 2014/15. Final Draft2010. http://www.nationalplanningcycles.org/sites/default/files/country_docs/Ethiopia/ethiopia_hsdp_iv_final_draft_2010_-2015.pdf (accessed 27.7.16).

55. World Health Organization. Ethiopia: Medical Products, Vaccines, Infrastructures and Equipment. *African Observatory, Regional Office for Africa, World Health Organisation*, 2014. http://www.aho.afro.who.int/profiles_information/index.php/Ethiopia:Medical_products,_vaccines,_infrastructures_and_equipment (accessed 27.7.16).

56. World Health Organization. Ethiopia: Service Delivery - The Health System. *African Observatory, Regional Office for Africa, World Health Organisation* 2014 http://www.aho.afro.who.int/profiles_information/index.php/Ethiopia:Service_delivery_-_The_Health_System (accessed 27.7.16).

57. Jima D, Wondabeku M, Alemu A, et al. Analysis of malaria surveillance data in Ethiopia: what can be learned from the Integrated Disease Surveillance and Response System? *Malaria journal* 2012; **11**: 330.

58. World Health Organization. Ethiopia: Health Information, Research, Evidence and Knowledge. 2014 http://www.aho.afro.who.int/profiles_information/index.php/Ethiopia:Health_system_outcomes (accessed 27.06.16).

59. Phalkey RK, Yamamoto S, Awate P, Marx M. Challenges with the implementation of an Integrated Disease Surveillance and Response (IDSR) system: systematic review of the lessons learned. *Health policy and planning* 2015; **30**(1): 131-43.

60. Hladik W, Shabbir I, Jelaludin A, Woldu A, Tsehaynesh M, Tadesse W. HIV/AIDS in Ethiopia: where is the epidemic heading? *Sexually transmitted infections* 2006; **82 Suppl 1**: i32-5.

61. Central Intelligence Agency. The World Factbook: Vietnam2016. https://www.cia.gov/library/publications/the-world-factbook/geos/vm.html (accessed 27.7.`6).

62. World Health Organization. WHO I Vietnam 2016. http://www.who.int/countries/vnm/en/ (accessed 29.7.2016).

63. World Bank. Country at a Glance: Vietnam 2016. http://www.worldbank.org/en/country/vietnam (accessed 27.7.2016).

64. World Bank. Poverty Reduction in Vietnam: Remarkable Progress, Emerging Challenges 2013. http://www.worldbank.org/en/news/feature/2013/01/24/poverty-reduction-in-vietnam-remarkable-progress-emerging-challenges. (accessed 27.07.2016).

65. Center for Disease Prevention and Control. Global Health - Vietnam 2016. http://www.cdc.gov/globalhealth/countries/vietnam/ (accessed 06.07.2016).

66. Ministry of Health & Health Partnership group. Strengthening Prevention and Control of Non-Communicable Diseases; Joint Annual Health Review. Hanoi, Vietnam. 2014 http://jahr.org.vn/index.php?option=com_content&view=article&id=227%3Ajahr-2014-ncd&catid=47%3Abao-cao-da-xay-dung&Itemid=83&lang=en (accessed 06.07.2016).

67. Thanh N, Tran B, Waye A, Lindholm L. Socialization of Health Care" in Vietnam: What Is It and What Are Its Pros and Cons? *Value in Health Regional Issues* 2014 doi 10.1016/j.vhri.2013.09.006.

68. Kotsila, Panagiota. "Health Is Gold" Institutional Structures and the Realities of Health Access in the Mekong Delta, Vietnam. *ZEF Working Paper Series 105* 2012. ttp://www.econstor.eu/handle/10419/88296 (accessed 06.07.2016).

69. Universal Health Coverage Partnership. Vietnam I Universal Health Coverage Partnership 2016. http://uhcpartnership.net/country-profile/vietnam-2/ (accessed 27.7.16).

70. Tran PD, Vu LN, Nguyen HT, et al. Strengthening global health security capacity–Vietnam demonstration project, 2013. *MMWR Morbidity and mortality weekly report* 2014; **63**(4): 77-80.

71. Vo Huu Thuan. Strengthening Public Health Surveillance and Response to Foodborne Outbreaks in Southern Vietnam: Finland: University of Tampere. 2016. https://uta32-kk.lib.helsinki.fi/handle/10024/98432 (accessed 06.07.16).

72. Kinh NV. Situation Analysis: Antibiotic Use and Resistance in Vietnam 2010. http://www.cddep.org/publications/situation_analysis_antibiotic_use_and_resistance_vietnam#sthash.qKIyMFuC.dpuf (accessed 06.07.2016).

73. Hoa NQ, Larson M, Kim Chuc NT, Eriksson B, Trung NV, Stalsby CL. Antibiotics and paediatric acute respiratory infections in rural Vietnam: health-care providers' knowledge, practical competence and reported practice. *TMIH* 2009; **14**(5): 546-55.

74. Thu TA, Rahman M, Coffin S, Harun-Or-Rashid M, Sakamoto J, Hung NV. Antibiotic use in Vietnamese hospitals: a multicenter point-prevalence study. *American journal of infection control* 2012; **40**(9): 840-4.

75. Nguyen KV, Thi Do NT, Chandna A, et al. Antibiotic use and resistance in emerging economies: a situation analysis for Viet Nam. *BMC public health* 2013; **13**: 1158.

76. Ministry of Health of the Socialist Republic of Vietnam GARP OUCRU- Hanoi. First Report on Antibiotic Use and Resistance in Vietnam Hospitals in 2008-2009. http://benhnhietdoi.vn/data/files/documents/Report_on_antibiotics_use_and_resistance_in_15_hospitals_in_2008-2009.pdf (accessed 27.7.16).

77. World Health Organization. Minister of Health of Viet Nam Signs Antimicrobial Resistance Pledge2013. http://www.who.int/medicines/news/amr_pledge_vietnam/en/. (accessed 29.7.2016).

78. World Health Organization. Vietnam Signs Aide-Memoire to Combat Antimicrobial Resistance'. *WHO Regional Office for the Western Pacific*, 2015. http://www.wpro.who.int/mediacentre/releases/2015/20150722/en/ (accessed 27.7.16).

79. Global Antibiotic Resistance Partnership - Vietnam National working group. Situational Analysis: Antibiotic Use and Resistance in Vietnam.2010. https://www.cddep.org/sites/default/files/vn_report_web_1_8.pdf (accessed 27.7.16).

80. Erhart A, Thang ND, Xa NX, et al. Accuracy of the health information system on malaria surveillance in Vietnam. *Transactions of the Royal Society of Tropical Medicine and Hygiene* 2007; **101**(3): 216-25.

81. Thanh DC, Hien NT, Tuan NA, et al. Brief behavioural surveys in routine HIV sentinel surveillance: a new tool for monitoring the HIV epidemic in Viet Nam. *Western Pacific surveillance and response journal : WPSAR* 2015; **6**(1): 52-4.

82. Global Health Security Agenda. Global Health Security Agenda Road Map: Vietnam 2015. https://ghsagenda.org/docs/Vietnam-GHSA-5-year-RoadMap-2015.pdf (accessed 27.7.16).

83. Pham N. Vietnam: Increased Surveillance Leads to Detection of Zika Virus. 2016. http://blogs.cdc.gov/global/2016/05/23/vietnam-increased-surveillance-leads-to-detection-of-zika-virus/ (accessed 06.07.2016 2016).

84. Hochwarter S, Cuong DD, Chuc N TK, Larsson M. Towards an Electronic Health Record System in Vietnam: A Core Readiness Assessment. *Journal of Health Informatics in Developing Countries* 2014; **8**(2).

85. Global Antibiotic Resistance Partnership (GARP). Situation analysis: Antibiotic use and resistance in South Africa. *South African Medical Journal* 2011; **101**(8).

Acknowledgements and attributions 10

This is an independent study commissioned by the Wellcome Trust and funded by Department of Health, UK. The team involved in this work included Anna C Seale (LSHTM), Coll Hutchison (LSHTM), Silke Fernandes (LSHTM), Helen Kelly (LSHTM), Nicole Stoesser (University of Oxford), Paul Turner (University of Oxford), Kara Hanson (LSHTM), Clare Chandler (LSHTM), Catherine Goodman (LSHTM), Richard Stabler (LSHTM), Brett Lowe (University of Oxford) and J Anthony G Scott (LSHTM). The report was first drafted by Coll Hutchison, Silke Fernandes, Helen Kelly, Nicole Stoesser and Anna Seale. The site visits were done by Anna Seale, Coll Hutchison, and Nicole Stoesser and (for economics) Silke Fernandes.

In Ethiopia we would particularly like to thank the following institutions and personnel: Ethiopian Public Health Institute, Addis Ababa - Dr Adugna Woyessa, American Society for Microbiology, Laboratory Capacity Development Consultant - Dr Lourens Robberts; Centers for Communicable Disease Control, Ethiopia - Dr Yared Tedla, Laboratory Officer for the PEPFAR programme; Armauer Hansen Research Institute, Addis Ababa - Dr Abraham Aseffa, Scientific Director and Taye Tolera Balcha (Director General); Haramaya University College of Health and Medical Sciences - Dr Yadeta Dessie, Dr Nega Assefa, Mr Melkamu Dedefo; Harar Regional Laboratory, Harar; Harar Hospital Laboratory, Haramaya University, Hiwot Fana Specialized Hospital, Harar.

In Malawi we would particularly like to thank the Malawi-Liverpool-Wellcome Trust Research Unit at Queen's Hospital, Blantyre including Stephen Gordon, Dean Everett, Brigitte Denis, Rebecca Lester, Jennifer Cornick, Jamilah Meghji, Melita Gordon and Nicholas Feasey.

In Vietnam, we would like to thank the staff of the Oxford University Clinical Research Unit (OUCRU), including Guy Thwaites, Rogier Van Doorn, and Bui Huyen Trang, as well as Dr Nguyen Van Vinh Chau (Director, Hospital for Tropical Diseases) in Ho Chi Minh City, and Prof Nguyen Van Kinh, Prof Nguyen Vu Trung and Dr Le Thi Hoi from the National Hospital of Tropical Diseases; Prof Luong Ngoc Khue, Ngo Thi Bich Ha, Dr Nguyen Trong Khoa, and Mr Cao Hung Thai from the Ministry of Health, Medical Services Administration (MSA).

In Kenya, we would like to thank the KEMRI-Wellcome Trust Research Programme including Neema Mturi, Shebe Mohammed, Mary Muthomi, Brett Lowe, Salim Mwarumba, Oscar Kai, Moses Mosobo, Horace Gumba, Anthony Etyang, Eduard Sanders, Elinah Kaulu, Dorothy Tole, Lucian Mshote, Barnes Kitsao, Godfried Otieno.

We would also like to thank others who gave their time and advice, including Prof Rob Heyderman, Dr Chris Parry, and the WHO Invasive Bacterial Disease group.

11 Appendices

A: Summary features of pilot surveillance study in three geographic regions in Ghana

	Current efforts	Gaps/Limitations
Study/ publication	Surveillance pilot study spanning 6 month period from June – Nov 2014	Rise in ciprofloxacin resistance over time in Ghana raises concerns about need for action
Population coverage	9/10 regions of Ghana (Upper West Region did not submit)<br><br>27% data from inpatients and 60% from outpatients; sources of remaining were unidentified.<br>Specimen type included blood, urine, stool, swabs (ear, eye, wound, etc.) and sputum<br><br>53% were female, 41% male, 6% unknown	Overrepresentation of laboratories reporting data from southern sector of Ghana (~67%), in particular from academic tertiary referral laboratories and underrepresentation from northern sector – disparities such as this may introduce biases while interpreting results to direct antibiotic policy in Ghana<br>Considerations such as lack of access to healthcare facilities in remote and rural parts in northern sector, economic and social reasons and patronage of traditional medications compared to orthodox medicine must be factored into interpretation of surveillance data.
Health care seeking		
Clinical surveillance/ Population sampled	24 centres:<br>3 teaching hospitals<br>7 regional hospitals<br>3 zonal public health reference laboratories (PHRL)<br>4 faith based hospitals<br>3 district hospitals<br>2 research laboratories<br>2 quasi-government hospitals (Military and Cape Coast university hospital)	In this lab-based surveillance, datasets were received from 18/24 participating laboratories.<br>Six laboratories did not submit any data during the surveillance period.<br>Preliminary investigations revealed lapses within these hospitals, including:<br>breakdown of culture facilities; clinicians not making request for cultures; and internal managerial issues
Training	3 day residential workshop organised to harmonise susceptibility testing protocols and discuss issues relating to logistics<br>Ghana divided into north and south regions for training<br>Training of 33 technologists from 24 laboratories, included classroom lectures and practical sessions including Clinical Laboratory Standard Institute guidelines for susceptibility testing	
Quality control	25% of laboratories routinely participate in External Quality Assurance (EQA) schemes	
Data collection	Pre-designed data collection sheet finalised, for collection of:<br>Patient information<br>Specimen type<br>Bacteria isolated<br>Antimicrobial agents tested and inhibitory zone sizes<br>Initials of technologist submitting data<br>Completed data sheets sent biweekly to Medical Microbiology Dept., School of Biomedical and Allied Health Sciences<br>All data received was entered into WHONET database files by research assistant<br>For purposes of analysis, geographical border of Ghana was divided into southern, middle and northern sectors<br>Analysis was performed using Epi Info	
Isolate identification	Bacterial isolates identified as far as possible using Gram morphology, routine biochemical tests, and in some instances the API 20E system (bioMerieux).	Most laboratories have capacity for urine culture, many lacked capacity for blood culture<br>Laboratories do not process enough blood culture<br>Gram-negative pathogens top the list of important bacteria identified in this surveillance study. Laboratories in Ghana lack capacity for anaerobic culture
Susceptibility testing	Using disk diffusion method<br>Inhibition zone sizes measured and reported in millimetres	
Management		Clinicians not making request for culture<br>Internal managerial issues<br>Data was received from only one of the public health reference laboratories (PHRL), suggesting that PHRLs in Ghana are not yet ready to coordinate surveillance system and the mandate, direction and functions of PHRLs in Ghana must be first aligned to address the global public health threat of AMR

B: Summary of AMR networks and governance in South Africa[85]

	Current effort	Gaps/Limitations
Leadership	The Global Antibiotic Resistance Partnership (GARP)* - Situation Analysis: Antibiotic Use and Resistance in South Africa report, published in conjunction with the South African Medical Journal. National Antibiotic Surveillance Forum (now the South African Society for Clinical Microbiology), the Group for Enteric, Respiratory, and Meningeal disease surveillance (GERMS), and their partners perform surveillance that is nationwide, long-term, and focused on clinically relevant pathogens and antibiotics.	
Other stakeholders: Public	The Group for Enteric Respiratory and Meningeal disease Surveillance in South Africa (GERMS-SA);<br>The National Antibiotic Surveillance Forum (NASF)/ South African Society for Clinical Microbiology (SASCM);<br>The STI Reference Centre, in collaboration with the National Department of Health (NDoH), also conducts surveillance.<br>NASF/SASCM collects data on selected invasive pathogens isolated from blood and cerebrospinal fluid specimens at academic hospitals.<br>The participating laboratories, which participate voluntarily, have been principally those serving academic tertiary care hospitals	
Stakeholders: Private	Private sector AMR data are generated through a collaborative effort involving private pathology (microbiology) laboratories that use a common laboratory system, Meditech, that enables all participants to use a standardised and reproducible means of data extraction for the generation of AMR reports.	
Policy	AMR National Strategy Framework 2014-24 which provides a structure to manage AMR to limit further increases in resistant microbial infections<br>Essential Drugs List (EDL) and Standard Treatment Guidelines (STGs).	
Population coverage	AMRRU of the NICD introduced, in July 2010, a laboratory-based AMR surveillance (LARS) system to elucidate the epidemiology of AMR HAI-associated Staphylococcus aureus and Klebsiella pneumoniae isolates collected from patients at designated sentinel sites throughout South Africa.	

	Current effort	Gaps/Limitations
Clinical surveillance/ Population sampled	GERMS-SA collects data in three areas: AIDS-related opportunistic infections, epidemic-prone diseases and vaccine-preventable diseases<br>The Enteric Diseases Reference United (EDRU) collects data on patients presenting throughout South Africa with both invasive and non-invasive diarrhoea-causing bacteria. EDRU collates patient and isolate information under a single record, compiled from 2003 onward. EDRU attempts to represent the entire country by offering free serogrouping, serotyping and antibiotic susceptibility testing to all diagnostic laboratories throughout the country.<br>The STI Reference Centre has tested Neisseria gonorrhoeae isolates for antibiotic susceptibility, collected from 270 sites across the country. It has played a leading role in the development of the Gonococcal Resistance to Antimicrobials Surveillance Programme (GRASP) in Africa, a global programme co-ordinated by the World Health Organization (WHO). It has supported isolate collection and laboratories in Namibia, Zimbabwe, Madagascar and Tanzania, providing regional technical assistance and training<br>Several important studies have also been conducted in the private sector. Currently, the Federation of Infectious Diseases Societies of Southern Africa (FIDSA) conducts surveillance for various pathogens, reported on their website	As surveillance is laboratory-based, it's not possible to correlate resistant pathogens with clinical outcomes.<br>Measurement of resistance in the community is extremely limited<br><br>There are concerns about comprehensiveness, as participation is voluntary and limited to large university health centers
Isolate identification		Non standardized collection practices
Susceptibility testing		
Data collection/ reporting	GERMS-SA produces an annual report, as well as a quarterly surveillance bulletin and numerous publications, maintaining an extensive database on antibiotic resistance.	
Quality control/ training	GERMS-SA regularly audits participating laboratories for quality and completeness. The stored isolates form can be accessed for special studies that are conducted periodically.	

	Current effort	Gaps/Limitations
Management		- Data from this surveillance must be translated into existing mechanisms for regulating medicines, including the Essential Drugs List (EDL) and Standard Treatment Guidelines (STGs)<br>- little evaluation of impact of EDL and STGs<br>- The NASF/SASCM system has strengths, but is limited by lack of clinical information on cases, variability in analytics, inability to differentiate between community- and hospital-acquired infections, the limits on population coverage, and differences in methods.

C: Laboratories assessed as part of SURVAC

Cameroon (CAE)<br>(n=5)	Yaoundé:<br>Centre Pasteur de Yaoundé,<br>The Mother and Child Center,<br>Lanacome Laboratory<br>Laboratoire National de Santé et d'Hygiene Mobile<br>Douala:<br>Laquintinie Hospital laboratory.
Democratic Republic of Congo (DRC)<br>(n=6)	Kinshasa:<br>Institut National de Recherches Biomedicales (INRB)<br>Hopital Pédiatrique Kalembelembe<br>Hopital Kingasani<br>Laboratories for disease-specific programme for Tuberculosis, HIV and Malaria<br>Lubumbashi:<br>laboratory of the Hopital General Sendwe
Central African Republic (CAR)<br>(n=5)	Bangui:<br>Pasteur Institute of Bangui<br>Laboratoire National de Santé Publique,<br>HIV Laboratory,<br>Blood Bank Laboratory<br>Clinical Laboratory of the Complex Pediatrique de Bangui Hospital

D: Findings from SURVAC (Surveillance Epidémiologique en Afrique Centrale) program for rotavirus gastroenteritis surveillance in Central Africa

Laboratory assessment	The outcome of the assessment conducted at the beginning of the implementation of SURVAC project indicated that laboratory services and infrastructure were very weak throughout SURVAC countries. In most laboratories, much equipment was broken or could not be used due to the lack of proper maintenance. Reagents and supplies were not always available and this severely affected laboratory analyses.<br>Before SURVAC, the level of rotavirus testing capacity within SURVAC countries varied. In CAE and DRC where rotavirus surveillance started in 2007 and 2009, respectively, rotavirus samples analysis was limited to EIA testing only and due to resource constraints genotyping was performed at the RRLs in South Africa and Ghana [4]. In CAR, there was no surveillance system for rotavirus before the SURVAC project. Although CAE and DRC had attended annual training workshops conducted by RRL in South Africa that included RT-PCR techniques, none of the SURVAC countries had established molecular testing for rotavirus genotyping by RTPCR in their laboratories. For several years, DRC and CAR had been conducting PCR testing for other diseases.
SURVAC achievements 2009 to 2013	The key issues were procurement of equipment, reagent and supplies; training of laboratory personnel; establishment of molecular biology techniques for rotavirus genotyping; management of laboratory data; and enrolment of SURVAC countries laboratories in the WHO PT programme for rotavirus genotyping.
Procurement of equipment, reagent and supplies	A list of equipment was established to meet the requirement to set up molecular biology techniques (RT-PCR) and/or EIA technique in SURVAC laboratories. At the beginning of the project, most equipment was provided by WHO/AFRO and reagents by CDC. To build a sustainable system for laboratory items procurement, the WHO/AFRO and WHO country offices managed this procurement since June 2012 and from June 2013, the MoH was supposed to take over the procurement of all laboratory reagents. However, some of the molecular biology reagents used in the laboratories were still provided by either CDC or procured through WHO procurement system with support from WHO country offices.
Training of laboratory personnel	The human workforce was strengthened through technical training and assistance in specialised areas. In all, 23 personnel from the three SURVAC countries were trained in biosafety, quality assurance, QC, specimen handling and transport, rotavirus diagnosis using EIA and genotyping of rotavirus strains using RT-PCR. Despite a successful implementation of training activities, there still was a challenge of staff retention as the project had to deal with the departure of key personnel involved in the surveillance of rotavirus in CAE and in DRC. This situation, mainly due to the lack of career development opportunities and low salaries, is known to be a major issue in African countries [13]. SURVAC countries are working with partners to address this issue through another component of SURVAC project, the Central Africa Field Epidemiology and Laboratory Training (CAFELT).

Laboratory testing	Prior to SURVAC intervention, none of the laboratories in SURVAC countries performed molecular genotyping testing to support rotavirus surveillance. With support from the African Rotavirus Network [15], stool samples from CAE and DRC were sent with one laboratory member annually to the RRL in South Africa, to perform rotavirus genotyping analyses. Because of the high cost of this process, only few samples (50) were analysed for rotavirus genotyping by RT-PCR each year [5]. This resulted in few samples being analysed and minimal data on rotavirus strains circulating in these countries. Since the implementation of the SURVAC project, all three countries (3/3) are now performing rotavirus genotyping testing using RT-PCR. As a result no more samples are sent abroad for rotavirus genotyping. Furthermore, they all participate in external quality control for rotavirus genotyping. Central level laboratories are now carrying out the genotyping of rotavirus samples. This has resulted in an increase in the number of samples analysed and strains genotyped per year in each country. a slight decrease of number of samples genotyped was noticed in 2012, due to reagent stock outs
Management of laboratory data	Laboratory data collection was previously paper-based and upgraded to the computerised system using a standard data collection and reporting tool provided by WHO/AFRO. The computerised system was used to submit monthly reporting of data to the MoH and WHO. Epidemiologic and laboratory data produced by the SURVAC project were successfully used by the countries to advocate for the introduction of rotavirus vaccine in their countries. As result, rotavirus vaccine was introduced into the immunisation schedule in CAE in April 2014. DRC and CAR are planning to do so in 2016.
Proficiency testing and Quality Control	Since 2012, SURVAC laboratories have been participating in the PT program for rotavirus genotyping provided by WHO and CDC. In 2012, the PT score for the EIA testing was 100% for all laboratories and genotyping scores were ranging from 56.3 to 100% in 2012 which was satisfactory performance for a first PT. In 2013, their genotyping score dropped, ranging from 53.1 to 75% for CAE and DRC, respectively. This could have been a result of the logistics and administrative hurdles of WHO/AFRO transferring the responsibility to the MoH for providing the reagents and supplies for rotavirus genotyping as recommended by SURVAC project management committee. In addition to the reagent shortage issue in CAE which prevent laboratory staff from practicing regularly genotyping analysis, the staff turnover certainly affected this proficiency test as personnel responsible for rotavirus genotyping left the laboratory and the staff who performed the test did not have sufficient rotavirus genotyping experience. CAR could not participate in the proficiency testing for genotyping in 2013 due to a political crisis that has affected the country beginning in March 2013. In fact, their PT panel box was opened before receipt in the laboratory and the genotyping primers were missing. In 2014, all SURVAC laboratories passed both EIA and genotyping PT.

E: Current status of AMR surveillance efforts in the Philippines

Leadership	Current Effort	Gaps/Limitations
Policy	No comprehensive national plan<br>There are existing Antimicrobial Resistance Surveillance Program (ARSP) for humans, but none yet in animals<br><br>Philippine Action Plan to Combat AMR: One Health Approach<br>In April 10, 2014, President Benigno Aquino III created the Interagency Committee on AMR (ICAMR)<br>Through Administrative Order (A.O.) No. 42 directing member government agencies from the health, agriculture, trade, research and local government sectors to formulate and implement a national action plan that can rationalize and streamline government efforts to combat the AMR problem.	Fragmented policies on surveillance, laboratory capacity, drug accessibility and quality, rational use of antimicrobials, infection prevention and control and research for new alternative drugs and innovative methods<br><br>No body to consolidate all these efforts into one AMR program; question of sustainability and financing
National body	The Philippines is also part of the Asian Network for Surveillance of Resistant Pathogens (ANSORP), which is a study group involving different countries in Asia in relation to AMR the Philippines is actively collaborating with the Ministry of Health Malaysia in the conduct of a rapid assessment for regulatory measures to combat AMR.	
Other stakeholders		
Infection Control	Late 1980s – establishment of Infection and Control Committees (ICC) in hospitals as per Memo issued by the Office of Hospital Facility Services and Regulation of DOH<br>1996 – emphasis on the establishment of three vital committees in their hospitals (one of which is the ICC) as per Revised Rules and Regulation on Registration and Licensing of Hospitals<br>2003 - Manual on National Standards in Infection Control for Healthcare Facilities (2009, latest version)<br>2012- Creation of the National Center for Health Facilities and Development Technical Working Group for the development of the National Policy on IPC<br>The DOH-National Center for Health Promotion (NCHP) renewed the program on Healthy Settings, which is primarily about the promotion of health that necessarily includes sanitation, in places like schools and workplaces; Emphasis given on proper handwashing	The standards, guidelines and various measures are not properly disseminated and/or not strictly enforced.<br><br>Due to lack of awareness about the implications and importance of effective IPC, and lack of expertise in the area of IPC and dedicated personnel across the country.<br>AMR surveillance is not one of the major functions of IPC programs in the hospitals.<br>In most instances, AMR surveillance is not conducted.

Leadership	Current Effort	Gaps/Limitations
Drug Law/ Policies	AO 62 s. 1989 also known as “Rules and Regulations to Implement Prescribing Requirements under the Generics Act of 1988”<br><br>AO 63. series of 1989 entitled “Rules and Regulations to Implement Dispensing Requirements under the Generics Act of 1988<br>Generics Act of 1988<br><br>Cheaper Medicines Act of 2008<br>Food and Drug Administration Act of 2009 - The intention is to strengthen the regulatory capacity to ensure the safety, efficacy and quality of all medicines for human and animal health, including addressing counterfeit medicines	Pharmaceutical products are being prescribed inappropriately.<br><br>STG non-adherence: Prescribing of broad-spectrum antibiotics and of prolonged duration of prophylaxis.
	Philippine Medicines Strategy on Access to Medicines (2011-2016)<br>• Safety, Efficacy and Quality<br>• Availability and Affordability<br>• Rational Use of Medicines<br>• Accountability, Transparency and Good Governance<br>• Health Systems Support	Dispensing of antimicrobials without prescriptions<br>• Pharmacists, who are supposed to educate the consumers, act as prescribers of antimicrobials<br>• Drugstores operating without the supervision of a pharmacist, leaving all the dispensing duties to pharmacy assistants who most likely did not have adequate training on medicines and in counselling patients
		Pervasive practice of self-medication, and purchase of antimicrobials without doctor’s prescription<br><br>Patients and caregivers generally consider antibiotics to be relatively risk-free and are often not troubled by considerations of under treatment or development of resistant organisms<br>Widespread self-treatment often with the least effective agent in an incorrect dosage<br>Local practice of recycling prescriptions and prescription sharing among friends, neighbours and relatives.<br>Widespread lack of patient awareness that drug regimens should be completed

Leadership	Current Effort	Gaps/Limitations
Drug lists	Complete Treatment Package of the DOH NCPAM<br>- Medicines (including Antibiotics) are packaged and dispensed to patients in complete dosage regimens	Development of an Administrative Order on the Implementation of RUM<br><br>• where the AMR Program is an integral component<br><br>• Target month of approval: SEPTEMBER 2013
Current surveillance programme/ pilot	The ARSP data of the RITM come from the program’s 22 sentinel sites and the National Tuberculosis Research Laboratory<br><br>• All human cases, without any correlation with those from the animal health sector<br><br>• Results of studies done in academic institutions on AMR are not incorporated in the general picture	• No correlation of laboratory-based data with clinical data or antibiotic use surveillance in the status quo<br><br>• limiting the utility of existing data.<br><br>• The extent of external quality assurance system (EQAS) implementation, particularly for units outside the existing ARSP is not clear
	The ARSP 2014 Annual Report<br>In the Philippines, this is accomplished through the AMR<br>Surveillance Program (ARSP), which was established in 1988 by the DOH through the Research Institute for Tropical Medicine (RITM), which serves as the reference laboratory and central coordinating center conducting annual external quality assessment scheme (EQAS) for 23 sentinel sites in 14 regions of the country. The sentinel sites are mandated to implement standards for culture and susceptibility, and unusual test results or patterns are sent to the AMR Surveillance Reference Laboratory (ARSRL) monthly	

Leadership	Current Effort	Gaps/Limitations
Antibiotic use in animals	There are three (3) government agencies involved in the regulation of veterinary drugs particularly antimicrobials, in the country. These are the Bureau of Animal Industry (BAI) and Bureau of Fisheries and Aquatic Resources (BFAR) of the Department of Agriculture (DA) and the Food and Drug Administration (FDA) of the Department of Health (DOH). There are limited studies conducted by the universities and research agencies on AMR in veterinary sector. A study conducted by the College of Veterinary Medicine, University of the Philippines - Los Baños (UPLB), Los Baños, Laguna revealed AMR patterns in livestock and farm environmental/wildlife animals as indicators of the use of antibiotics in Philippine agricultural practices. In this study, the common antibiotics used in each livestock commodity were determined. The different bacteria were isolated and evaluated for susceptibility and/or resistance to antimicrobial drugs	Extensive use of antimicrobials in animal husbandry as growth enhancers and prophylactic agents

F: Summary of initiatives taken to contain antibiotic resistance in Cambodia

Initiatives	Details
A comprehensive national plan for rationalising drug use	This includes as key targets: the battle against counterfeit and substandard drugs; the reduction of self-medication and over prescription; enhanced involvement of the pharmaceutical staff; the reduction of antibiotic use in agriculture.
Revision of the national clinical practice guidelines	Current guidelines were developed in 1999 and are now out of date. To tackle this problem, the MoH launched a plan to update and revise them at 5-yearly intervals and also to ensure compliance with them.
National Strategic Plan for Medical Laboratory Services	In collaboration with WHO, the Cambodian National Institute of Public Health and other partners, an external quality assurance programme and a laboratory information system were explored as well as basic requirements for surveillance of antibiotic resistance at subnational level. One of the most important problems regarding the Cambodian microbiological capacity is its extreme scarcity, and even near-absence, of the service outside the capital and larger cities.
Training/ Medical Biology curriculum	Since 2009, the University of Health Sciences re-launched, with the support of Fondation Mérieux, a 3-year Medical Biology curriculum for pharmacists and medical doctors, allowing them to obtain a 'Specialised diploma in Medical Biology'
Infection control policies	In 2010, the National Strategic Plan and Guidelines for Infection Prevention and Control in Health Care Facilities were launched. The plan and guidelines, which are backed by standardised training modules, envisage the creation of two 'Centres of Excellence' in Battambang and Kampong Cham as well as institutionalisation of infection control activities as part of the annual cycle of funding request, as well as initiating the first Cambodian 'hand hygiene day'.<br>A first assessment by the MoH revealed major problems in the management of waste and waste water and in the maintenance of basic hygiene while working in often dilapidated infrastructure.

G: Current status of AMR surveillance efforts in Thailand

Leadership	Current Effort	Gaps/Limitations
Policy	National policies relating to AMR (2011 – present)	No comprehensive AB policy at national level No audit and intervention system<br>Draft AB policy but with no implementation NARST: surveillance ABR but no link to policy
National body	National AMR Surveillance Centre, Thailand (NARST) (http://narst.dmsc.moph.go.th/) (1997 - present) & WHO collaborating center	
Other stakeholders	Health Systems Research Institute (HSRI) - support health system development by working corporately with research alliances and engagement of stakeholders including policy-decision makers, potential research users, the media and the general public Pediatric Infectious Disease Society of Thailand - professional org<br>• Infectious Disease Association of Thailand – professional org<br>• Thai Health Promotion Foundation - Autonomous state agency support activities related to risk factors to health<br>• Drug System Monitoring and Development Program – Academic, monitors rational drug use, Chulalongkorn University<br>• Center AMR Monitoring Food- borne Pathogens, Faculty of Veterinary Science, Chulalongkorn University	
Infection Control	National strategies on EID 2013 – 2016 Human side<br>National Infection Control Program<br>• EID & International Health Regulation 2005	

Leadership	Current Effort	Gaps/Limitations
Drug Law/ Policies	National drug policy & strategies 2011 regarding rational drug use & AMR	Antibiotics available in rural shops<br>• Dispensing antibiotics without prescriptions common<br>• Topical antibiotics, combination antibiotics<br>• Higher rate of AMR from antibiotics<br>• Antibiotic use in feed, farm, fishery, etc.
	Use of antibiotics: Reclassification of critically important antibiotics from non-prescription drugs to prescription drugs<br>• Prohibition of direct-to-consumer advertising of antimicrobials	
	Policies to promote rational use of antibiotics and improve infection control	
	Activities by civil society organizations<br>• bottom-up projects to promote rational use of antibiotics and to eliminate dangerous drugs from communities (2010-present)<br>• Antibiotics Awareness Day campaign (2013-present)	
	Examples: Antibiotics Smart Use (ASU) (2007-present)<br>• Antibiotics Smart Use for Children (ASU-Kids) (2012-present)	Pharmaceutical industry, distributors, regulatory enforcement, and pharmacists at drugstores remain hard to convince
Health profession education	Revising undergraduate curriculum<br>• Training prescribers and pharmacists	
Antibiotic use in animals	Prohibition of antibiotic use for growth promoter<br>• Standard farms<br>• Surveillance of antibiotic residue in animal products<br>AMR Containment & Prevention Program & An Ecohealth Approach to Develop a Strategy for the Prudent Use of Antimicrobials to Control AMR in Human, Animal, and Environmental Health in Asia' (2012-2015)	

London School of Hygiene & Tropical Medicine
Keppel Street
London WC1E 7HT
United Kingdom

Improving health worldwide

www.lshtm.ac.uk
