# Supplementary material for: Supporting surveillance capacity for antimicrobial resistance: Laboratory capacity strengthening for drug resistant infections in low and middle income countries
Source: Wellcome Open Res. 2017 Sep 26;2:91. [Version 1] doi: 10.12688/wellcomeopenres.12523.1 (PMC5686477; doi:10.12688/wellcomeopenres.12523.1)
Supplement: Peer review history

# Peer review: Referee response for version 1

**DOI:** 10.21956/wellcomeopenres.13561.r27275

**Published:** 2017-11-10

**Authors:** Laura Shallcross, Patrick Rockenschaub

**Affiliations:** 1 Institute of Health Informatics, University College London, London, UK

Thank you for the invitation to review this article.

 The authors provide a clear summary of why better surveillance systems are required to tackle AMR in LMICs and the barriers to achieving this goal in this context.

**General comments**

 Although the authors mention the need for population surveillance and collection of clinical data, this article mainly focuses on the need to strengthen laboratory capacity at local, regional and national level. In my view this provides only part of the answer, as we also need to embed systems for monitoring antibiotic usage (prescribed and over the counter) and for measuring treatment outcomes. This is particularly relevant in settings where microbiology laboratories are poorly resourced. For example, a clinical assessment of whether the patient got better might in some cases be more informative than drug susceptibility testing.

 The tables are a very useful tool to assess surveillance capacity. It would have been helpful to apply these criteria across each of the 3 countries presented to give a summary of the systems available in each setting.

 Whilst it makes sense to develop surveillance systems centered on hospitals, in the longer-term it would be helpful to include sentinel sites from community settings where the population attending may be systematically different from those who attend hospital (the authors allude to this in their manuscript).

**Introduction**

 In the Introduction the authors cite cost as the major barrier to the establishment of an AMR surveillance system in high income countries. I think this is only part of the reason. Until recently AMR surveillance was not a high priority because the steady supply of new antibiotics meant that drug resistance did not really matter – you could simply use a different drug.

**Desk-based analysis and conceptual model**

 It would be helpful if the authors provide a list of experts involved in developing the conceptual model.

 More information on a) which databases and resources were searched for articles and information and b) which references were used to derive the results. Examples were clarification is desirable include:

- The finding by the authors that in Africa “… capacity is being developed …" currently appears to be solely based on one extensive example from Ghana. Stating that capacity is developed based on 1/47 states possibly overstates the true development.
- No numbers were provided for South-East Asia, although numerical summaries are available for Africa. Numbers were provided for Africa, but not for South-East Asia. Assuming that both are based on the WHO report (in the absence of other reference except for a workshop report from Cambodia), it is unclear why no numbers can be provided for South-East Asia to facilitate comparison.
- It is unclear what data was used to identify the listed “emerging themes”. Some of the points seem to draw on the case studies, e.g. Training: “Well-trained staff are likely to move elsewhere for better salaries, …” which is featured in the case studies for Malawi and Ethiopia.
- Further, it is unclear how model options were identified and summarised. Are they theory based (no reference is given) or are they based on observation of models in the countries of interest? Are they exhaustive?

 

**Analytical framework**

 It is currently unclear how the functions, sub-functions and activities needed for AMR surveillance were identified? It would be helpful to include some more detail on how this was developed.

**Costs**

 No discussion is included on how representative and generalizable costs based on a highly functioning site in Kenya can be expected to be. This would be valuable in judging the extrapolation of costs to other countries (both those included in the study as well as others).

We have read this submission. We believe that we have an appropriate level of expertise to confirm that it is of an acceptable scientific standard.

# Peer review: Referee response for version 1

**DOI:** 10.21956/wellcomeopenres.13561.r26414

**Published:** 2017-10-18

**Authors:** Lisa M. Bebell

**Affiliations:** 1 Department of Infectious Diseases, Massachusetts General Hospital, Boston, MA, USA

The authors present their findings on an important research topic – laboratory capacity support for antimicrobial resistance work in resource-limited settings. This is an important study to help strengthen antimicrobial resistance activities worldwide. However, I think prior to publishing this work, a few details could be added and very minor reorganization would benefit the manuscript and make the authors’ statement even stronger.

**Study design: **The authors utilized a combination of article and website review (including the WHO AMR plan) and site visits to three countries to compile data for this manuscript. This seems appropriate given the current state of AMR activity in LMICs.

**Specific comments:**

**Title:** Appropriate

**Abstract:** Reflective of the introduction, methods and results.

**Introduction:** Appropriate to the topic.

**Methods: **Clear and concise. The methods description would not allow for an exact replication of the author’s work, but the nature of the work presented here does not make that a necessary. It would be helpful for the authors to note why they chose the three locations for site visits – to understand the context in which they approached their evaluation. Their costing data for all sites is based on their visit to Kenya’s site, which may not fully represent costs at the other sites.

**Results: **The authors cite evidence that only 2/47 African region countries have national AMR plans in place, and only 7/47 have ICP plans in place. However, I think it is worth acknowledging that many other countries have plans under discussion at the national level, which are anticipated to soon be in place. In addition, it may be worth mentioning that some countries are pursuing surveillance and reporting for AMR on other levels, e.g. as GARP members and through the CDDEP. This may be further reported in Supplementary File 1, but would be worth a brief mention in the main text.

 The emerging themes in strengthening lab capacity for AMR are discussed. I am surprised that poor staffing levels and lack of necessary equipment did not emerge as themes. In my experience, one of the most common complaints is that 1) there are not enough staff employed to do the work, and 2) the supplies necessary to carry out the work are not there.

 In the section on country case studies, the target audience for the author’s considerations and recommendations is not clear. Is this meant for the general reader to consider, when making surveillance plans? Is this a message to the governments of each respective country? Researchers? It would be helpful to understand better to whom these recommendations are being made, in order to ensure they reach their target.

 For the costing results, the first 2 sentences might belong better in the methods. Also, it would help the reader to understand why these assumptions (1 sentinel site per 5 million people, 5 sites per country) were made – is this standard? I think it would also be helpful to present some of the actual projected costs of starting and maintaining AMR surveillance at 1 or more sites based on the costing analysis undertaken. The current discussion is theoretical, but without showing the funds needed to begin/continue AMR surveillance, it is hard to know how much of a burden this might represent for governments or other organizations, and what it would take to get started.

**Conclusions: **The authors highlight themes for success in AMR surveillance and challenges that are common across sites, and also those that differ between sites. Here, they also acknowledge the human resource deficit that has a particularly negative impact on developing laboratory capacity for AMR surveillance, a critically important theme. I would like to see the authors give perspective on what the cost, staffing, and infrastructure thresholds would be in order for countries to adopt an AMR surveillance program. The authors discuss high-level expertise in-country and engagement with ministries of health as important steps, but they don’t discuss the costs or practical trade-offs of what this would look like in any of the countries studied, or others. It would be nice to have a more definitive recommendation or understanding of what is possible in specific places, rather than only a general overview stating the importance of engaging multiple stakeholders.

I have read this submission. I believe that I have an appropriate level of expertise to confirm that it is of an acceptable scientific standard.

# Peer review: Referee response for version 1

**DOI:** 10.21956/wellcomeopenres.13561.r26411

**Published:** 2017-10-16

**Authors:** Elizeus Rutebemberwa

**Affiliations:** 1 Makerere University School of Public Health, Makerere University College of Health Sciences, Kampala, Uganda

Thank you for giving me the opportunity to review this interesting article on; “Supporting surveillance capacity for antimicrobial resistance: Laboratory capacity strengthening for drug resistant infections in low and middle income countries”. It is interesting to read. However certain areas need to be strengthened.

1. The title is a mouthful. I may not have a better format but a mechanism to remove repeated words like ‘capacity’ may generate a more focused title.
2. One silent feature of the write-up is surveillance in animals and the environment. In this era of sustainable development goals that looks at a more comprehensive perspective of human health and actively interacting and interfacing with the environment, surveillance for antimicrobial resistance would fall short if it does not put into consideration the use of antibiotics in the environment and how these affect resistance in humans. As a scientific article, it may be helpful to refer to sources of antimicrobial resistance outside the human health care system.
3. It is good the authors talk about the relevance of governance and legislation in the conclusion. The legal framework and enforcement are key factors in antibiotic use. Some more details on this in the body would be helpful to the reader.
4. The methods section is not written in sufficient detail for the reader to replicate the study. Suffice it to say that one may need to read the report to get the details but if the manuscript is to stand on its own, more details need to be inserted.
5. The presentation may need some improvement. Tables are given and can be easily understood. However, when reports are referred to, there is no mention of the highlights from the report in the manuscript. The reader has to click onto the links and read the reports before understanding the text. If some highlights on what the reader expects to pick from the reports are put in the text, this could prime the readers to go to these reports for more detailed information.
6. The discussion seems to be merged with the results which is fine. However, an interrogation of the findings vis-à-vis current literature would be helpful. Six of the 14 references are reports. More citations from laboratory surveillance studies would help in strengthening the discussion especially that the scope is low and middle income countries which are many more than the three or four countries where data was collected.
7. This may depend on the house rules but for the reader to go systematically through the writing, one would benefit from the results and discussion being presented with respect to the objectives namely; i) To map and compare existing models for laboratory system strengthening (for AMR surveillance) and ii) To map and compare existing AMR surveillance systems in at least three countries, identifying different approaches for monitoring emergence and spread of resistance in different countries or regional settings. The results are presented according to the data collection methods and a reader would still want to put all the results together synthesize them according to the objectives stated so that they key findings coming out can be discussed with reference to other current literature.
8. The authors present an interesting conceptual framework. The use of this framework in the data collection and results comes out although not in a systematic way. However, this could be due to the way results were presented which is according to data collection methods and not the conceptual framework. If the authors abide by the house rules, that would be fine although presenting results taking into consideration the conceptual framework helps readers to follow the story better.

I have read this submission. I believe that I have an appropriate level of expertise to confirm that it is of an acceptable scientific standard, however I have significant reservations, as outlined above.
